# Supplementary material for: Dual-role iron species in photoelectrocatalytic radical trifluoromethylation with trifluoroacetates
Source: Nat Commun. 2026 Feb 20;17:2983. doi: 10.1038/s41467-026-69922-y (PMC13035886; doi:10.1038/s41467-026-69922-y)
Supplement: Supplementary file 1 — Supplementary Information [file 41467_2026_69922_MOESM1_ESM.pdf]

Supplementary Information for

Dual-Role Iron Species in Photoelectrocatalytic Radical Trifluoromethylation with Trifluoroacetates

Sara Fernández-García,‡[a] Sara Cuadros,‡[b] Irene Bosque,*[b] Jose C. Gonzalez-Gomez,*[b] Francisco Juliá-Hernández,*[a]

[a] *Facultad de Química, Centro Multidisciplinar Pleiades-Vitalis, Universidad de Murcia Campus de Espinardo, 30100, Murcia (Spain)*

[b] *Instituto de Síntesis Orgánica (ISO) and Departamento de Química Orgánica, Universidad de Alicante Apdo. 99, 03080, Alicante (Spain)*

*Corresponding author: irene.bosque@ua.es, josecarlos.gonzalez@ua.es
francisco.julia@um.es

TABLE OF CONTENTS

General considerations 3

Optimization of the reaction conditions 5

General procedure for screening reactions 5

General reaction procedure 11

Substrate scope 13

Scale-up reactions 28

General procedure for scale-up reactions 28

Unsuccessful and low-yielding substrates 30

UV-Vis absorption measurements 31

Cyclic voltammetry measurements 38

Hydrogen detection 52

Evaluation of the reactivity at different applied anode potentials 54

Attempt at the direct photoelectrotrifluoromethylation with trifluoroacetic acid 56

In-situ HRMS investigation of the reaction mixture 57

Analysis of the photodecarboxylation of Fe(III) species 63

NMR spectral data 68

References 112

GENERAL CONSIDERATIONS

Material and reagents

Commercially available reagents were purchased from Sigma-Aldrich, Acros Organic, Alfa Aesar and/or Fluorochem and used directly without purification. Anhydrous MeCN was purchased from Acros Organics and Sigma Aldrich as extra dry and 99.9+% purity. $CDCl_3$ was purchased from Sigma Aldrich and Acros Organic and stored with sodium carbonate. The solvents used in column chromatography were obtained from commercial suppliers and used without further purification.

Chromatography

Thin layer chromatography (TLC) was carried out on 0.25 mm Merck silica plates (60F-254) and on 0.20 mm Polygram® aluminum oxide neutral plates using UV light (λ = 254, 365 nm) as visualizing agent as well as potassium permanganate stains. Flash column chromatography was performed using Merck flash silica gel (particle size 0.043-0.063 mm) and Thermo Fisher Aluminum oxide basic Brockmann I (particle size 40-300 µm, pore size 60A). Dry-loading was used to introduce the sample into the column using Celite® as supporting material.

Analytical methods

Gas chromatography was performed on an Agilent 5973 mass spectrometer coupled to an Agilent 6890N gas chromatograph. High-resolution mass spectra (HRMS) were determined using an Agilent 1920 Infinity II HPLC module, an Agilent Q-TOF 7250B, and an Agilent Q-TOF 6550 hybrid mass spectrometer with JetStream electrospray + i-Funnel ionization source or an Agilent 7200 Q-TOF in electron impact mode (70 eV). NMR spectra for the characterization of compounds were recorded on two Bruker instruments: 600 MHz (^{1} H) and at 151 MHz (^{13}C), 400 MHz (^{1} H) and at 101 MHz (^{13}C) and 376 MHz (^{19}F) and 300MHz (^{1} H) at 25 ºC. Chemical shifts (δ) are reported in ppm, using the residual solvent peak in $CDCl_3$ (^{1}H = 7.26 and ^{13}C = 77.16 ppm), DMSO-d^6 (^{1}H = 2.50, ^{13}C = 39.52). Coupling constants, J, are reported in hertz. All ^{13}C NMR spectra were obtained with ^{1}H decoupling.

Reaction Set up.

Photoelectrocatalytic reactions were carried out using a set up consisting of one 75W violet 390 nm LED Kessil lamp, ElectraSyn 2.0, and cooled down using a fan (Fig. S1). Reaction vials are placed at 3 cm distance from the light source.

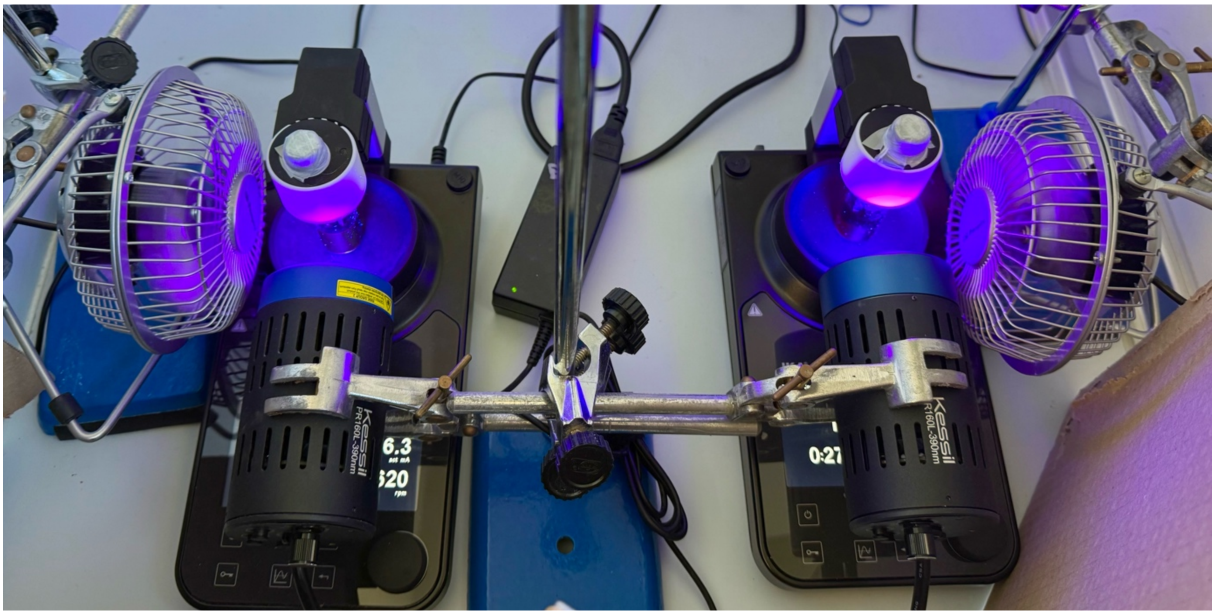

Fig. S1. Reaction setup.

OPTIMIZATION OF REACTION CONDITIONS

General procedure for screening reactions

A 10 mL ElectraSyn vial equipped with a stirring bar was charged with the corresponding substrate (0.5 mmol, 1 equiv), NaO_2CCF_3 (408 mg, 3.0 mmol, 6 equiv), $Fe(OTf)_2$ (17.8 mg, 0.05 mmol, 10 mol%), 4,4'-dimethoxy-2,2'-bipyridine **L1** (10.6 mg, 0.05 mmol, 10 mol%) and $TBAPF_6$ (387 mg, 1.0 mmol, 2 equiv.). The electrodes RVC (+)/SS (-) were then inserted, and the vial closed with the IKA screw-cap. The hole of the screw-cap was closed with a septum, and the vial was evacuated and backfilled with nitrogen with the aid of a needle, and this procedure was repeated three times. Against a positive N_2 flow, dry MeCN (5 mL) and 50µL of TFA were added *via* a syringe. Additional degassing with N_2 flow for 3 minutes was carried out in the reaction mixture. The reaction was stirred (650 rpm) under potentiostatic conditions (*i.e.* constant voltage = 2.06 V) using ElectraSyn 2.0 while irradiated with a 390nm Kessil LED, at 3 cm, and cooled down using a fan. After 24h, the electrodes were rinsed with EtOAc (10 mL) and the reaction was quenched with saturated aqueous $NaHCO_3$ solution (2 mL). An aliquot of the organic phase was analyzed by ^{19}F NMR spectroscopy using hexafluorobenzene as an internal standard to determine the yield of **2**. ^{19}F NMR spectra were recorded with D1 = 7 s to ensure reproducibility in the quantification of crude NMR yields.

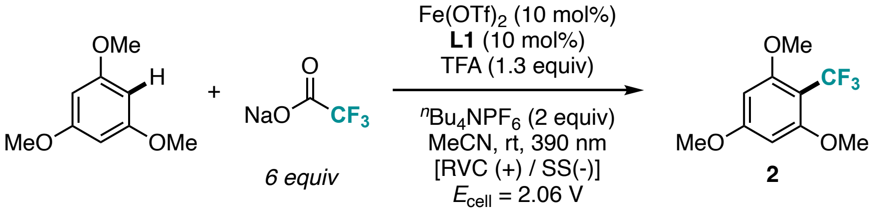

Entry	Variation	Yield (%) of **2**[a]
1	---	**76**
2	without Fe salt	0
3	without **L1**	0
4	without **L1** / with ferrocene (10 mol%)	90(9)[c]
5	without **L1** / with acetylferrocene	38
6	without **L1** / with Fe(**L1**)$_3$(PF$_6$)$_2$ (3mol%)	41[b]
7	without **L1** / with [(4-BrC$_6$H$_4$)$_3$N]SbCl$_6$	58
8	without light	0
9	without electricity	0

Table S1. Control experiments. Reaction conditions: 1,3,5-trimethoxybenzene (50.5 mg, 0.30 mmol, 1.0 equiv), NaO_2CCF_3 (245 mg, 3.0 mmol, 6 equiv), $Fe(OTf)_2$ (10.6 mg, 0.03 mmol, 10 mol%), 4,4'-dimethoxy-2,2'-bipyridine **L1** (6.5 mg, 0.03 mmol, 10 mol%) and $TBAPF_6$ (232 mg, 0.6 mmol, 2 equiv.), TFA (30 µL, 0.4 mmol, 1.3 equiv), additive (10 mol%), RVC anode, stainless steel cathode, in 3 mL ACN were stirred (650 rpm) at 35 ºC under potentiostatic conditions (i.e. constant voltage = 2.06 V) using ElectraSyn 2.0 while irradiated with a 390nm Kessil LED at 3 cm. [a]Yields were determined by ^{19}F NMR using hexafluorobenzene as internal standard. [b]7 mol% of $Fe(OTf)_2$ instead of 10 mol%. [c]yield of bistrifluoromethylated product in parenthesis.

Although the use of ferrocene as a redox mediator afforded a very good yield for the formation of product **2**, the reaction conditions in entry 1 were generally more effective for the remaining substrates in the reaction scope.

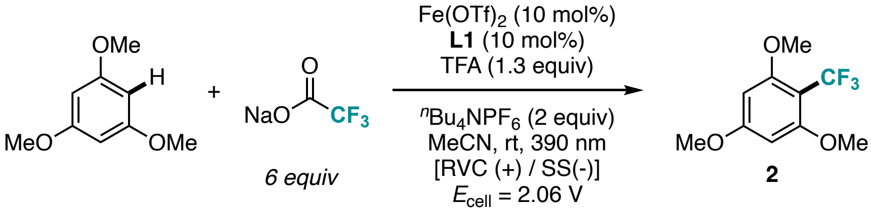

Entry	Variation	Yield (%) of **2**[a]
1	---	**76**
2	constant current (i = 2 mA)	38
3	GC instead of RVC	10
4	Gf instead of RVC	<5
5	Ni instead of SS	69(23)[b]
6	HFIP instead of TFA	18
7	PTSA (1.3 equiv)	62
8	1 equiv of ^{n}Bu$_4$NPF$_6$	24
9	DMF instead of MeCN	54(<5)[b]

Table S2. Deviation from optimized conditions. Reaction conditions: 1,3,5-trimethoxybenzene (50.5 mg, 0.30 mmol, 1.0 equiv), NaO_2CCF_3 (245 mg, 3.0 mmol, 6 equiv), $Fe(OTf)_2$ (10.6 mg, 0.03 mmol, 10 mol%), 4,4'-dimethoxy-2,2'-bipyridine **L1** (6.5 mg, 0.03 mmol, 10 mol%) and ^{n}Bu$_4$NPF$_6$ (232 mg, 0.6 mmol, 2 equiv.), TFA (30µL, 0.4 mmol, 1.3 equiv), RVC anode, stainless steel cathode, in 3 mL ACN were stirred (650 rpm) at 35ºC under potentiostatic conditions (i.e. constant voltage = 2.06 V) using ElectraSyn 2.0 while irradiated with a 390nm Kessil LED at 3 cm. [a]Yields were determined by ^{19}F NMR using hexafluorobenzene as internal standard. [b]Yield of bistrifluoromethylated compound.

The reaction was conducted in two different laboratories (University of Murcia and University of Alicante), obtaining reproducible results for the optimized conditions, particularly using reaction conditions from entry 1. Moreover, in addition to Kessil LEDs (390 nm), EvoluChem® LEDs (405 nm) afforded similar results.

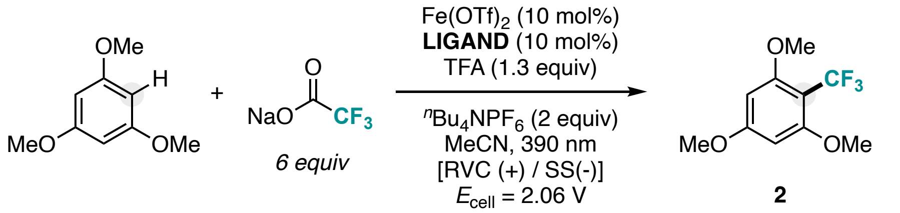

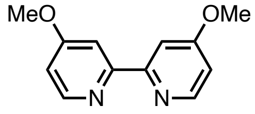

L1

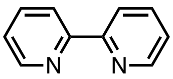

L2

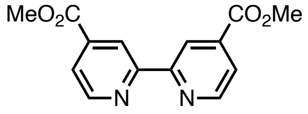

L3

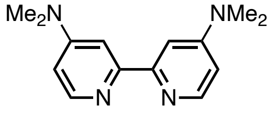

L4

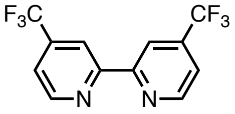

L5

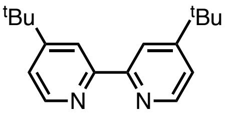

L6

Entry	Ligand	Yield (%) of **2**[a]
1	**L1**	**76**
2	**L2**	63
3	**L3**	52
4	**L4**	0
5	**L5**	46
6	**L6**	29

Table S3. Ligand screening. Reaction conditions: 1,3,5-trimethoxybenzene (50.5 mg, 0.30 mmol, 1.0 equiv), NaO_2CCF_3 (245 mg, 3.0 mmol, 6 equiv), $Fe(OTf)_2$ (10.6 mg, 0.03 mmol, 10 mol%), Ligand (10 mol%) and $TBAPF_6$ (232 mg, 0.6 mmol, 2 equiv.), TFA (30 µL, 0.4 mmol, 1.3 equiv), additive (10 mol%), RVC anode, stainless steel cathode, in 3 mL ACN were stirred (650 rpm) at 35 °C under potentiostatic conditions (i.e. constant voltage = 2.06 V) using ElectraSyn 2.0 while irradiated with a 390nm Kessil LED at 3 cm. [a]Yields were determined by ^{19}F NMR using hexafluorobenzene as internal standard.

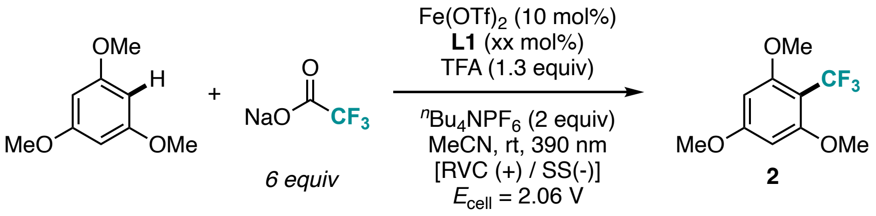

Entry	**L1** Loading (mol%)	Yield (%) of **2**[a]
1	0	0
2	10	**76**
3	30	90(9)[b]
4	50	36

Table S4. L1 Loading. Reaction conditions: 1,3,5-trimethoxybenzene (50.5 mg, 0.30 mmol, 1.0 equiv), NaO_2CCF_3 (245 mg, 3.0 mmol, 6 equiv), $Fe(OTf)_2$ (10.6 mg, 0.03 mmol, 10 mol%), 4,4'-dimethoxy-2,2'-bipyridine **L1** (xx mol%) and $^{n}Bu_4NPF_6$ (232 mg, 0.6 mmol, 2 equiv.), TFA (30µL, 0.4 mmol, 1.3 equiv), RVC anode, stainless steel cathode, in 3 mL ACN were stirred (650 rpm) at 35ºC under potentiostatic conditions (i.e. constant voltage = 2.06 V) using ElectraSyn 2.0 while irradiated with a 390nm Kessil LED at 3 cm. [a]Yields were determined by ^{19}F NMR using hexafluorobenzene as internal standard. [b]Yield of bistrifluoromethylated compound.

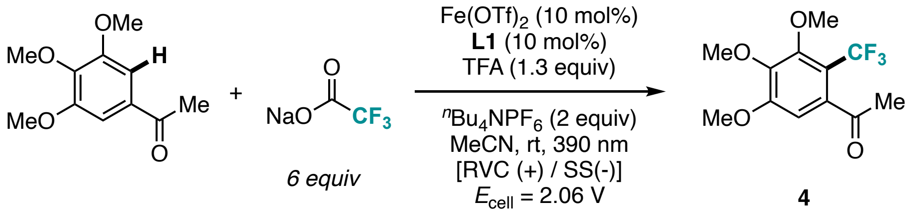

Entry	Variation	Yield (%) of **4**
1	---	**89**
2	without TFA	0
3	TFA (2.5 equiv)	59
4	TFA (5.0 equiv)	55
5	TFA (4.0 eq.) + Cs_2CO_3 (2.0 eq.)[a]	85
6	$MeCO_2H$ (1.3 equiv.) instead of TFA	21%

Table S5. Optimization of the trifluoroacetic acid additive. Reaction conditions: 1-(3,4,5-trimethoxyphenyl)ethan-1-one (63.1 mg, 0.5 mmol), NaO_2CCF_3 (245 mg, 3.0 mmol, 6 equiv), $Fe(OTf)_2$ (10.6 mg, 0.03 mmol, 10 mol%), 4,4'-dimethoxy-2,2'-bipyridine **L1** (6.5 mg, 0.03 mmol, 10 mol%) and $TBAPF_6$ (232 mg, 0.6 mmol, 2 equiv.), TFA, RVC anode, stainless steel cathode, in 3 mL ACN were stirred (650 rpm) at 35ºC under potentiostatic conditions (i.e. constant voltage = 2.06 V) using ElectraSyn 2.0 while irradiated with a 390nm Kessil LED at 3 cm. Yields were determined by ^{19}F NMR using hexafluorobenzene as internal standard. TFA = trifluoroacetic acid; PTSA = *p*-toluenesulfonic acid. [a]TFA(4.0 equiv) and C_2CO_3 (2.0 equiv) instead of TFA (1.3 equiv) and NaO_2CCF_3 (6 equiv).

GENERAL REACTION PROCEDURE

General procedure A

A 10 mL ElectraSyn vial equipped with a stirring bar was charged with the corresponding substrate (0.5 mmol, 1 equiv), NaO_2CCF_3 (408 mg, 3.0 mmol, 6 equiv), $Fe(OTf)_2$ (17.8 mg, 0.05 mmol, 10 mol%), 4,4'-dimethoxy-2,2'-bipyridine **L1** (10.6 mg, 0.05 mmol, 10 mol%) and $TBAPF_6$ (387 mg, 1.0 mmol, 2 equiv.). The electrodes RVC (+)/SS (-) were then inserted, and the vial was closed with an IKA screw-cap. The hole of the screw-cap was closed with a septum, and the vial was evacuated and backfilled with nitrogen with the aid of a needle, and this procedure was repeated three times. Against a positive N_2 flow, dry MeCN (5 mL) and 50μL of TFA were added *via* a syringe. Additional degassing with N_2 flow for 3 minutes was carried out in the reaction mixture. The reaction was stirred (650 rpm) under potentiostatic conditions (*i.e.* constant voltage = 2.06 V) using ElectraSyn 2.0 while irradiated with a 390nm Kessil LED, at 3 cm, and cooled down using a fan. After 24h, the electrodes were rinsed with Et_2O (10 mL), the reaction was quenched with saturated aqueous $NaHCO_3$ solution (2 mL) and transferred to a separating funnel. The two phases were separated, and the organic layer was washed with a saturated aqueous $NaHCO_3$ solution (2x15mL), then with brine (15 mL) and dried over anhydrous Na_2SO_4. After removal of the solvent under reduced pressure, the crude mixture was purified by flash column chromatography on a silica gel column to afford the trifluoromethylated product.

General procedure B

A 10 mL ElectraSyn vial equipped with a stirring bar was charged with the corresponding substrate (0.5 mmol, 1 equiv), NaO_2CCF_3 (408 mg, 3.0 mmol, 6 equiv), $Fe(OTf)_2$ (17.8 mg, 0.05 mmol, 10 mol%), 4,4'-dimethoxy-2,2'-bipyridine **L1** (10.6 mg, 0.05 mmol, 10 mol%) and $TBAPF_6$ (387 mg, 1.0 mmol, 2 equiv.). The electrodes RVC (+)/SS (-) were then inserted, and the vial was closed with an IKA screw-cap. The hole of the screw-cap was closed with a septum, and the vial was evacuated and backfilled with nitrogen with the aid of a needle. This procedure was repeated three times. Against a positive N_2 flow, dry MeCN (5 mL) and 50μL of TFA were added *via* a syringe. Additional degassing with N_2 flow for 3 minutes was carried out in the reaction mixture. The reaction was stirred (650 rpm) under potentiostatic conditions (*i.e.* constant voltage = 2.06 V) using ElectraSyn 2.0 while irradiated with a 390nm Kessil LED, at 3 cm, and cooled down using a fan. After 24h, the electrodes were rinsed with EtOAc (10 mL),

the reaction was quenched with saturated aqueous $NaHCO_3$ solution (2 mL) and transferred to a separating funnel. The two phases were separated, the organic layer was washed with saturated aqueous $NaHCO_3$ solution (2x15mL), then brine (15 mL) and dried over anhydrous Na_2SO_4. After removal of the solvent under reduced pressure, the crude mixture was purified by flash column chromatography on a silica gel column to afford the trifluoromethylated product.

General procedure C

A 10 mL ElectraSyn vial equipped with a stirring bar was charged with the corresponding substrate (0.5 mmol, 1 equiv), NaO_2CCF_3 (408 mg, 3.0 mmol, 6 equiv), $Fe(OTf)_2$ (17.8 mg, 0.05 mmol, 10 mol%), 4,4'-dimethoxy-2,2'-bipyridine **L1** (10.6 mg, 0.05 mmol, 10 mol%) and $LiClO_4$ (106.4 mg, 1.0 mmol, 2 equiv.). The electrodes RVC (+)/SS (-) were then inserted, and the vial was closed with an IKA screw-cap. The hole of the screw-cap was closed with a septum, and the vial was evacuated and backfilled with nitrogen with the aid of a needle, and this procedure was repeated three times. Against a positive N_2 flow, dry MeCN (5 mL) and 50μL of TFA were added *via* a syringe. Additional degassing with N_2 flow for 3 minutes was carried out in the reaction mixture. The reaction was stirred (650 rpm) under potentiostatic conditions (*i.e.* constant voltage = 2.06 V) using ElectraSyn 2.0 while irradiated with a 390nm Kessil LED, at 3 cm, and cooled down using a fan. After 24h, the electrodes were rinsed with MeCN (10 mL) and the reaction was quenched with Na_2CO_3 (54 mg) and the crude mixture was stirred for 5 min. Later, the reaction crude was filtered through celite, and after removal of the solvent under reduced pressure, the crude mixture was purified by flash column chromatography on a silica gel column to afford the trifluoromethylated product.

SUBSTRATE SCOPE

1,3,5-trimethoxy-2-(trifluoromethyl)benzene (2)

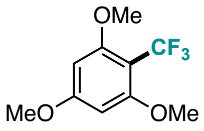

2

Prepared following the general procedure B and starting from 1,3,5-trimethoxybenzene (71.5 mg, 0.5 mmol). The crude mixture was purified by flash column chromatography on a silica gel column, eluting with a gradient from 10:0 to 6:4 (Hexane: EtOAc), to provide **2** as a white solid (71.5 mg, 61% yield).

Rf = 0.30 (Hexane/EtOAc 8:2 (v/v)).
^{1}H NMR (400 MHz, CDCl$_3$): δ 6.13 (s, 2H), 3.84 (s, 6H), 3.83 (s, 3H) ppm.
^{13}C NMR (101 MHz, CDCl$_3$) δ 163.7, 160.6 (q, *J* = 1.5 Hz), 124.5 (q, *J* = 273.2 Hz), 100.6 (q, *J* = 30.2 Hz), 91.4, 56.4, 55.5 ppm.
^{19}F NMR (376 MHz, CDCl$_3$): δ – 54.16 ppm.
HRMS (EI, *m/z*) calcd. For $C_{10}H_{11}F_3O_3$ [$M^{\bullet+}$]: 236.0655; found 236.0658.
Spectroscopic data are in agreement with previously reported literature data.[1]

1,2-dimethoxy-4-methyl-3-(trifluoromethyl)benzene (3)

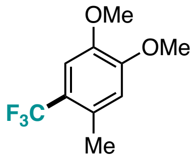

3

Prepared following the general procedure B and starting from 1,2-dimethoxy-4-methylbenzene (72 µL, 0.5 mmol). The crude mixture was purified by flash column chromatography on a silica gel column using a mixture of Cyclohexane/EtOAc (6:1) to provide **3** as a light-yellow oil (70.5 mg, 64% yield).

Rf = 0.40 (Cyclohexane/EtOAc 6:1 (v/v)).
^{1}H NMR (400 MHz, CDCl$_3$): δ 7.07 (s, 1H), 6.73 (s, 1H), 3.90 (s, 3H), 3.88 (s, 3H), 2,42 (q, *J* = 1.6Hz, 3H) ppm.

^{13}C NMR (101 MHz, CDCl$_3$) δ 151.0, 146.6, 129.9 (q, *J* = 2.0Hz), 124.8 (q, *J* = 272.7Hz), 120.8 (q, *J* = 30.5Hz)114.6, 109.2 (q, *J* = 5.7Hz), 56.3, 56.1, 19.1 (q, *J* = 2.1Hz) ppm.

^{19}F NMR (376 MHz, CDCl$_3$): δ – 60.04 (d, *J* = 2.0 Hz) ppm.

HRMS (ESI, *m/z*) calcd. For $C_{10}H_{11}F_3O_2$ [M+]: 220.0711; found 220.0706.

Spectroscopic data is in agreement with previously reported literature data.[1]

1-(3,4,5-trimethoxy-2-(trifluoromethyl)phenyl)ethan-1-one **(4)**

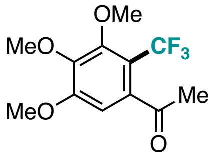

4

Prepared following the general procedure B and starting from 1-(3,4,5-trimethoxyphenyl)ethan-1-one (105.1 mg, 0.5 mmol). The crude mixture was purified by flash column chromatography on a silica gel column using a mixture of Cyclohexane/EtOAc (4:1) to provide **4** as a light yellow oil (115 mg, 82% yield).

Rf = 0.46 (Cyclohexane/EtOAc (4:1) (v/v)).

^{1}H NMR (400 MHz, CDCl$_3$): δ 6.47 (s, 1H), 3.95 – 3.94 (m, 3H), 3.89 – 3.87 (m, 6H), 2.47 – 2.46 (m, 3H) ppm.

^{13}C NMR (101 MHz, CDCl$_3$) δ 202.5, 156.3, 152.8 (q, *J* = 2.0Hz), 143.4, 137.7, 123.4 (q, *J* = 273.1Hz), 112.9 (q, *J* = 31.2 Hz), 104.4, 61.9, 60.9, 56.3, 31.5 (q, *J* = 3.0Hz) ppm.

^{19}F NMR (376 MHz, CDCl$_3$): δ –55.02 ppm.

HRMS (ESI, *m/z*) calcd. For $C_{12}H_{13}F_3O_4$ [M+]: 278.0766; found 278.0763.

Spectroscopic data is in agreement with previously reported literature data.[1]

***1,3,5-trimethyl-2-(trifluoromethyl)benzene* (5)**

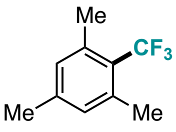

5

Starting from mesitylene (70 µL, 60.1 mg, 0.5 mmol) and following the general procedure A to provide the product **5** (70%, ^{19}F NMR yield). Isolation of the product could not be performed effectively due to volatility.

^{19}F NMR (376 MHz, CDCl$_3$): δ –53.70 ppm.

HRMS (EI, *m*/*z*) calcd. For $C_{10}H_{11}F_3$ [$M^{•+}$]: 188.0807; found 188.0809.

Spectroscopic data are in agreement with previously reported literature data.[1]

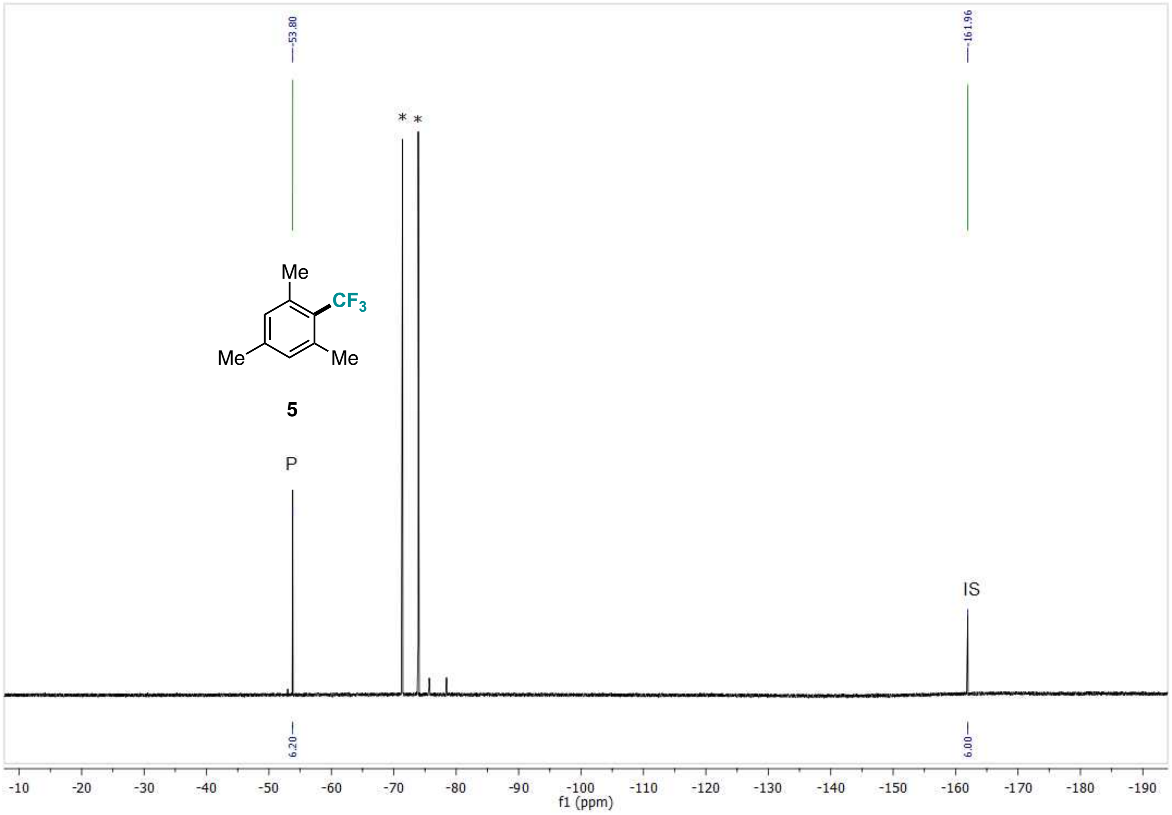

Fig. S2. ^{19}F NMR spectrum of the trifluoromethylation of mesitylene, showing the formation of 5. Yield is determined using hexafluorobenzene as internal standard (20 µL, 0,17 mmol). Signals of TBAPF$_6$ are indicated with an astherisc (*).

***1,2,4,5-tetramethyl-3-(trifluoromethyl)benzene* (6)**

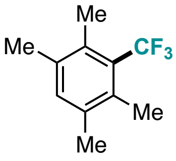

6

Prepared following the general procedure B and starting from durene (67.1 mg, 0.5 mmol). The crude mixture was purified by flash column chromatography on a silica gel column eluting with hexane to provide **6** as a white solid (43.8 mg, 40% yield).

Rf = 0.45 (Hexane).

^{1}H NMR (400 MHz, CDCl$_3$): δ 7.09 (s, 1H), 2.32 (q, *J* = 3.3 Hz, 6H), 2.26 (s, 6H) ppm.

^{13}C NMR (101 MHz, CDCl$_3$) δ 135.1, 134.5, 133.5 (q, *J* = 2.1 Hz), 128.1 (q, *J* = 22.7 Hz), 126.3 (q, *J* = 277.1 Hz), 20.5, 16.6 (q, *J* = 4.9 Hz) ppm.

^{19}F NMR (376 MHz, CDCl$_3$): δ –51.76 ppm.

HRMS (ESI, *m*/*z*) calcd. For $C_{11}H_{13}F_3$ [$M^{\bullet+}$]: 202.0964; found 202.0965.

Spectroscopic data are in agreement with previously reported literature data.[2]

***4-(tert-butyl)-1-methoxy-2-(trifluoromethyl)benzene* (7)**

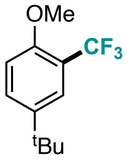

7

Prepared following the general procedure B and starting from 4-*tert*-butylanisole (88 µL, 0.5 mmol). The crude mixture was purified by flash column chromatography on a silica gel column, eluting with a gradient from 10:0 to 6:4 (Hexane: EtOAc), to provide **7** as a yellow oil (76.6 mg, 66% yield).

Rf = 0.40 (Hexane).

^{1}H NMR (400 MHz, CDCl$_3$): δ 7.58 (d, *J* = 2.3 Hz, 1H), 7.51 (dd, *J* = 8.7, 2.2 Hz, 1H), 6.95 (d, *J* = 8.7 Hz, 1H), 3.89 (s, 3H), 1.33 (s, 9H) ppm.

^{13}C NMR (101 MHz, CDCl$_3$) δ 155.4, 143.1, 130.1, 124.1 (q, *J* = 270.8 Hz), 124.1 (q, *J* = 5.3 Hz), 118.2 (q, *J* = 30.3 Hz), 111.9, 56.1, 34.3, 31.5 ppm.

^{19}F NMR (376 MHz, CDCl$_3$): δ –62.13 ppm.

HRMS (ESI, *m/z*) calcd. For $C_{12}H_{15}F_3O$ [$M^{\bullet+}$]: 232.1070; found 232.1069.
Spectroscopic data are in agreement with previously reported literature data.[3]

***1-Methyl-3-(trifluoromethyl)pyridin-2(1H)-one* (8)**

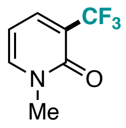

8

Prepared following the general procedure B and starting from *N*-methyl-2-pyridone (50 μL, 0.5 mmol). The crude mixture was purified by flash column chromatography on a silica gel column eluting with EtOAc to provide **8** as a colorless oil (64 mg, 72% yield).
Rf = 0.35 (EtOAc).
^{1}H NMR (400 MHz, CDCl$_3$): δ 7.74 (d, *J* = 6.9 Hz, 1H), 7.50 (d, *J* = 6.9Hz, 1H), 6.23 (t, *J* = 7.0 Hz, 1H), 3.60 (s, 3H) ppm.
^{13}C NMR (101 MHz, CDCl$_3$) δ 159.0, 142.3, 138.9 (q, *J* = 5.1 Hz), 122.8 (q, *J* = 271.5 Hz), 120.6 (q, *J* = 30.7 Hz), 104.1, 38.0 ppm.
^{19}F NMR (376 MHz, CDCl$_3$): δ –66.08 ppm.
HRMS (ESI, *m/z*) calcd. For $C_7H_6F_3NO$ [M+]:177.0401; found 177.0403.
Spectroscopic data are in agreement with previously reported literature data.[3]

***1,3-dimethyl-5-(trifluoromethyl)pyrimidine-2,4(1H,3H)-dione* (9)**

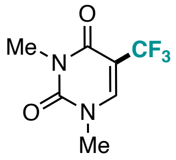

9

Prepared following the general procedure B and starting from methyl uracil (70.1 mg, 0.5 mmol). The crude mixture was purified by flash column chromatography on a silica gel column using a mixture of Cyclohexane/EtOAc (1:1) to provide **9** as a light orange oil (71 mg, 68% yield).

Rf = 0.43 (Cyclohex/EtOAc 1:1 (v/v)).
^{1}H NMR (400 MHz, CDCl$_3$): δ 7.67 (d, *J* = 1.2 Hz, 1H), 3.48 (s, 3H), 3.36 (s, 3H) ppm.

^{13}C NMR (101 MHz, CDCl$_3$) δ 158.8, 151.0, 143.6 (q, *J* = 5.8 Hz), 122.1 (q, *J* = 269.8 Hz), 104.3 (q, *J* = 33.0 Hz), 37.9, 28.2 ppm.

^{19}F NMR (376 MHz, CDCl$_3$): δ –63.83ppm.

HRMS (ESI, *m/z*) calcd. For $C_7H_7F_3N_2O_2$ [M+]: 208.0460; found 208.0455

Spectroscopic data are in agreement with previously reported literature data.[4]

2,4,6-trimethoxy-5-(trifluoromethyl)pyrimidine **(10)**

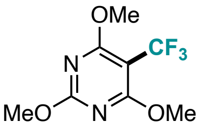

10

Prepared following the general procedure A and starting from 2,4,6-trimethoxypyrimidine (70.1 mg, 0.5 mmol). The crude mixture was purified by flash column chromatography on a silica gel column using a mixture of Cyclohexane/Et_2O (4:1) to provide **10** as a white solid (60 mg, 50% yield).

Rf = 0.57 (Cyclohex/ Et_2O 4:1 (v/v)).

^{1}H NMR (400 MHz, CDCl$_3$): δ 4.02 (s, 6H), 4.00 (s, 3H) ppm.

^{13}C NMR (101 MHz, CDCl$_3$) δ 169.8, 164.9, 123.5 (q, *J* = 271.3 Hz), 89.3 (q, *J* = 34.2 Hz), 55.1, 54.9 ppm.

^{19}F NMR (376 MHz, CDCl$_3$): δ –55.97 ppm.

HRMS (ESI, *m/z*) calcd. For $C_8H_9F_3N_2O_3$ [M+]: 238.0565; found 238.0554

Spectroscopic data are in agreement with previously reported literature data.[4]

2,6-Dimethoxy-3-(trifluoromethyl)pyridine **(11a)** ***and 2,6-dimethoxy-3,5-bis(trifluoromethyl)pyridine*** **(11b)**

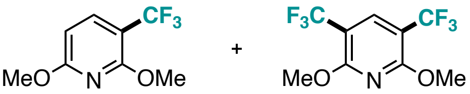

11a
major

11b

Prepared following the general procedure A and starting from 2,6-dimethoxypyridine (65 µL, 0.5 mmol). The crude mixture was purified by flash column chromatography on a silica gel

column using a mixture of Cyclohexane/Et_2O (4:1) to provide **11a** and **11b** as a colourless oil (89.1 mg, 78% yield).

Data for major isomer **11a**:

Rf = 0.36 (Cyclohex/EtOAc 6:1 (v/v)).

^{1}H NMR (400 MHz, $CDCl_3$): δ 7.70 (d, *J* = 8.3Hz, 1H), 6.31 (d, *J* = 8.4Hz, 1H), 4.01 (s, 3H), 3.95 (s, 3H) ppm.

^{13}C NMR (101 MHz, $CDCl_3$): 165.3, 160.6 (q, *J* = 1.9 Hz), 138.93 (q, *J* = 4.5 Hz), 123.9 (q, *J* = 270.1 Hz), 104.5 (q, *J* = 33.5 Hz), 100.9, 54.9, 53.9 ppm.

^{19}F NMR (376 MHz, $CDCl_3$): δ –61.98 ppm.

Spectroscopic data are in agreement with previously reported literature data.[3]

Data for major isomer **11b**:

Rf = 0.43 (Cyclohex/EtOAc 6:1 (v/v)).

^{1}H NMR (400 MHz, $CDCl_3$): δ 7.98 (s, 1H), 4.07 (s, 6H) ppm.

^{13}C NMR (101 MHz, $CDCl_3$): δ 171.3, 122.9 (q, *J* = 271.3 Hz), 104.4 (q, *J* = 34.3 Hz), 101.0, 54.8 ppm.

^{19}F NMR (376 MHz, $CDCl_3$): δ –62.36 ppm.

Spectroscopic data are in agreement with previously reported literature data.[3]

4,6-dimethyl-5-(trifluoromethyl)-2H-pyran-2-one **(12)**

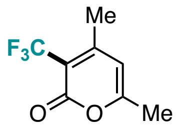

12

Prepared following the general procedure C and starting from *4,6-dimethyl-2H-pyran-2-one* (62.1 mg, 0.5 mmol). The crude mixture was purified by flash column chromatography on a silica gel column, using a mixture of Cyclohexane/Et_2O (6:4) to provide **12** as a white solid (49 mg, 51% yield).

Rf = 0.42 (Cyclohexane/ Et_2O (6:4) (v/v)).

^{1}H NMR (600 MHz, $CDCl_3$): δ 5.92 (s, 1H), 2.33 (q, *J* = 2.9Hz, 3H), 6H), 2.26 (s, 3H) ppm.

^{13}C NMR (151 MHz, CDCl$_3$) δ 164.1, 158.3, 158.2, 123.3 (q, *J* = 274.0 Hz), 111.2 (q, *J* = 31.3Hz), 108.4, 20.8 (q, *J* = 4.0 Hz), 19.9 ppm.
^{19}F NMR (376 MHz, CDCl$_3$): δ –58.06 ppm.
HRMS (ESI, *m/z*) calcd. For $C_{12}H_{13}F_3O_4$ [M+]: 278.0766; found 278.0763.
Spectroscopic data is in agreement with previously reported literature data.[5]

***3-(Trifluoromethyl)-2H-chromen-2-one* (13)**

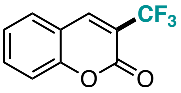

13

Prepared following the general procedure B and starting from coumarin (73.1 mg, 0.5 mmol). The crude mixture was purified by flash column chromatography on a silica gel column using a mixture of Cyclohexane/EtOAc (6:1) to provide **13** as a white solid (35 mg, 32% yield).

Rf = 0.29 (Cyclohex/EtOAc 6:1 (v/v)).
^{1}H NMR (400 MHz, CDCl$_3$): δ 8.16 (s, 1H), 7.71 – 7.66 (m, 1H), 7.62 (d, *J* =7.7, 1.6 Hz, 1H), 7.41 – 7.36 (m, 2H) ppm.
^{13}C NMR (151 MHz, CDCl$_3$) δ 156.0, 154.8, 143.5 (q, *J* = 5.0 Hz), 134.6, 129.6, 125.4, 121.5 (q, *J* = 272.7 Hz), 117.9 (q, *J* = 33.2 Hz), 117.2 ppm.
^{19}F NMR (376 MHz, CDCl$_3$): δ – 66.20 ppm.
HRMS (ESI, *m/z*) calcd. For $C_{10}H_5F_3O_2$ [M+]: 214.0242; found 214.0232.
Spectroscopic data are in agreement with previously reported literature data.[3]

***5,7-dimethoxy-3-(trifluoromethyl)-2H-chromen-2-one* (14)**

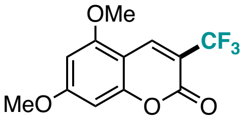

14

Prepared following the general procedure B and starting from 5,7-dimethoxy-2*H*-chromen-2-one (103.1 mg, 0.5 mmol). The crude mixture was purified by flash column chromatography on a silica gel column using a mixture of Cyclohexane/EtOAc (1:1) to provide **14** as a yellow solid (84 mg, 61% yield).

Rf = 0.59 (Cyclohex/EtOAc 1:1 (v/v)).

^{1}H NMR (400 MHz, CDCl$_3$): δ 8.39 (s, 1H), 6.42 (d, *J* = 2.0 Hz, 1H), 6.31 (d, *J* = 2.1 Hz, 1H), 3.92 (s, 3H), 3.88 (s, 3H) ppm.
^{13}C NMR (101 MHz, CDCl$_3$) δ 166.2, 158.3, 157.8, 156.8, 139.0 (q, *J* = 4.9 Hz), 122.2 (q, *J* = 271.2 Hz), 111.7 (q, *J* = 33.2 Hz), 102.4, 95.4, 92.9, 56.3, 56.2 ppm.
^{19}F NMR (376 MHz, CDCl$_3$): δ – 65.36 (d, *J* = 0.9 Hz) ppm.
HRMS (ESI, *m/z*) calcd. For $C_{12}H_9F_3O_4$ [M+]: 274.0453; found 274.0443.
Spectroscopic data are in agreement with previously reported literature data.[1]

5,7-bis(trifluoromethyl)-2,3-dihydrothieno[3,4-b][1,4]dioxine **(15a)** ***and 5-(Trifluoromethyl)-2,3-dihydrothieno[3,4-b][1,4]dioxine*** **(15b)**

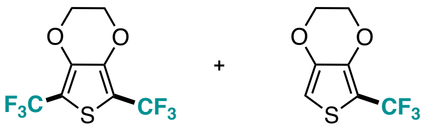

Prepared following the general procedure B and starting from 3,4-ethylenedioxythiophene (55 µL, 0.5 mmol). The crude mixture was purified by flash column chromatography on a silica gel column using a mixture of Cyclohexane/EtOAc (6:1) to provide a mixture of compounds (ratio m:b 1:1.5) as a light-yellow oil (97 mg, 79%).

Data for major isomer **15a**:
Rf = 0.30 (Cyclohex/EtOAc 6:1 (v/v)).
^{1}H NMR (400 MHz, CDCl$_3$): δ 4.36 (s, 4H) ppm.
^{13}C NMR (101 MHz, CDCl$_3$): δ 141.5, 121.6 (q, *J* = 269.4 Hz), 106.7 (q, *J* = 40.5 Hz), 66.8 ppm.
^{19}F NMR (376 MHz, CDCl$_3$): δ –56.07 ppm.
HRMS (ESI, *m/z*) calcd. For $C_8H_4F_6O_2S$ [M+]: 277.9836; found 277.9836.
Spectroscopic data are in agreement with previously reported literature data.[6]

Data for minor isomer **15b**:
Rf = 0.27 (Cyclohex/EtOAc 6:1 (v/v)).
^{1}H NMR (400 MHz, CDCl$_3$): δ 6.49 (s, 1H), 4.32 – 4.29 (m, 2H), 4.25 – 4.22 (m, 2H) ppm.

^{13}C NMR (101 MHz, $CDCl_3$): δ 142.1(q, J = 3.3 Hz), 122.4 (q, J = 267.8 Hz), 104.6 (q, J = 39.2 Hz), 102.3 (q, J = 2.0 Hz), 65.1, 64.4ppm.

^{19}F NMR (376 MHz, $CDCl_3$): δ –55.40 ppm.

HRMS (ESI, *m/z*) calcd. For $C_7H_5F_3O_2S$ [M+]: 209.9962; found 209.9956

Spectroscopic data are in agreement with previously reported literature data.[3]

***Ethyl 4-bromo-5-(trifluoromethyl)-1H-pyrrole-2-carboxylate* (16)**

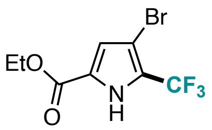

16

Prepared following the general procedure B but with slight modifications (the reaction was performed using a constant voltage of 1.70 V) and starting from ethyl 4-bromo-1*H*-pyrrole-2-carboxylate (109 mg, 0.5 mmol). The crude mixture was purified by flash column chromatography on a silica gel column using a mixture of Cyclohexane/Et_2O (6:1) to provide **16** as a white solid (101 mg, 70% yield).

Rf = 0.25 (Cyclohex/EtOAc 6:1 (v/v)).

^{1}H NMR (400 MHz, $CDCl_3$): δ 10.39 (br s, 1H), 6.93 (s, 1H), 4.38 (q, J = 7.1 Hz, 2H), 1.38 (t, J = 7.1 Hz, 3H) ppm.

^{13}C NMR (101 MHz, $CDCl_3$) δ 160.3, 124.6, 122.5 (q, J = 39.0Hz), 120.1 (q, J = 269.0Hz), 118.4, 98.4 (q, J = 2.8 Hz), 61.9, 14.4 ppm.

^{19}F NMR (376 MHz, $CDCl_3$): δ –60.14ppm.

HRMS (ESI, *m/z*) calcd. For $C_8H_7BrF_3NO_2$ [M+]: 284.9612; found 284.9606.

***Methyl 1-methyl-2-(trifluoromethyl)-1H-indole-3-carboxylate* (17)**

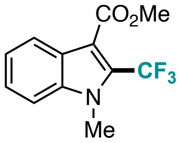

17

Prepared following the general procedure B with slight modifications (the reaction was performed using a constant voltage of 1.70 V) and starting from methyl 1-methyl-1*H*-indole-3-

carboxylate (94.6 mg, 0.5 mmol). The crude mixture was purified by flash column chromatography on a silica gel column using a mixture of Cyclohexane/Et_2O (6:1) to provide **17** as a white solid (72 mg, 56% yield).

Rf = 0.49 (Cyclohex/EtOAc 6:1 (v/v)).

^{1}H NMR (400 MHz, $CDCl_3$): δ 8.10 (d, *J* = 8.2 Hz, 1H), 7.42 – 7.41 (m, 2H), 7.33 – 7.29 (m, 1H), 3.97 (s, 3H), 3.93 (q, *J* = 1.5 Hz, 3H) ppm.

^{13}C NMR (101 MHz, $CDCl_3$) δ 164.0, 137.1, 128.4 (q, *J* = 37.7 Hz), 125.5, 125.4, 122.9, 122.7, 121.0 (q, *J* = 271.1 Hz), 110.3, 109.9 (q, *J* = 2.5 Hz), 52.0, 32.3 (q, *J* = 3.8 Hz) ppm.

^{19}F NMR (376 MHz, $CDCl_3$): δ –55.62 (q, *J* = 1.5 Hz) ppm.

HRMS (ESI, *m/z*) calcd. For $C_{12}H_{10}F_3NO_2$ [M+]: 257.0664; found 257.0663.

Spectroscopic data are in agreement with previously reported literature data.[1]

***3-methyl-2-(trifluoromethyl)-1H-indole* (18)**

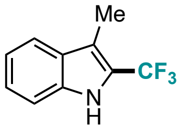

18

Prepared following the general procedure B with slight modifications (the reaction was performed using a constant voltage of 1.70 V) and starting from 3-methylindole (65.6 mg, 0.5 mmol). The crude mixture was purified by flash column chromatography on a silica gel column eluting with a gradient from 10:0 to 9:1 (hexane: EtOAc) to provide **18** as a white solid (70 mg, 70% yield).

Rf = 0.50 (Hex/EtOAc 9:1 (v/v)).

^{1}H NMR (300 MHz, $CDCl_3$) δ 8.16 (s, 1H), 7.65 (d, *J* = 8.0 Hz, 1H), 7.39 (m, 2H), 7.20 (m, 1H), 2.46 (q, *J* = 1.9 Hz, 3H) ppm.

^{13}C NMR (101 MHz, $CDCl_3$) δ 135.3, 128.2, 124.9, 122.3 (q, *J* = 268.4 Hz), 121.7 (q, *J* = 36.9 Hz), 120.5, 120.2, 114.2 (q, *J* = 2.9 Hz), 111.7, 8.5 ppm.

^{19}F NMR (376 MHz, $CDCl_3$): δ –58.65 ppm.

HRMS (ESI, *m/z*) calcd. For $C_{10}H_8F_3N$ [$M^{\bullet+}$]: 199.0604; found 199.0600.

Spectroscopic data are in agreement with previously reported literature data.[3]

1,3,7-trimethyl-8-(trifluoromethyl)-3,7-dihydro-1H-purine-2,6-dione **(19)**

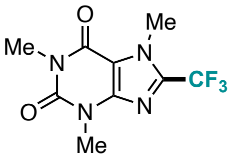

19

Prepared following the general procedure B and starting from caffeine (97.1 mg, 0.5 mmol). The crude mixture was purified by flash column chromatography on a silica gel column, eluting with 6:4 (hexane:EtOAc) mixture, to provide **19** as a white solid (75.5 mg, 58% yield).

Rf = 0.86 (EtOAc).

^{1}H NMR (400 MHz, CDCl$_3$): δ 4.14 (q, J = 1.2 Hz, 1H), 3.57 (s, 1H), 3.39 (s, 1H) ppm.

^{13}C NMR (101 MHz, CDCl$_3$) δ 155.6, 151.5, 146.6, 139.1 (q, J = 40.1 Hz), 118.3 (q, J = 271.3 Hz), 109.8, 33.3 (q, J = 2.1 Hz), 30.0, 28.2 ppm.

^{19}F NMR (376 MHz, CDCl$_3$): δ – 62.46 ppm.

HRMS (ESI, *m/z*) calcd. For $C_9H_9F_3N_4O_2$ [$M^{\bullet+}$]: 262.0672; found 262.0670.

Spectroscopic data are in agreement with previously reported literature data.[3]

7-((1,3-dioxolan-2-yl)methyl)-1,3-dimethyl-8-(trifluoromethyl)-3,7-dihydro-1H-purine-2,6-dione **(20)**

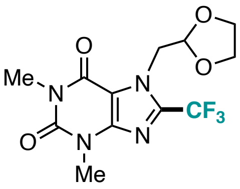

20

Prepared following the general procedure B and starting from Doxofylline (133.2 mg, 0.5 mmol). The crude mixture was purified by flash column chromatography on basic alumina column using a mixture of Cyclohexane/EtOAc (2:1) to provide **20** as a white solid (89 mg, 53% yield).

Rf = 0.67 (Hex/EtOAc 2:1 (v/v)).

^{1}H NMR (400 MHz, CDCl$_3$): δ 5.33 (t, J = 4.3Hz, 1H), 4.67 (d, J = 4.4 Hz, 2H), 3.96 – 3.88 (m, 4H), 3.59 (s, 3H), 3.42 (s, 3H) ppm.

^{13}C NMR (101 MHz, CDCl$_3$) δ 155.4, 151.4, 146.8, 139.3 (q, J = 40.0 Hz), 118.4 (q, J = 271.5 Hz), 109.6, 101.0, 65.5, 48.8 (q, J = 1.8 Hz), 30.1, 28.4 ppm.

^{19}F NMR (376 MHz, CDCl$_3$): δ – 60.81 ppm.

Spectroscopic data are in agreement with previously reported literature data.[7]

1-((2R,4S,5R)-4-Hydroxy-5-(hydroxymethyl)tetrahydrofuran-2-yl)-5 (trifluoromethyl)pyrimidine-2,4(1H,3H)-dione(Trifluridine) **(21)**

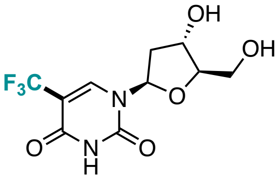

21

Starting from deoxyuridine (68.5 mg, 0.3 mmol) and following the general procedure C to provide **21** as a light-yellow solid (54 mg, 61 % yield).

Rf = 0.67 (AcOEt).

^{1}H NMR (400 MHz, DMSO-d_6): δ 11.83 (s, 1H), 8.73 (s, 1H), 6.08 (t, J = 6.1 Hz, 1H), 5.29 (d, J = 4.4 Hz, 1H), 5.23 (t, J = 4.5 Hz, 1H), 4.27 – 4.22 (m, 1H), 3.83 (q, J = 3.0 Hz, 1H), 3.68 – 3.56 (m, 2H), 2.20 (t, J = 5.7 Hz, 2H) ppm.

^{13}C NMR (151 MHz, DMSO-d_6) δ 159.1, 149.6, 142.3 (q, J = 6.6 Hz), 122.8 (q, J = 269.2 Hz), 102.7 (q, J = 31.9 Hz), 87.7, 85.5, 69.4, 60.3, 40.7 ppm.

^{19}F NMR (376 MHz, DMSO-d$_6$): δ – 61.59 ppm.

HRMS (ESI, *m*/*z*) calcd. For $C_{10}H_{10}F_3N_2O_5$ [M–H]$^-$: 295.0547; found 295.0552.

Spectroscopic data are in agreement with previously reported literature data.[3]

5-((3,5-dimethyl-2-(trifluoromethyl)phenoxy)methyl)oxazolidin-2-one (22a) and 5-((3,5-Dimethyl-2,4-bis(trifluoromethyl)phenoxy)methyl)oxazolidin-2-one **(22b)**

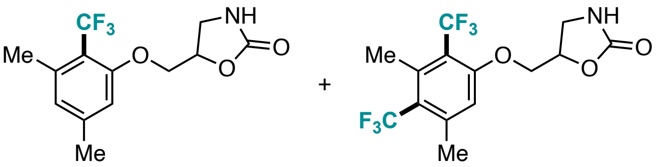

22a
major

22b

Prepared following the general procedure C and starting from metaxalone (110.7 mg, 0.5 mmol). The crude mixture was purified by flash column chromatography on a silica gel column

using a mixture of Cyclohexane/EtOAc (4:1) to provide a mixture of compounds (ratio m:b 62:38) as a light-yellow oil (97 mg, 62%).

Data for major isomer **22a**:

Rf = 0.51 (Cyclohex/EtOAc 4:1 (v/v)).

^{1}H NMR (400 MHz, CDCl$_3$): δ 6.61 (s, 2H), 5.38 (brs, 1H), 5.02 – 4.94 (m, 1H), 4.15 (d, J = 4.8Hz, 2H), 3.82 – 3.76 (m, 1H), 3.63 – 3.59 (m, 1H), 2.46 – 2.43 (m, 6H) ppm.

^{13}C NMR (101 MHz, CDCl$_3$) δ 159.1, 139.8, 74.1, 67.8, 42.6, 21.2, 14.3 ppm. Some of the quaternary carbon signals could not be observed.

^{19}F NMR (376 MHz, CDCl$_3$): δ –53.13 - –53.16 (m, 3F) ppm.

Data for minor isomer **22b**:

Rf = 0.62 (Cyclohex/EtOAc 4:1 (v/v)).

^{1}H NMR (400 MHz, CDCl$_3$): δ 6.69 (s, 2H), 5.34 (brs, 1H), 5.02 – 4.94 (m, 1H), 4.24 – 4.22 (m, 2H), 3.82 – 3.76 (m, 1H), 3.69 – 3.65 (m, 1H), 2.53 – 2.49 (m, 6H) ppm.

^{13}C NMR (101 MHz, CDCl$_3$) δ 157.7, 143.0, 114.9, 73.6, 68.8, 42.5, 21.8, 17.5 ppm. Some of the quaternary carbon signals could not be observed.

^{19}F NMR (376 MHz, CDCl$_3$): δ –53.04 - –53.07 (m, 3F), –53.35 - –53.38 (m, 3F) ppm.

Spectroscopic data is in agreement with previously reported literature data.[3]

N-(2-(5-methoxy-2-(trifluoromethyl)-1H-indol-3-yl)ethyl)acetamide (23a)

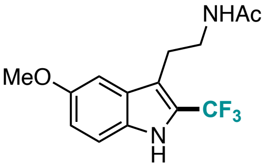

23a

Prepared following the general procedure C and starting from melatonin (116 mg, 0.5 mmol). The crude mixture was purified by flash column chromatography on a silica gel column using a mixture of Cyclohexane/EtOAc (1:1) to provide **23a** as a white solid (51 mg, 34% yield).

Rf = 0.47 (Cyclohex/EtOAc 1:1 (v/v)).

^{1}H NMR (400 MHz, CDCl$_3$): δ 8.92 (s, 1H), 7.30 (d, J = 8.9 Hz, 1H), 7.08 (d, J = 2.4 Hz, 1H), 6.97 (dd, J = 8.9, 2.4 Hz, 1H), 5.67 – 5.66 (m, 1H), 3.84 (s, 3H), 3.55 (q, J = 6.5Hz, 2H), 3.11 – 3.06 (m, 2H), 1,92 (s, 3H) ppm.

^{13}C NMR (101 MHz, CDCl$_3$) δ 170.6, 154.9, 130.7, 127.9, 122.1 (q, *J* = 268.7Hz), 122.8 (q, *J* = 36.7 Hz), 116.2, 114.6 (q, *J* = 2.8Hz), 113.1, 100.7, 55.9, 40.1, 24.1, 23.4 ppm.
^{19}F NMR (376 MHz, CDCl$_3$): δ – 57.92 ppm.
Spectroscopic data are in agreement with previously reported literature data.[8]

***N-(2-(5-methoxy-2,4-bis(trifluoromethyl)-1H-indol-3-yl)ethyl)acetamide* (23b) *and N-(2-(5-methoxy-2,6-bis(trifluoromethyl)-1H-indol-3-yl)ethyl)acetamide* (23c)**

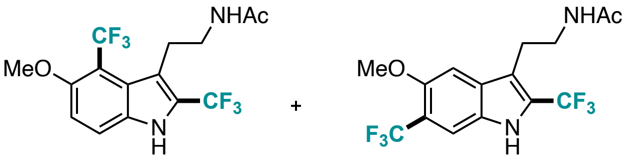

23b
major

23c

Starting from melatonin (116.2 mg, 0.5 mmol) and following the general procedure C to provide a mixture of 2 isomers (ratio 72:28) as a yellow oil (84 mg, 46% yield).

Data for the major isomer **23b**:
Rf = 0.57 (Cyclohex/EtOAc 1:1 (v/v)).
^{1}H NMR (400 MHz, CDCl$_3$): δ 9.64 (br s, 1H), 7.53(d, *J* = 9.0 Hz, 1H), 7.08 (d, *J* = 9.0 Hz, 1H), 5.63 – 5.62 (m, 1H), 3.91 (s, 3H), 3.47 (q, *J* = 6.8 Hz, 2H), 3.12 – 3.08 (m, 2H), 1.95 (s, 3H) ppm.
^{13}C NMR (75 MHz, CDCl$_3$) δ 171.0, 154.1, 132.5, 126.5 (q, *J* = 35.7 Hz), 125.0 (q, *J* = 272.4 Hz), 123.0, 121.9 (q, *J* = 269.9 Hz), 117.8, 113.3, 110.2 (q, *J* = 32.1 Hz), 60.6, 40.5, 25.3 (q, *J* = 5.0 Hz), 23.0 ppm.
^{19}F NMR (376 MHz, CDCl$_3$): δ –52.83, –58.11 ppm.
Spectroscopic data are in agreement with previously reported literature data.[3]

SCALE-UP REACTIONS.

<u>General procedure for scale-up reactions</u>

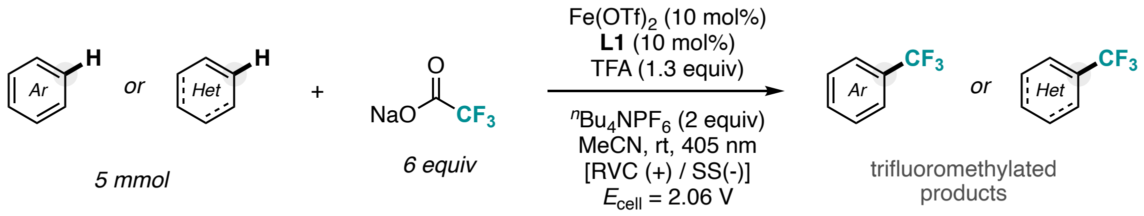

An oven-dried 100 mL Schlenk tube was equipped with a stirring bar and charged with the corresponding (hetero)arene (5 mmol, 1 equiv), NaO_2CCF_3 (4.1 g, 30 mmol, 6 equiv), $Fe(OTf)_2$ (178 mg, 0.5 mmol, 10 mol%), 4,4'-dimethoxy-2,2'-bipyridine **L1** (106 mg, 0.5 mmol, 10 mol%) and $TBAPF_6$ (3.87 g, 10 mmol, 2 equiv.). All reagents were added in open-air conditions. The reticulated vitreous carbon (RVC) anode (50x25x3mm) and stainless steel (SS) cathode (50x25x1.7mm) were then inserted, and the vial was closed with a perforated septum [*note*: the RVC electrode was supported over a stainless steel spike of a total length of 180 mm]. The Schlenk tube was evacuated and back-filled with argon (x3 times). Against a positive argon flow, degassed dry MeCN (50 mL) was added *via* a syringe. The reaction was stirred (650 rpm) under constant potential electrolysis (CPE, E_{cell}= 2.06 V), while irradiated with a 405 nm light (EvoluChem LEDs from HepatoChem®), at a distance of 3 cm for 70 h [*note:* a mirror was placed behind the Schlenk tube at a distance of 3 cm, and two fans were used for ventilation]. Once this time elapsed, the electrodes were rinsed with ethyl acetate (10 mL), and the crude was transferred to a 250 mL separatory funnel. The organic phase was washed with a saturated solution of $NaHCO_3$ (30 mL). After phase separation, the aqueous layer was extracted twice with EtOAc (20 mL, each). The combined organic layers were dried over anhydrous Na_2SO_4, then evaporated under reduced pressure. The crude product was purified by automated flash column chromatography on SiO_2 gel.

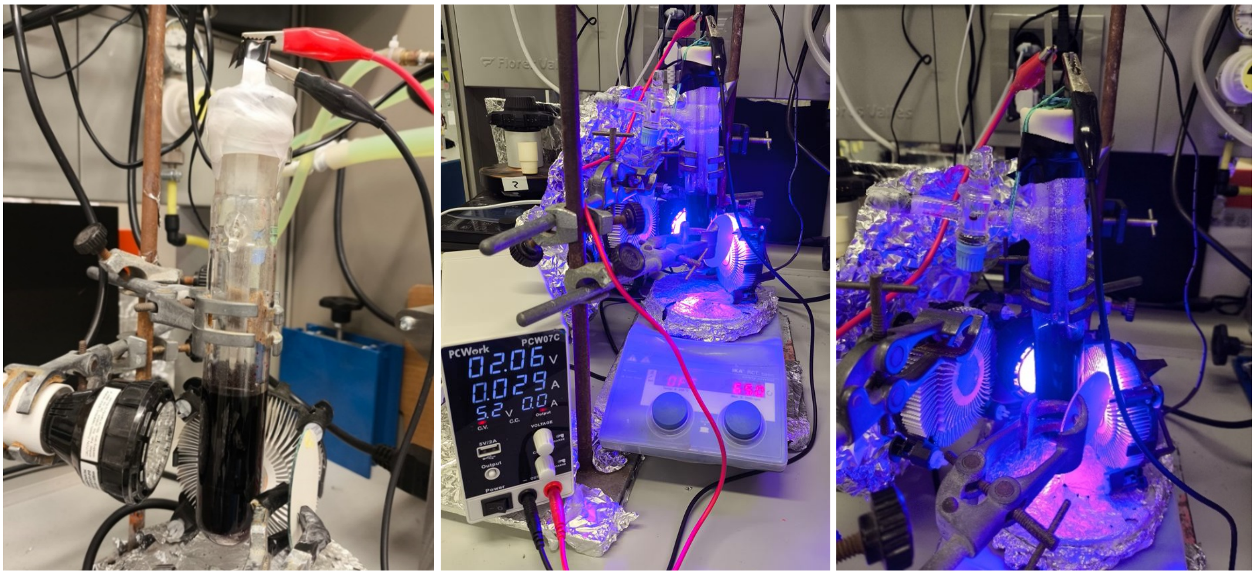

Fig. S3. Gram scale reaction set-up.

1,3,5-trimethoxy-2-(trifluoromethyl)benzene

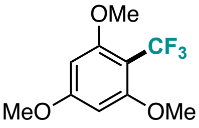

2

Prepared following the scale-up general procedure, using 1,3,5-trimethoxybenzene (841 mg, 5 mmol). Reaction time: 70 h. The crude mixture was purified by automated flash column chromatography on a silica gel column, eluting with a gradient from 10:0 to 6:4 (hexane: EtOAc), to provide **2** as a white solid (520 mg, 44% yield).

1,3,7-Trimethyl-8-(trifluoromethyl)-3,7-dihydro-1H-purine-2,6-dione

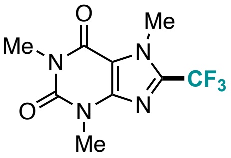

19

Prepared following the scale-up general procedure, using caffeine (971 mg, 5 mmol). Reaction time: 110 h. The crude mixture was purified by automated flash column chromatography on a silica gel column, eluting with 6:4 (hexane:EtOAc) to provide **19** as a white solid (755 mg, 58% yield)

UNSUCCESSFUL AND LOW-YIELDING SUBSTRATES

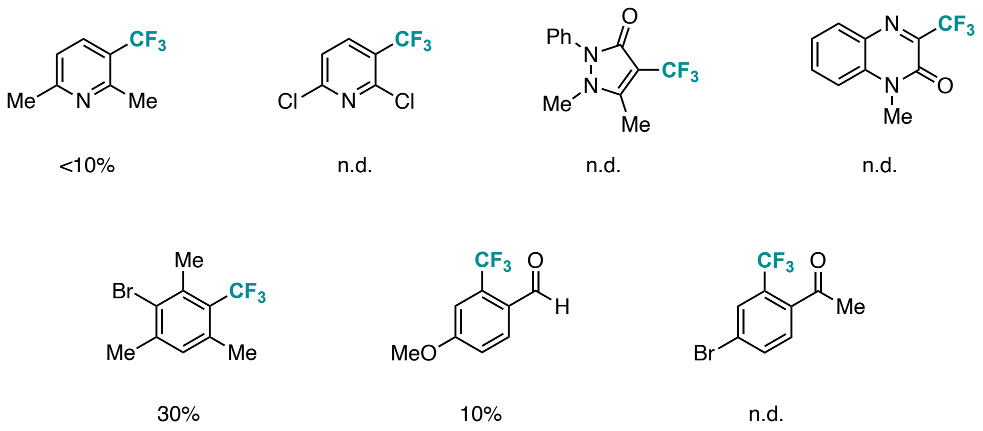

Fig. S4. Low-yielding substrates.

Yields were determined by ^{19}F NMR using hexafluorobenzene as internal standard.

UV-VIS ABSORPTION MEASUREMENTS

Samples for UV-Vis monitoring were prepared at the concentration of the optimized reaction conditions and then diluted for UV-Vis spectra acquisition, resulting in a 48 μM concentration of iron. Measurements were recorded in a Horiba Duetta fluorescence and absorbance spectrometer.

UV-Vis spectrum of Fe(II) + sodium trifluoroacetate

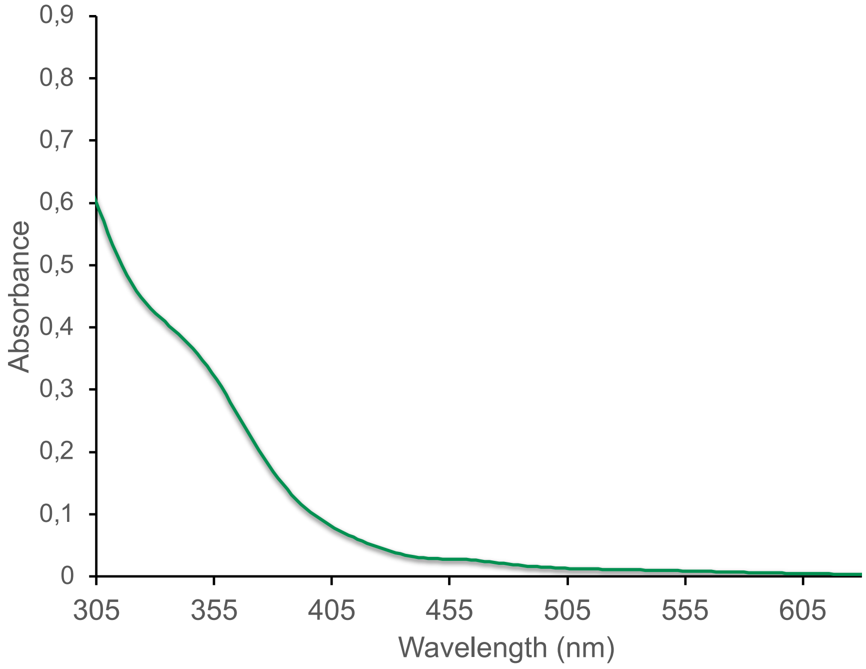

Fig. S5. UV-Vis spectrum of Fe(II) trifluoroacetate species. Conditions: $Fe(OTf)_2$ (10.6 mg, 0.03 mmol, 1 equiv.) and NaO_2CCF_3 (163.2 mg, 1.2 mmol, 60 equiv) in 3 mL MeCN. The solution was diluted before recording UV- Vis spectrum.

UV-Vis spectrum of isolated [Fe(L1)$_3$](PF$_6$)$_2$

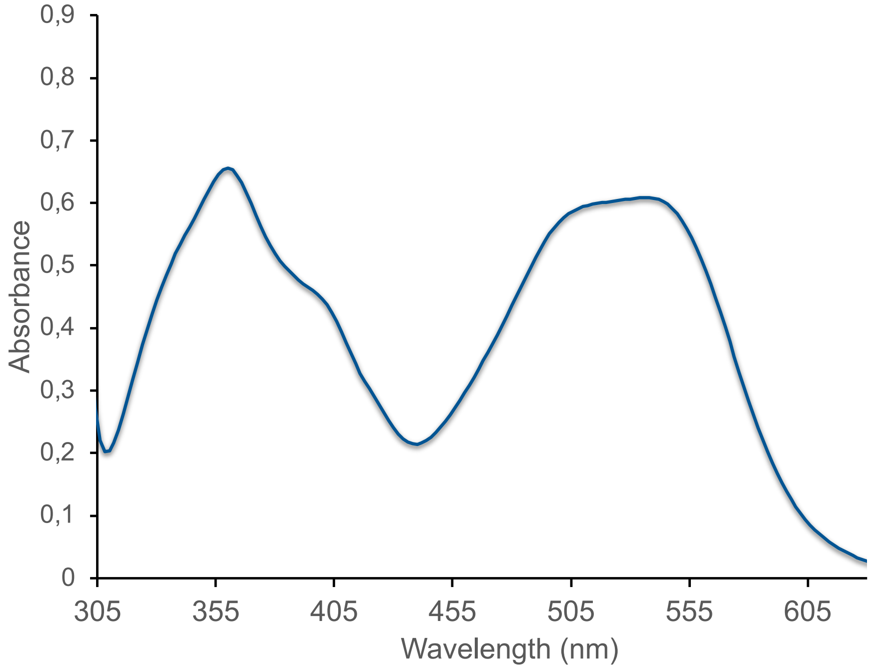

Fig. S6. UV-Vis spectrum of [Fe(L1)$_3$](PF$_6$)$_2$. Conditions: [Fe(**L1**)$_3$](PF$_6$)$_2$ (33 mg, 0.03 mmol) in 3 mL MeCN. The solution was diluted before recording UV- Vis spectrum.

UV-Vis spectrum of a mixture of $Fe(OTf)_2$, L1 and sodium trifluoroacetate in the absence of trifluoroacetic acid.

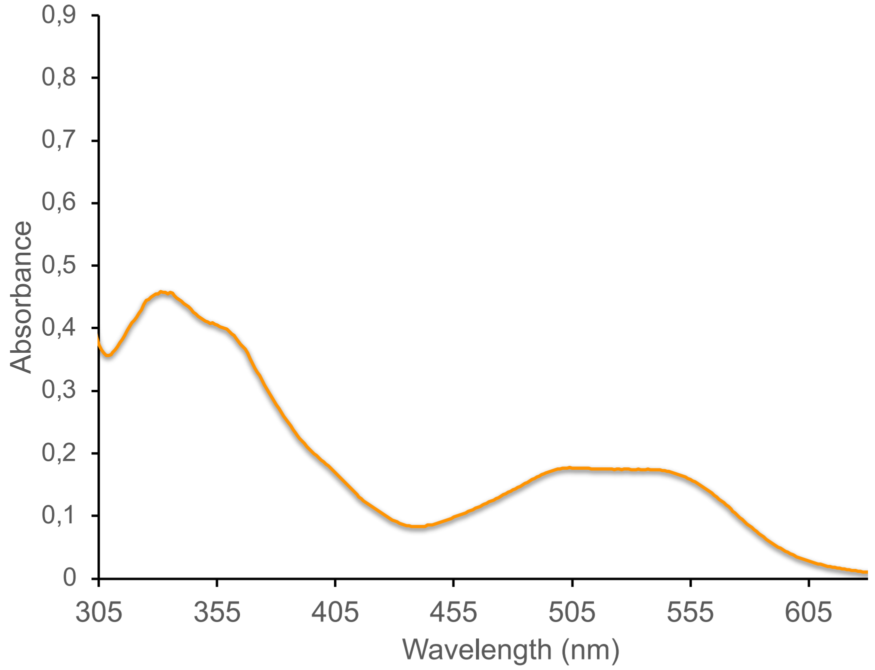

Fig. S7. UV-Vis spectrum of a mixture of $Fe(OTf)_2$, L1 and sodium trifluoroacetate in the absence of trifluoroacetic acid. Conditions: $Fe(OTf)_2$ (10.6 mg, 0.03 mmol, 1 equiv.), 4,4'-dimethoxy-2,2'-bipyridine (**L1**) (6.5 mg, 0.03 mmol, 1 equiv) and NaO_2CCF_3 (163.2 mg, 1.2 mmol, 60 equiv) in 3 mL MeCN. The solution was diluted before recording UV- Vis spectrum.

UV-Vis spectrum of a mixture of Fe(OTf)$_2$, L1 and sodium trifluoroacetate in the presence of trifluoroacetic acid.

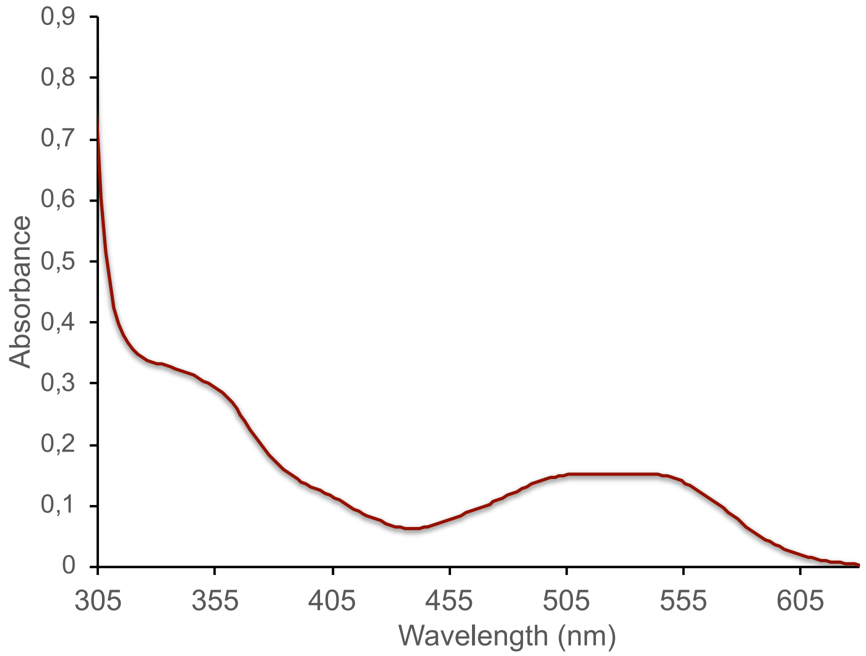

Fig. S8. UV-Vis spectrum of a mixture of Fe(OTf)$_2$, L1 and sodium trifluoroacetate in the presence of trifluoroacetic acid. Conditions: Fe(OTf)$_2$ (10.6 mg, 0.03 mmol, 1 equiv.), 4,4'-dimethoxy-2,2'-bipyridine (**L1**) (6.5 mg, 0.03 mmol, 1 equiv), NaO_2CCF_3 (163.2 mg, 1.2 mmol, 60 equiv) and trifluoroacetic acid (30µL, 0.4 mmol, 13 equiv) in 3 mL MeCN. The solution was diluted before recording UV- Vis spectrum.

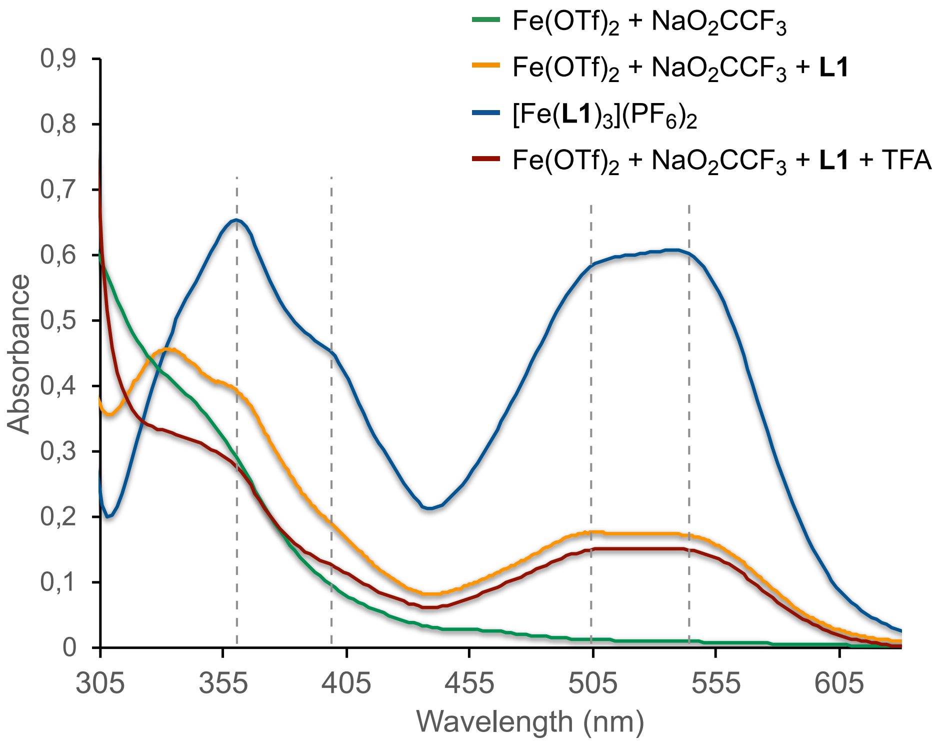

Fig. S9. Overlap of UV-Vis spectra of a mixture of Fe(II)/L1/NaO_2CCF_3 without TFA (orange trace), a mixture of Fe(II)/L1/NaO_2CCF_3 with TFA (red trace), pure $[Fe(L1)_3](PF_6)_2$ (blue trace) and iron(II) trifluoroacetate species (green trace).

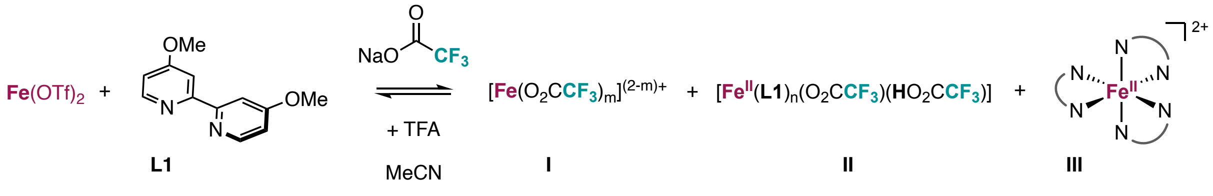

According to the data, under reaction conditions, a mixture of iron(II) triflate, **L1**, sodium trifluoroacetate and TFA (ration 1:1:60:13) results in the formation of non-ligated Fe trifluoroacetate species (**I**), ligated Fe species containing both **L1** and trifluoroacetate ligands (**II**) and homoleptic trisbipyridine-type Fe complex (**III**).

Quantification of $[Fe(L1)_3]^{2+}$ species under reaction conditions.

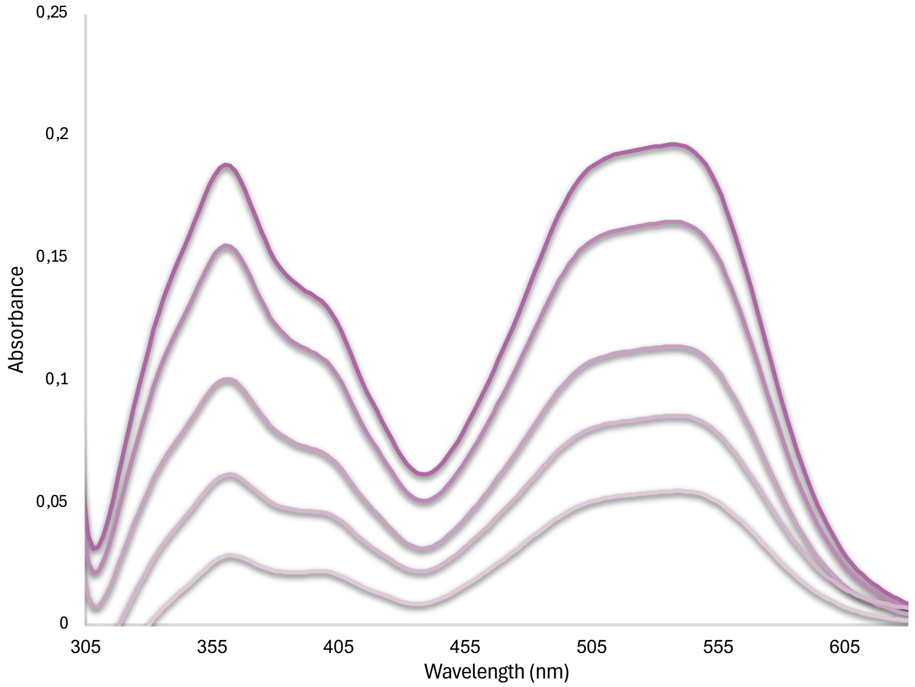

Fig. S10. UV-Vis spectra of $[Fe(L1)_3](PF_6)_2$ in MeCN at different concentrations (4.00 μM, 5.95 μM, 8.41 μM, 11.16 μM, 14.35 μM).

$[Fe(L1)_3](PF_6)_2$ (μM)	Absorbance (542 nm)
4.00	0,0548
5.95	0,0854
8.41	0,1136
11.16	0,1642
14.35	0,1956

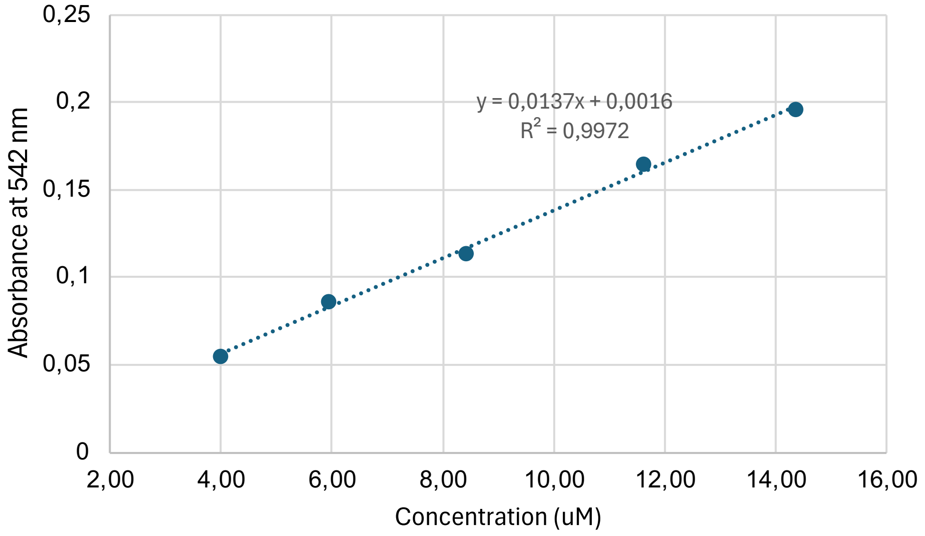

Absorbance (542nm) = 0.0137 $[[Fe(\mathbf{L1})_3]^{2+}]$ + 0.0016

Under reaction conditions: Absorbance (542 nm) = 0.1514.

$$0.1514 = 0.0137\,[[Fe(\mathbf{L1})_3]^{2+}] + 0.0016$$

$$[[Fe(\mathbf{L1})_3]^{2+}] = 10.93\ \mu M$$

Overall concentration of iron in solution = 48 μM

Concentration of $[Fe(L1)_3]^{2+}$ = 10.9 μM, accounting for 23% of total iron content.

Considering the presence of other ligated Fe species in solution, that could absorb in the same region, it can be stated that the maximum expected concentration of trisbipyridine-type Fe species would be 23%.

CYCLIC VOLTAMMETRY MEASUREMENTS

All the cyclic voltammograms were recorded at room temperature with a scan rate of 0.1 V/s. A typical three-electrode cell was employed, which consisted of a glassy carbon (GC) working electrode (3 mm diameter), a platinum wire as the counter electrode, and an Ag/AgCl (0.1 M KCl) reference electrode. All solutions were prepared in acetonitrile containing 0.1 M tetrabutylammonium hexafluorophosphate ($TBAPF_6$) as the supporting electrolyte. The glass electrochemical cell was kept closed with a stopper during the measurements. Oxygen was removed from the solvent by purging with argon before each measurement. The potential of ferrocenium/ferrocene (Fc^+/Fc) couple was used as an internal reference system ($E_{1/2}$ (Fc^+/Fc) = +0.38 V vs SCE; $E_{1/2}$ (Fc^+/Fc) = +0.33 V *vs* Ag/AgCl (0.1 M KCl)).[9,10]

Representative reaction mixture

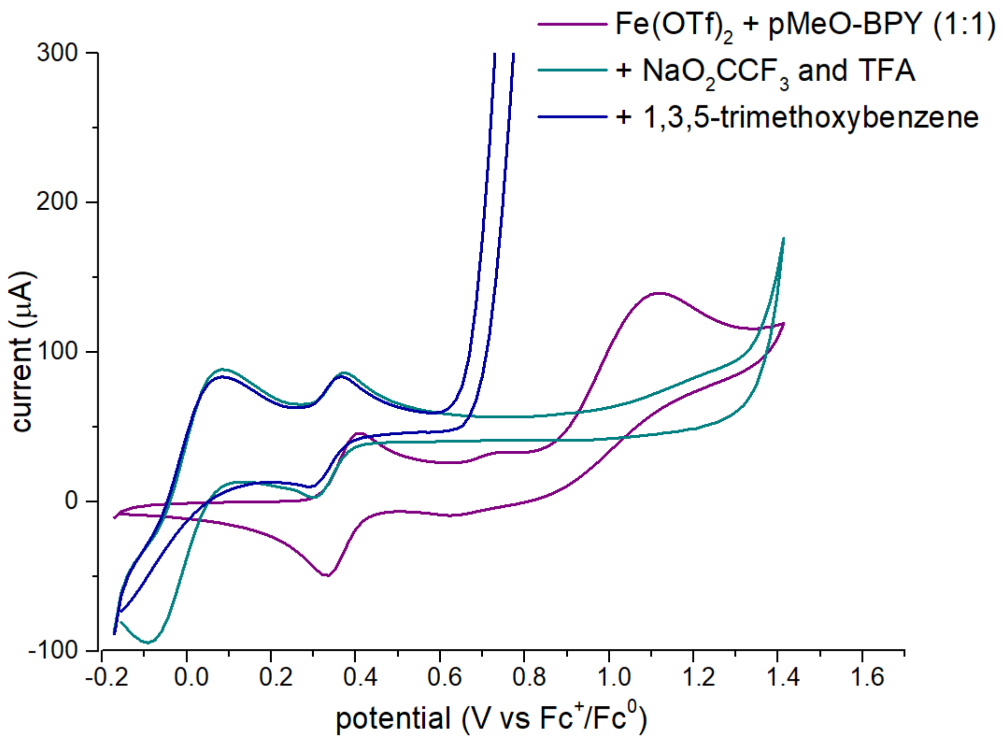

Fig. S11. Cyclic voltammograms of: **(i)** $Fe(OTf)_2$ (10 mM) and **L1** (10 mM) (purple trace); **(ii)** sequential addition of NaO_2CCF_3 (600 mM) and trifluoroacetic acid (TFA, 130 mM) to solution (i) (green trace); **(iii)** sequential addition of 1,3,5-trimethoxybenzene (100 mM) to solution (ii) (dark blue trace).

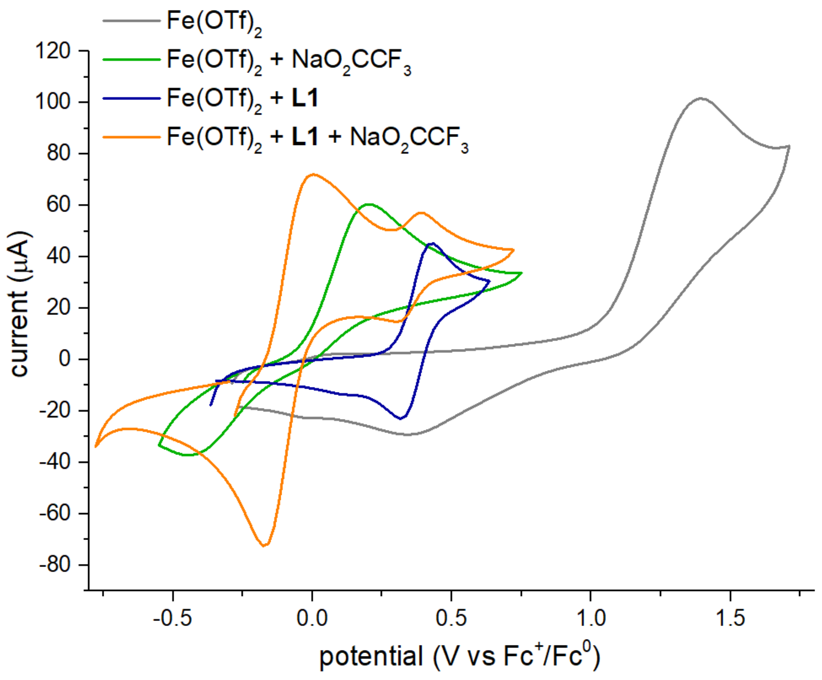

Fig. S12. Cyclic voltammograms of: **(i)** $Fe(OTf)_2$ (10 mM) [grey trace]; **(ii)** $Fe(OTf)_2$ (10 mM) and NaO_2CCF_3 (600 mM) [green trace], **(iii)** $Fe(OTf)_2$ (10 mM) and **L1** (10 mM) [blue trace], **(iv)** $Fe(OTf)_2$ (10 mM), **L1** (10 mM) and NaO_2CCF_3 (600 mM) [orange trace]. Every independent measurement was done in the presence of trifluoroacetic acid (TFA, 130 mM).

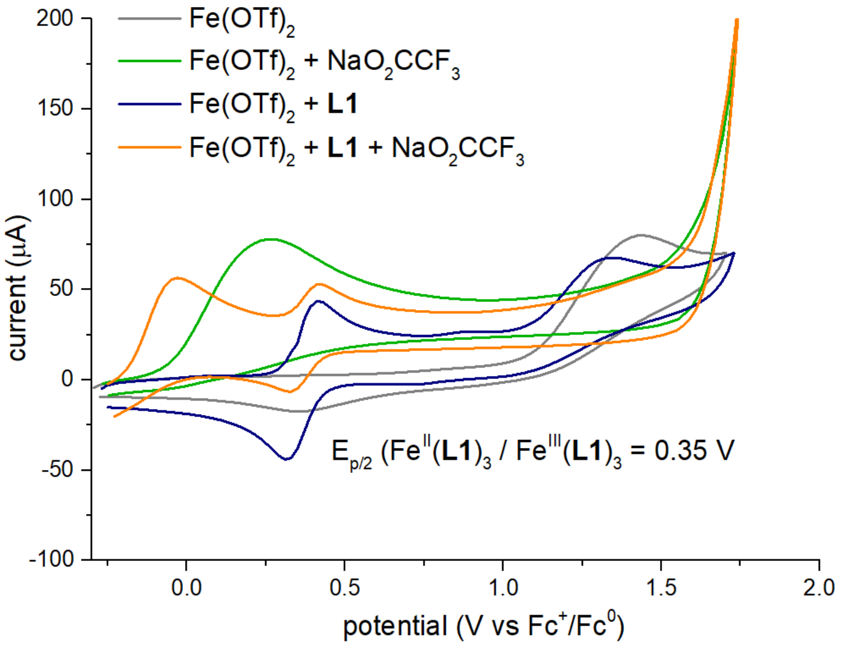

Fig. S13. Cyclic voltammograms of: **(i)** $Fe(OTf)_2$ (10 mM) [grey trace]; **(ii)** $Fe(OTf)_2$ (10 mM) and NaO_2CCF_3 (600 mM) [green trace], **(iii)** $Fe(OTf)_2$ (10 mM) and **L1** (10 mM) [blue trace], **(iv)** $Fe(OTf)_2$ (10 mM), **L1** (10 mM) and NaO_2CCF_3 (600 mM) [orange trace]. **Note:** In contrast to Fig. S12, the independent measurements were done in the absence of TFA.

Effect of acid addition

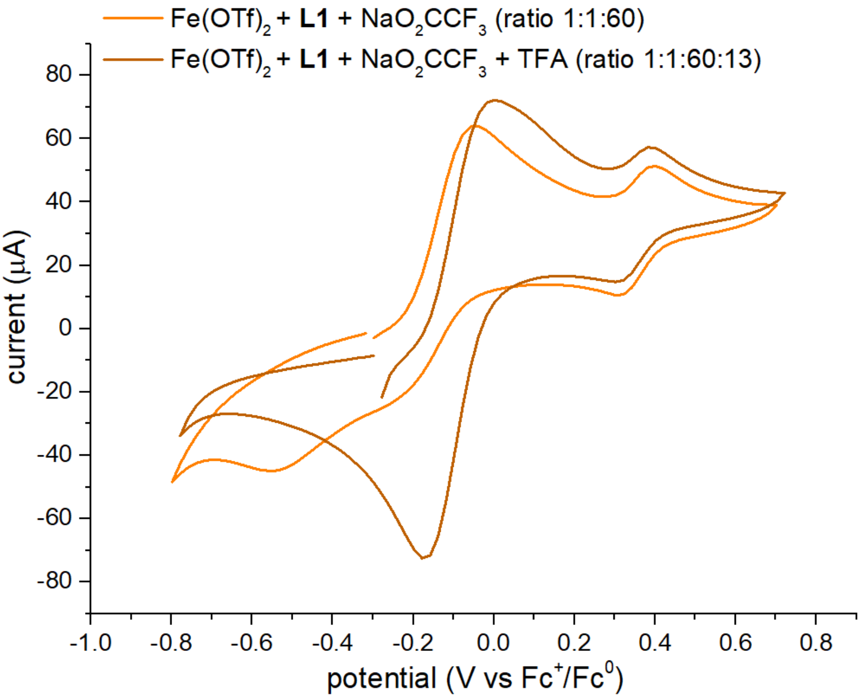

Fig S14. Cyclic voltammograms of: **(i)** $Fe(OTf)_2$ (10 mM), **L1** (10 mM) and NaO_2CCF_3 (600 mM) [light orange trace]; **(ii)** $Fe(OTf)_2$ (10 mM), **L1** (10 mM), NaO_2CCF_3 (600 mM) and TFA (130 mM) [brown trace].

<u>Comments</u>: A new reversible redox pair with $E_{p/2} = -0.1$ V (vs $Fc^{+/0}$) is observed when adding TFA to the reaction mixture. The new redox pair must contain trifluoroacetates and **L1** in its coordination sphere, since this behavior is not observed in the absence of ligand and in the presence of TFA (refer to green line, Fig. S13). The presence of acid presumably stabilizes the oxidized form (Fe^{III}) of a [Fe(**L1**)$_x$($OCCF_3$)$_y$] complex, leading to reversibility.

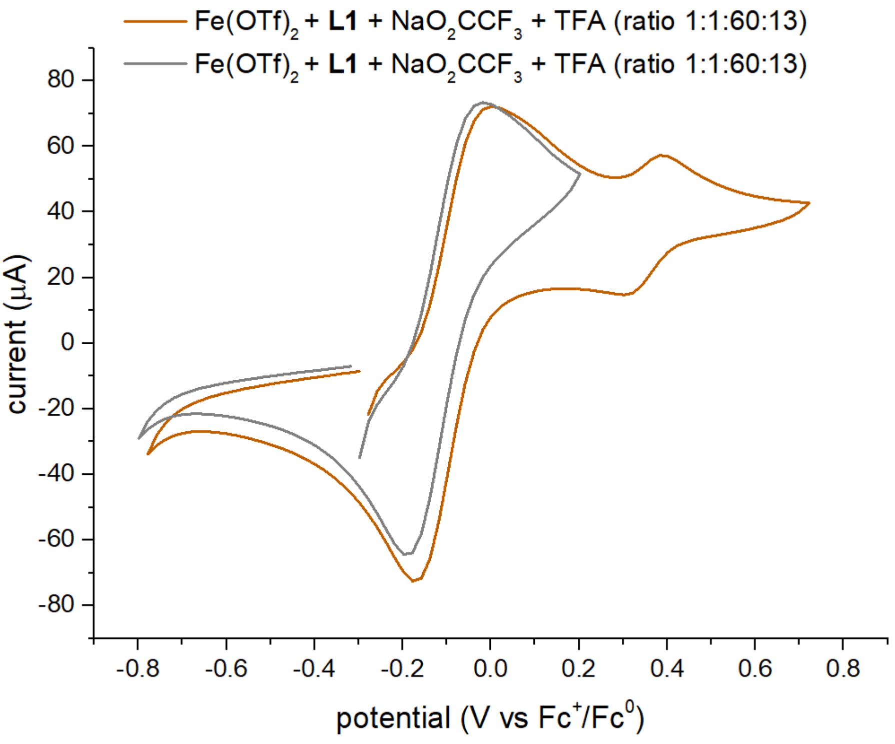

Fig S15. Cyclic voltammograms of $Fe(OTf)_2$ (10 mM), **L1** (10 mM), NaO_2CCF_3 (600 mM) and TFA (130 mM), acquired with two different scan windows: **(i)** E-begin/E-end=- 0.3 V; E-vertex 1= +0.7 V; E-vertex 2= -0.8 V [brown trace]; **(ii)** E-begin/E-end=- 0.3 V; E-vertex 1= +0.2 V; E-vertex 2= -0.8 V [grey trace].

Comments: The new reversible redox pair ($E_{p/2}$ = −0.10 V, vs $Fc^{+/0}$) is formed independently of the oxidation of the Fe(**LI**)$_3$ complex ($E_{p/2}$ = + 0.36 V, vs $Fc^{+/0}$). This is corroborated when cutting the scan window to avoid Fe(**L1**)$_3$ oxidation (grey trace of Fig S15).

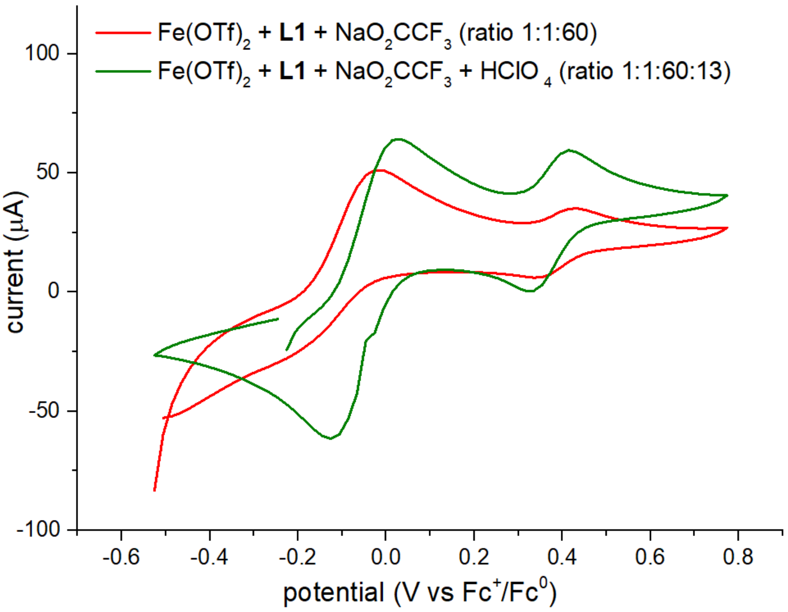

Fig S16. Cyclic voltammograms of: **(i)** $Fe(OTf)_2$ (10 mM), **L1** (10 mM) and NaO_2CCF_3 (600 mM) [red trace]; and **(ii)** $Fe(OTf)_2$ (10 mM), **L1** (10 mM), NaO_2CCF_3 (600 mM) and perchloric acid (130 mM) [green trace].

***Comments*:** The new reversible redox pair ($E_{p/2}$ = −0.10 V, vs $Fc^{+/0}$) is also formed in the presence of a strong inorganic acid, such as perchloric acid ($HClO_4$). In this case, TFA can be formed *in situ* when mixing $HClO_4$ with NaO_2CCF_3.

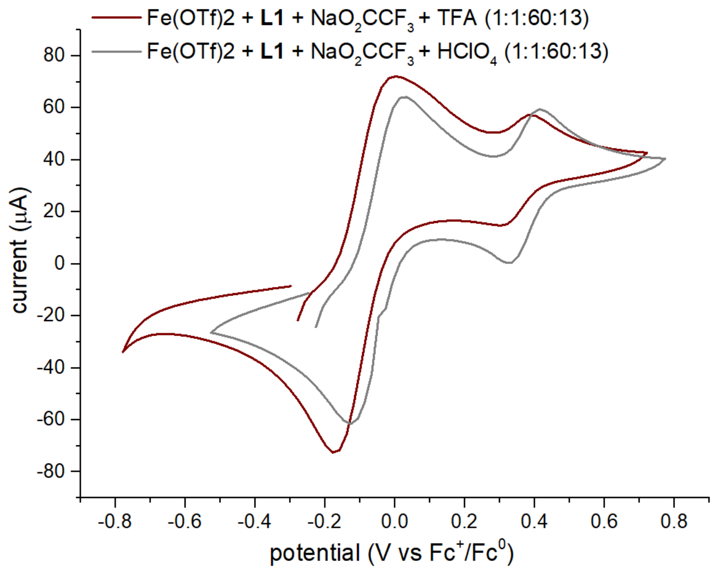

Fig S17. Cyclic voltammograms of: **(i)** $Fe(OTf)_2$ (10 mM), **L1** (10 mM) and NaO_2CCF_3 (600 mM) + TFA (130 mM) [brown trace]; and **(ii)** $Fe(OTf)_2$ (10 mM), **L1** (10 mM), NaO_2CCF_3 (600 mM) and perchloric acid (130 mM) [grey trace].

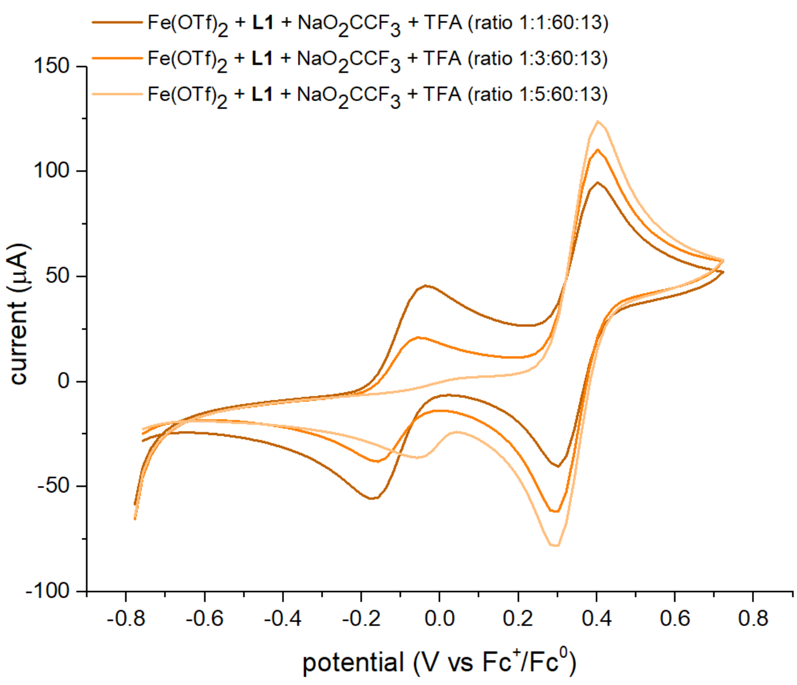

Fig S18. Cyclic voltammograms of **(i)** $Fe(OTf)_2$ (10 mM), **L1** (10 mM) and NaO_2CCF_3 (600 mM) + TFA (130 mM) [brown trace]; and sequential additions of **L1**: **(ii) L1** (30 mM) → ratio Fe:**L1** = 1:3; **(ii) L1** (50 mM) → ratio Fe:**L1** 1:5.

Comments: With increasing amounts of **L1**, the first reversible redox pair ($E_{p/2}$ = − 0.1 V, vs $Fc^{+/0}$) disappears. Specifically, with a 1:5 ratio of Fe:**L1**, only the Fe(**L1**)$_3$ complex is observed together with a new reduction curve (Ep = −0.05 V, vs $Fc^{+/0}$), indicating the presence of a Fe(III) species.

Photochemical and redox stability

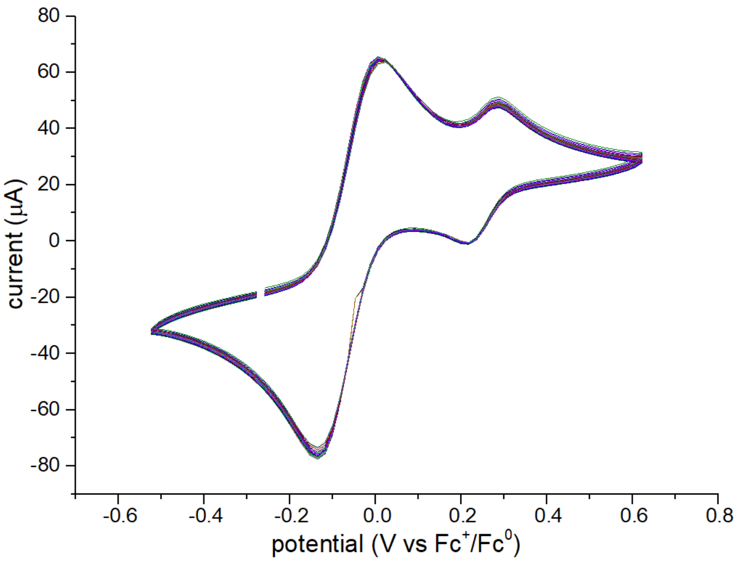

Fig S19. Sequential cyclic voltammograms of $Fe(OTf)_2$ (10 mM) + **L1** (10 mM) + NaO_2CCF_3 (600 mM) + TFA (130 mM); in the dark. 55 total scans.

<u>Comments</u>: Both redox pairs are stable after 55 total scans, under dark conditions.

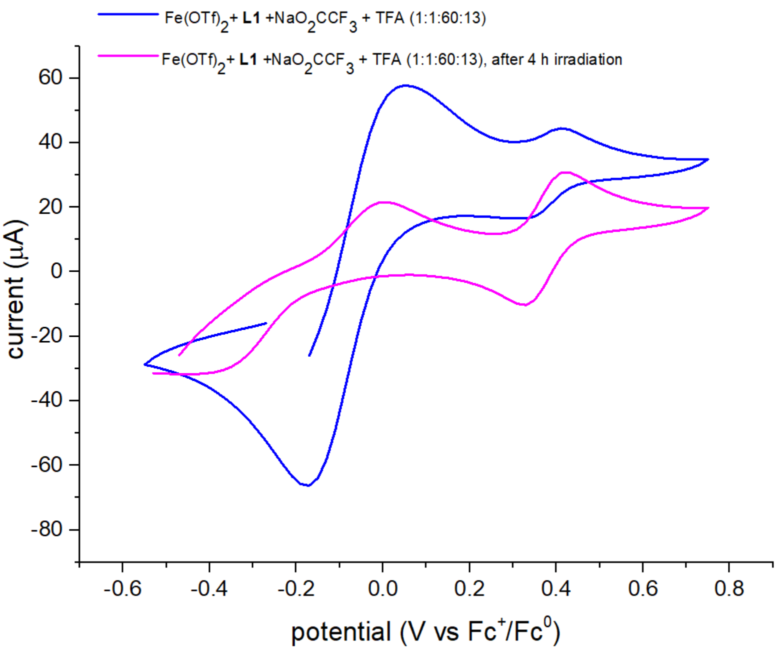

Fig S20. Cyclic voltammograms of: **(i)** $Fe(OTf)_2$ (10 mM), **L1** (10 mM) and NaO_2CCF_3 (600 mM) and TFA (130 mM) [blue trace]; and **(ii)** $Fe(OTf)_2$ (10 mM), **L1** (10 mM), NaO_2CCF_3 (600 mM) and TFA (130 mM), after 4 hours of irradiation at 405 nm [pink trace].

Comments: The first reversible redox pair ($E_{p/2}$ = −0.10 V, vs $Fc^{+/0}$) is photolabile, since a change in the CV was observed after 4 hours of irradiation at 405 nm.

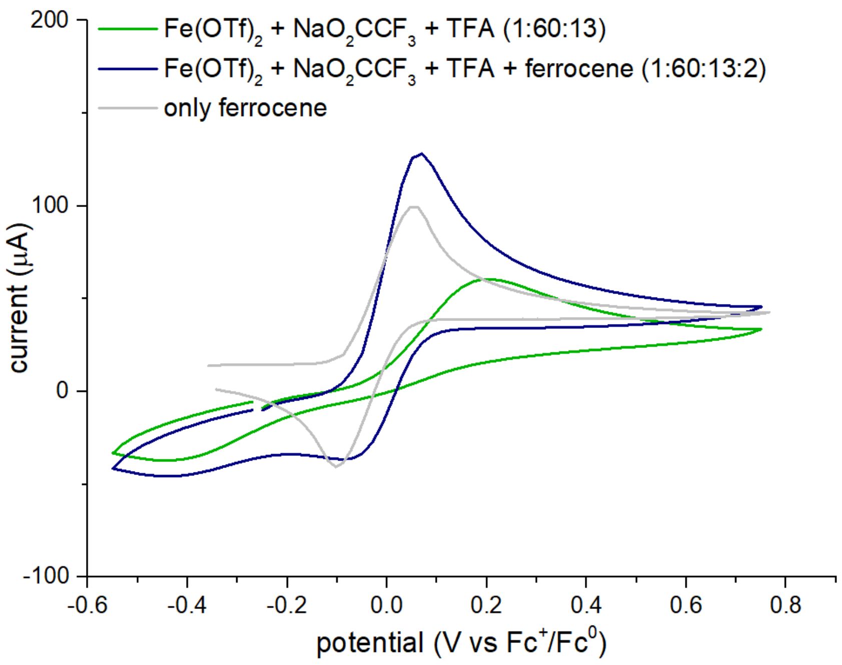

Fig S21. Cyclic voltammograms of: **(i)** $Fe(OTf)_2$ (10 mM), NaO_2CCF_3 (600 mM) and TFA (130 mM) [green trace]; **(ii)** sequential addition of ferrocene to solution **(i)** ($Fc^{+/0}$, 10 mM); and **(iii)** ferrocene (10 mM *ca.*).

***<u>Comments</u>*:** The reduction current of Fc is significantly lower than the oxidation current, indicating that Fc is able to oxidize non-ligated $Fe^{II}(O_2CCF_3)_n$ species.[11,12]

Cyclic voltammograms of the individual components

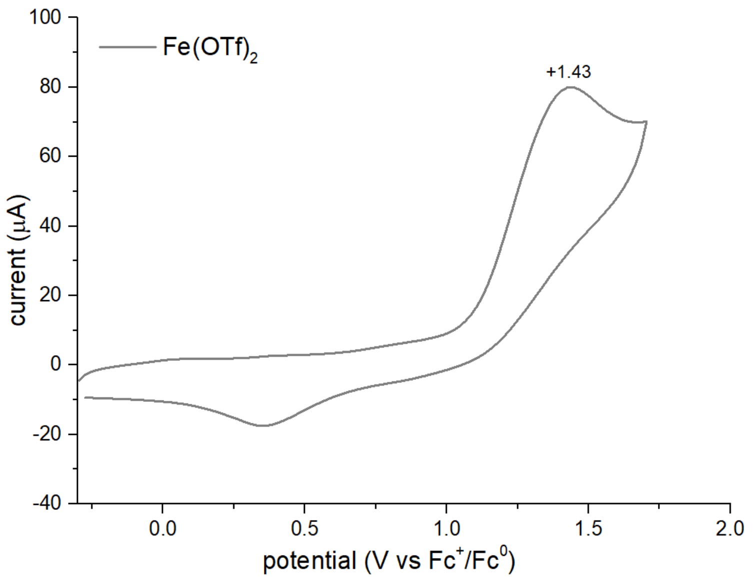

Fig. S22. Cyclic voltammogram of $Fe(OTf)_2$ (10 mM); in 0.1 M $TBAPF_6$ in CH_3CN.

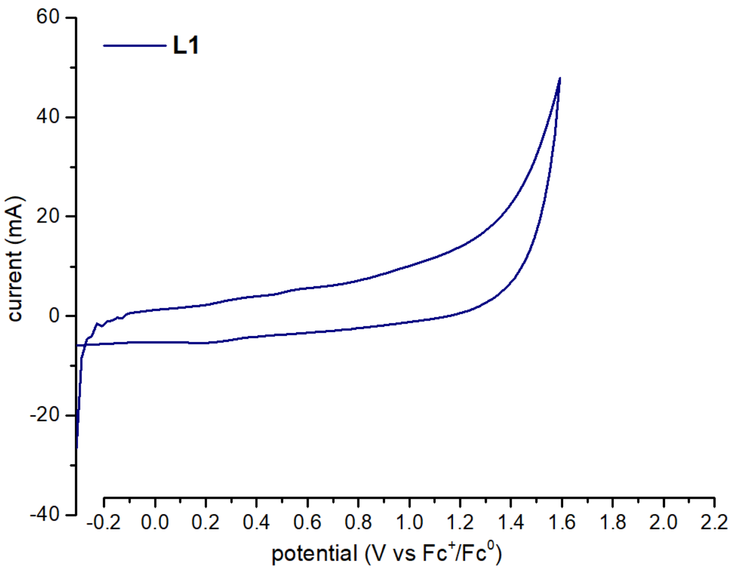

Fig. S23. Cyclic voltammogram of **L1** (10 mM); in 0.1 M $TBAPF_6$ in CH_3CN.

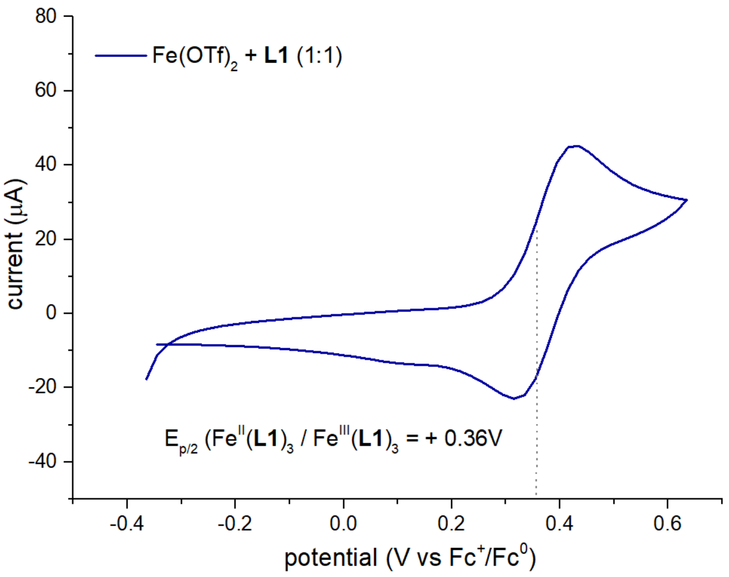

Fig. S24. Cyclic voltammogram of $Fe(OTf)_2$ (10 mM) and *p*-MeO-BPY ligand **L1**; in 0.1 M $TBAPF_6$ in CH_3CN.

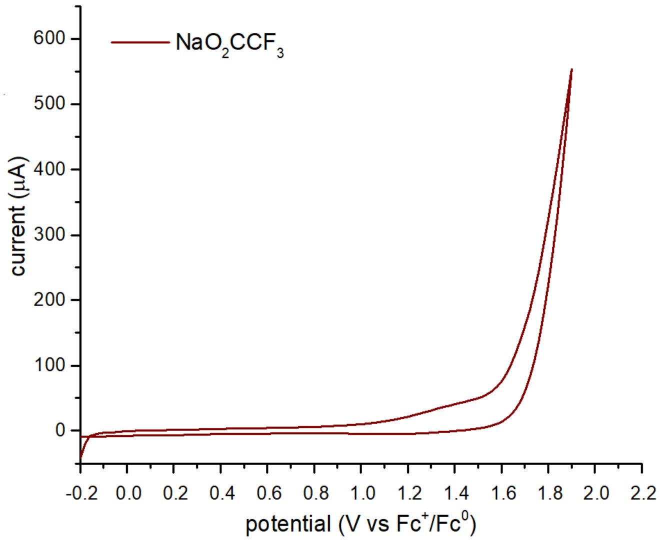

Fig. S25. Cyclic voltammogram of NaO_2CCF_3 (600 mM); 0.1 M $TBAPF_6$ in CH_3CN

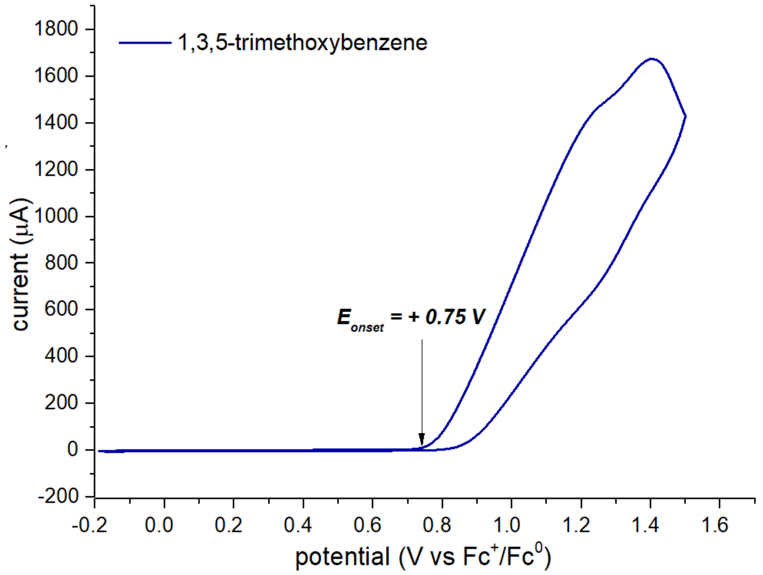

Fig. S26. Cyclic voltammogram of 1,3,5-trimethoxybenzene (100 mM); in 0.1 M TBAPF$_6$ in CH_3CN.

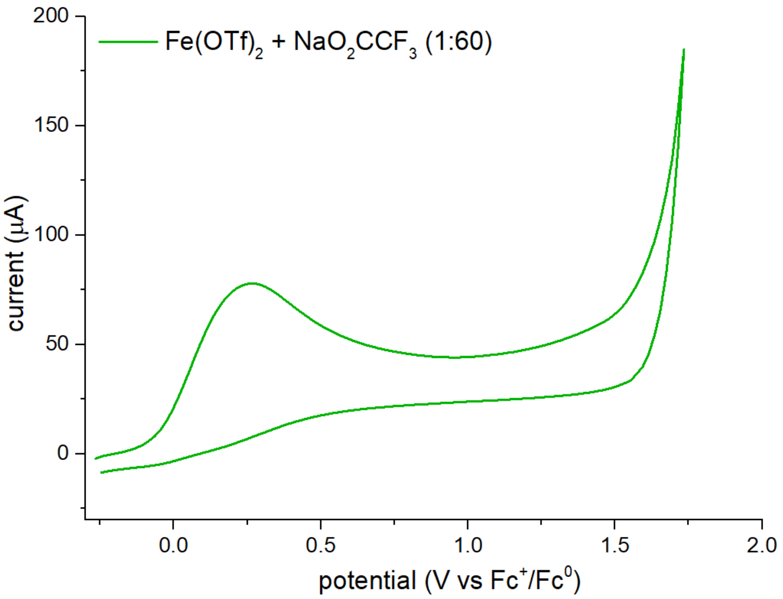

Fig. S27. Cyclic voltammogram of $Fe(OTf)_2$ (10 mM) and NaO_2CCF_3 (600 mM); in 0.1 M TBAPF$_6$ in CH_3CN.

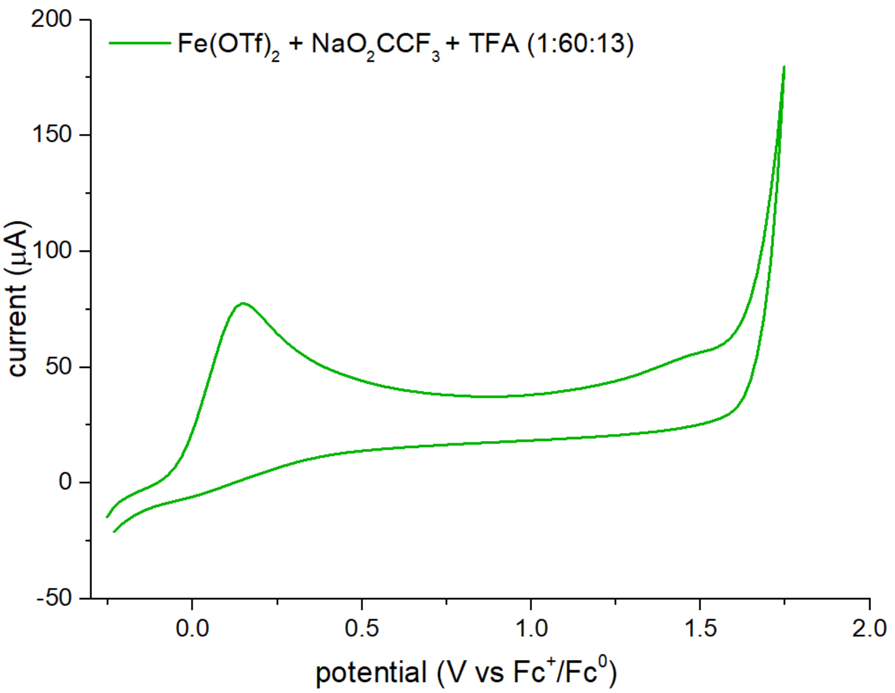

Fig. S28. Cyclic voltammogram of $Fe(OTf)_2$ (10 mM), NaO_2CCF_3 (600 mM) and TFA (130 mM); in 0.1 M $TBAPF_6$ in CH_3CN.

HYDROGEN DETECTION

Following the general procedure A, 1,3,5-trimethoxybenzene (71.5 mg, 0.5 mmol, 1 equiv.) and sodium trifluoroacetate (408 mg, 3 mmol, 6.0 equiv.) were used. After the reaction was completed, a sample of the headspace (1 mL) was collected using a gas syringe. The sample was analyzed in a Gas Chromatograph 8890 Agilent (thermal conductivity detector, TCD; column temperature: 40 ºC; injector temperature: 50 ºC; flow gas carrier 1 mL/min He; column: Molisieve 5A; volume of injection: 250 µL.). The analysis of the sample (Figure S30) was compared with a blank (Figure S28) and a sample containing pure hydrogen (Figure S29). *Note:* the observed N_2 and O_2 signals are attributed to atmospheric air that cannot be fully excluded during injection.

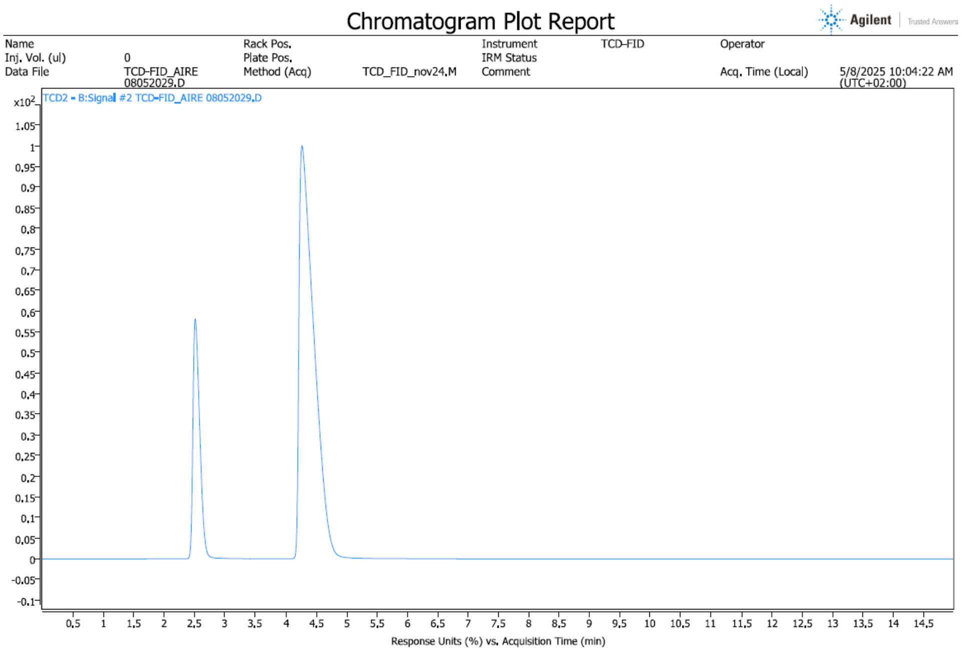

Fig. S29. Gas chromatogram of air (blank sample).

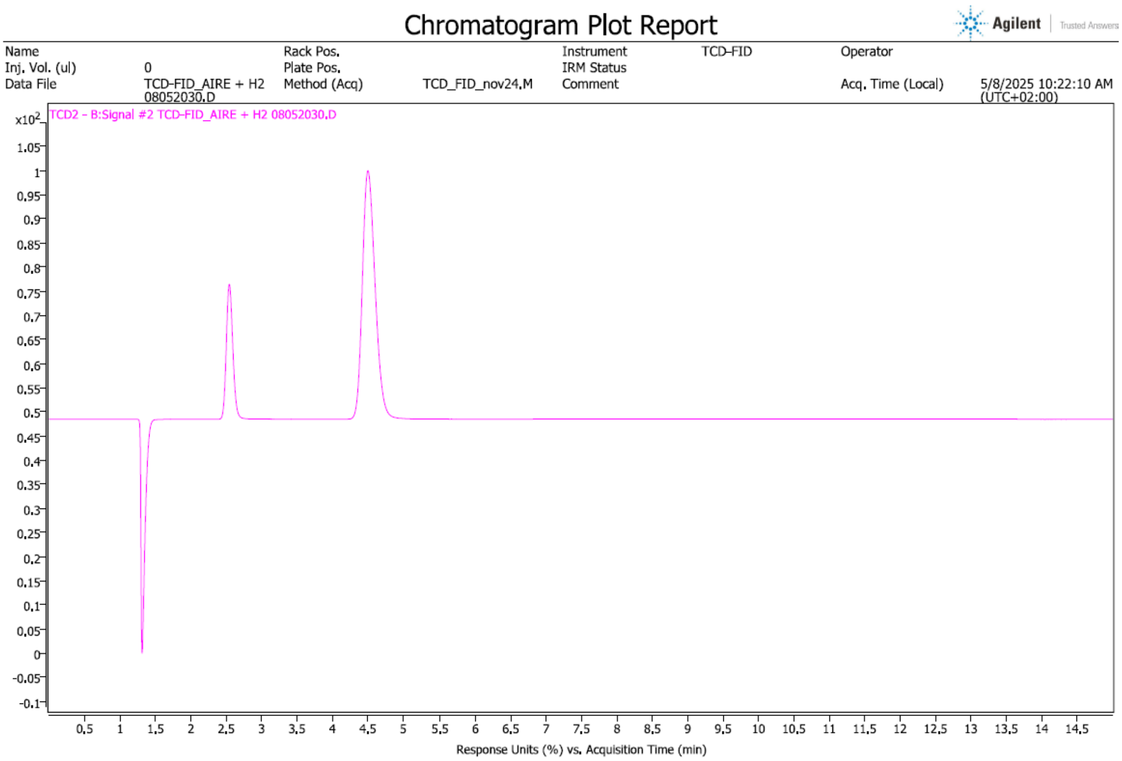

Fig. S30. Gas chromatogram of a reference sample containing air and hydrogen (reference sample).

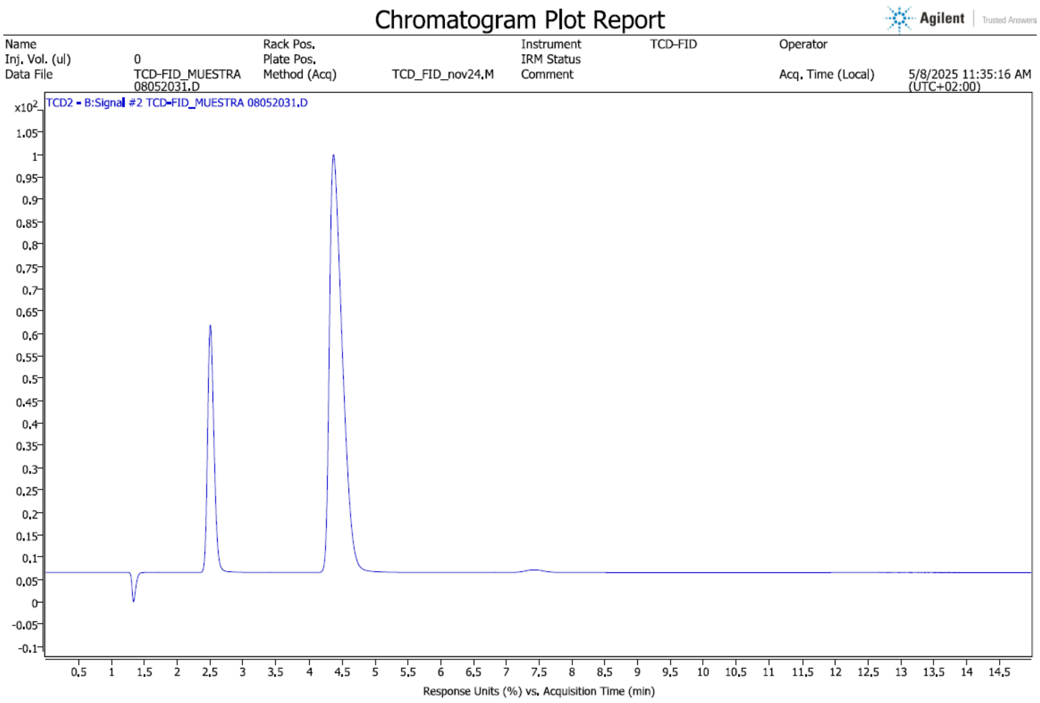

Fig. S31. Gas chromatogram of the headspace sampled from the reaction vessel.

EVALUATION OF THE REACTIVITY AT DIFFERENT APPLIED ANODE POTENTIALS

The experiments were performed following the general procedure A, using caffeine (38.8 mg, 0.2 mmol). In each experiment, the anode potential corresponding to the applied cell-potential was determined at the beginning of the reaction using a third reference electrode (Ag/AgCl (0.1 M in KCl)) and a potentiostat connected between the reference electrode and the anode.

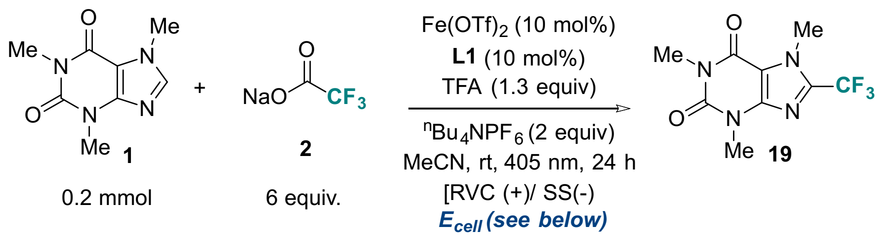

entry	E_{cell} applied(V)	E_{anode} (V vs Fc^+/Fc^0)	Yield (%) of 19
1	0.8	-0.13	0
2	1.2	-0.05	24
3	1.4	0.03	26
4	1.6	0.14	59
5	1.8	0.26	65
6	2.0	0.48	68
7	2.4	0.94	69

Table S6. **Voltage-reactivity relationship studies.** Reaction conditions: caffeine (38.8 mg, 0.2 mmol, 1 equiv.), NaO_2CCF_3 (163.2 mg, 1.2 mmol, 6.0 equiv), $Fe(OTf)_2$ (7.1 mg, 0.02 mmol, 10 mol%), 4,4'-dimethoxy-2,2'-bipyridine **L1** (4.3 mg, 0.02 mmol, 10 mol%) and $TBAPF_6$ (154.9 mg, 0.4 mmol, 2 equiv.), TFA, RVC anode, stainless steel cathode, in 2 mL ACN were stirred (650 rpm) at 35ºC under different constant voltages, using ElectraSyn 2.0 while irradiated with a 405 nm EvoluChem® LED at 3 cm. Yields were determined by ^{19}F NMR using hexafluorobenzene as internal standard.

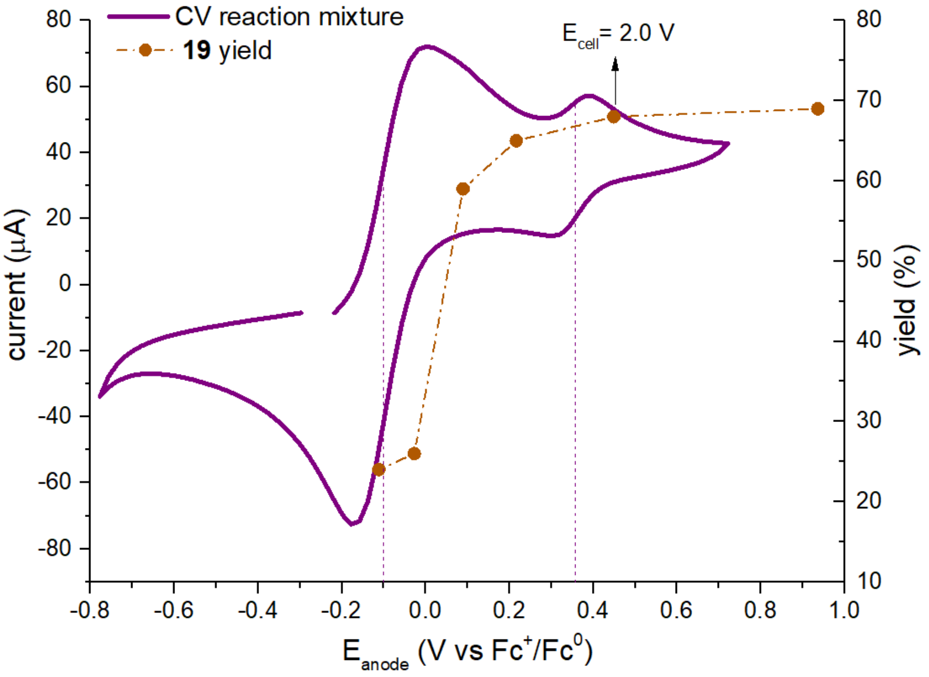

Fig. S32. Graphical overlap between: **(i)** the cyclic voltammetry of the multifunctional iron species (left Y-axis); and **(ii)** the graphical representation of the relationship between anode potential (E_{anode}) *vs* yield of product **19** (right Y-axis).

ATTEMPT AT THE DIRECT PHOTOELECTROTRIFLUOROMETHYLATION WITH TRIFLUOROACETIC ACID

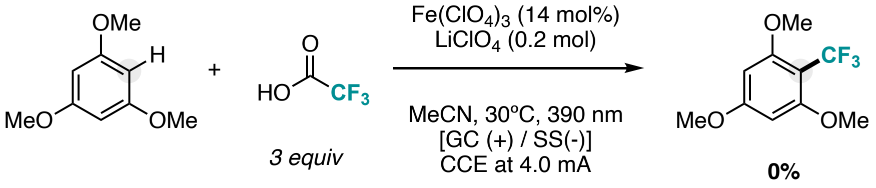

Fig. S33. Attempt at the electrophotochemical C─H fluoroalkylation of heterocycles using trifluoroacetic acid. Reaction conditions reported by Ackermann and co-workers using 1,3,5-trimethoxybenzene (50.5 mg, 0.30 mmol, 1.0 equiv) as substrate.[9] Yield was determined by ^{19}F NMR using hexafluorobenzene as internal standard.

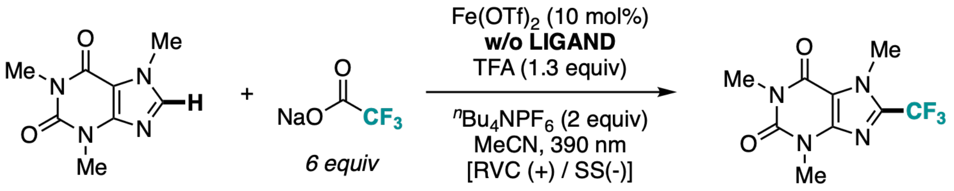

entry	conditions	CF_3-yield
1	E_{cell} = 2.06 V	15%
2	i = 4.0mA	2%
3	+ Fc (10 mol%), E_{cell} = 2.06 V	<5%

Fig. S34. Attempts to trifluoromethylate caffeine without L1. Reaction conditions: caffeine (97.1 mg, 0.30 mmol, 1.0 equiv), NaO_2CCF_3 (245 mg, 3.0 mmol, 6 equiv), $Fe(OTf)_2$ (10.6 mg, 0.03 mmol, 10 mol%), $TBAPF_6$ (232 mg, 0.6 mmol, 2 equiv.) and TFA (30 µL, 0.4 mmol, 1.3 equiv), RVC anode, stainless steel cathode, in 3 mL ACN were stirred (650 rpm) at 35 ºC using ElectraSyn 2.0 while irradiated with a 390nm Kessil LED at 3 cm. [a]Yields were determined by ^{19}F NMR using hexafluorobenzene as internal standard.

IN-SITU HRMS ANALYSIS OF THE REACTION MIXTURE

Samples for ESI-HRMS were prepared using a mixture of $Fe(OTf)_2$ (10 mM), **L1** (10 mM), NaO_2CCF_3 (600 mM) and TFA (130 mM) in MeCN and diluted prior to the analyses.

Analyses were performed using an Agilent TOF/Q-TOF G6546A Mass Spectrometer with direct injection and electrospray ionization in positive and negative mode. The mobile phase consisted of MeOH/water (70:30) with 0.1% formic acid and 5 mM ammonium formate.

These are the ions that we could identify from the mixture which are related to the intermediates proposed in the mechanistic hypothesis:

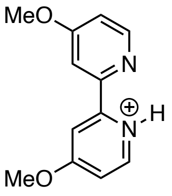

$C_{12}H_{13}N_2O_2^+$

Compound Details

Cpd. 1: C12 H13 N2 O2

Name	Formula	RT	RI	Mass	Diff (Tgt, ppm)	CAS	ID Source	Score	Algorithm
	C12 H13 N2 O2	0.223		217.0980	1.54		FBF	99.44	FBF

Species	m/z	Score (Tgt)	Score (Lib)	Score (DB)	Score (MFG)	Score (RT)
M+	217.0975	99.44				

Compound Spectra (overlaid)

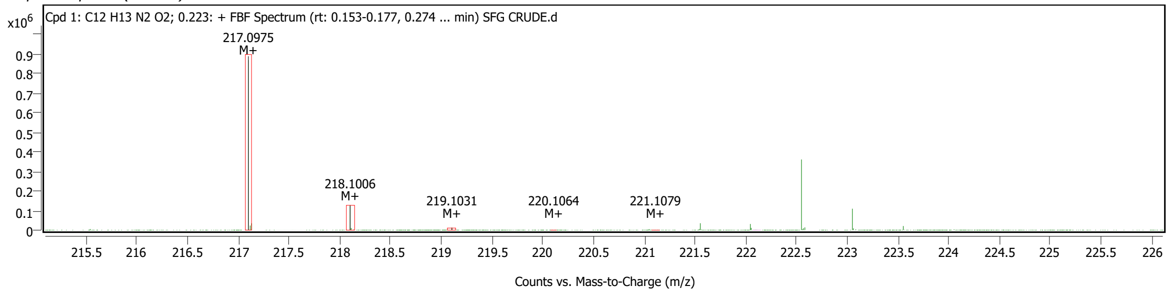

Spectrum Peaks

m/z	Z	Abund	Diff (ppm)	Height %	Height % (Calc)	Ion Species	Formula
217.0975	1	888062	1.52	100.00	100.00	M+	C12H13N2O2
218.1006	1	127391	1.58	14.34	13.94	M+	C12H13N2O2
219.1031	1	12387	1.92	1.39	1.31	M+	C12H13N2O2
220.1064	1	957	5.51	0.11	0.09	M+	C12H13N2O2
221.1079	1	69	0.86	0.01	0.01	M+	C12H13N2O2
217.0975		868499	1.50				

Compound ID Table

Name	Formula	Species	RT	RT Diff	Mass	CAS	ID Source	Score	Score (Lib)	Score (Tgt)
	C12 H13 N2 O2	M+	0.223		217.0980		FBF	99.44		99.44

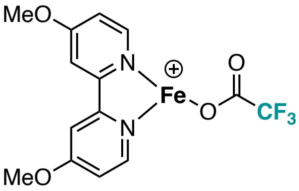

$C_{14}H_{12}F_3FeN_2O_4^+$

Compound Details

Cpd. 1: C14 H12 F3 Fe N2 O4

Name	Formula	RT	RI	Mass	Diff (Tgt, ppm)	CAS	ID Source	Score	Algorithm
	C14 H12 F3 Fe N2 O4	0.223		383.0146	0.11		FBF	94.10	FBF

Species	m/z	Score (Tgt)	Score (Lib)	Score (DB)	Score (MFG)	Score (RT)
M+	383.0140	94.10				

Compound Spectra (overlaid)

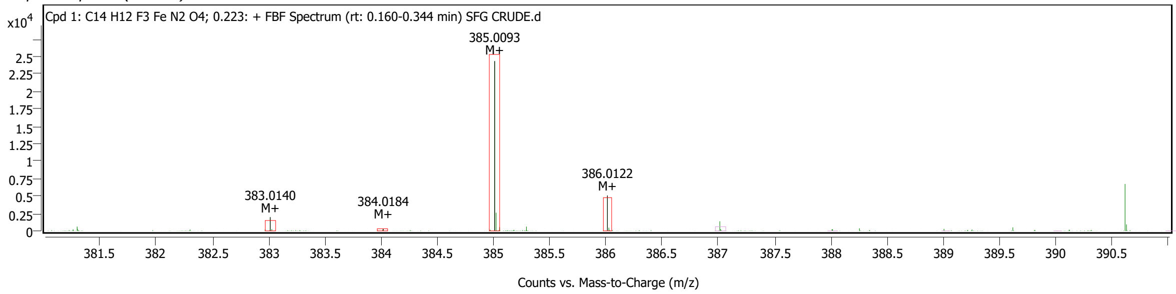

Spectrum Peaks

m/z	Z	Abund	Diff (ppm)	Height %	Height % (Calc)	Ion Species	Formula
383.0140	1	1968	0.11	8.08	6.36	M+	C14H12F3FeN2O4
384.0184	1	277	3.46	1.14	1.03	M+	C14H12F3FeN2O4
385.0093	1	24340	0.04	100.00	100.00	M+	C14H12F3FeN2O4
386.0122	1	5079	0.26	20.87	18.46	M+	C14H12F3FeN2O4

Compound ID Table

Name	Formula	Species	RT	RT Diff	Mass	CAS	ID Source	Score	Score (Lib)	Score (Tgt)
	C14 H12 F3 Fe N2 O4	M+	0.223		383.0146		FBF	94.10		94.10

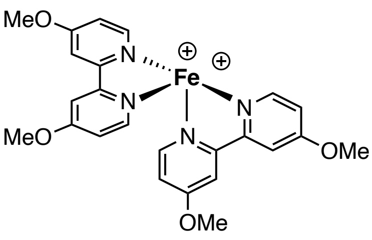

$C_{24}H_{24}FeN_4O_4^{2+}$

Compound Details

Cpd. 1: C24 H24 Fe N4 O4

Name	Formula	RT	RI	Mass	Diff (Tgt, ppm)	CAS	ID Source	Score	Algorithm
	C24 H24 Fe N4 O4	0.514		486.1197	0.71		FBF	99.05	FBF

Species	m/z	Score (Tgt)	Score (Lib)	Score (DB)	Score (MFG)	Score (RT)
M+2 (M+2H)+2	243.0592 245.0672	99.05				

Compound Spectra (overlaid)

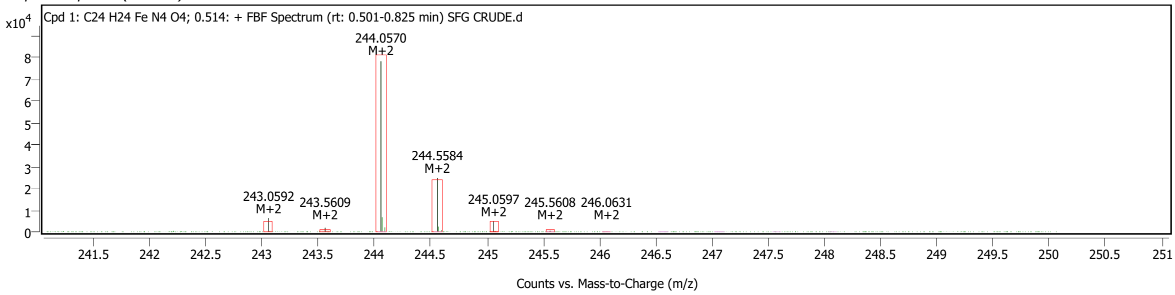

Spectrum Peaks

m/z	Z	Abund	Diff (ppm)	Height %	Height % (Calc)	Ion Species	Formula
243.0592	2	6285	0.25	8.04	6.35	M+2	C24H24FeN4O4
243.5609	2	1863	1.02	2.38	1.77	M+2	C24H24FeN4O4
244.0570	2	78197	0.63	100.00	100.00	M+2	C24H24FeN4O4
244.5584	2	24774	0.87	31.68	30.10	M+2	C24H24FeN4O4
245.0597	2	4947	1.38	6.33	5.49	M+2	C24H24FeN4O4
245.0672	2	56	10.30	100.00	100.00	(M+2H)+2	C24H24FeN4O4
245.5608	2	464	1.43	0.59	0.74	M+2	C24H24FeN4O4
246.0631	2	92	6.39	0.12	0.08	M+2	C24H24FeN4O4

Compound ID Table

Name	Formula	Species	RT	RT Diff	Mass	CAS	ID Source	Score	Score (Lib)	Score (Tgt)
	C24 H24 Fe N4 O4	M+2 (M+2H)+2	0.514		486.1197		FBF	99.05		99.05

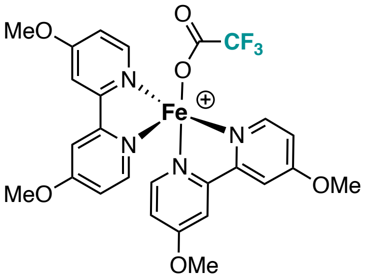

$C_{26}H_{24}F_3FeN_4O_6^+$

Compound Details

Cpd. 1: C26 H24 F3 Fe N4 O6

Name	Formula	RT	RI	Mass	Diff (Tgt, ppm)	CAS	ID Source	Score	Algorithm
	C26 H24 F3 Fe N4 O6	0.223		599.1046	0.35		FBF	97.99	FBF

Species	m/z	Score (Tgt)	Score (Lib)	Score (DB)	Score (MFG)	Score (RT)
M+	599.1037	97.99				

Compound Spectra (overlaid)

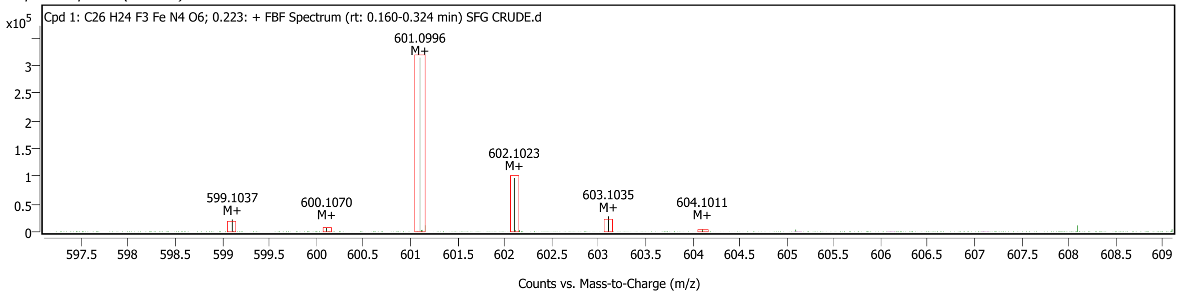

Spectrum Peaks

m/z	Z	Abund	Diff (ppm)	Height %	Height % (Calc)	Ion Species	Formula
599.1037	1	22101	-0.29	7.03	6.35	M+	C26H24F3FeN4O6
600.1070	1	6978	0.15	2.22	1.91	M+	C26H24F3FeN4O6
601.0996	1	314505	0.61	100.00	100.00	M+	C26H24F3FeN4O6
602.1023	1	97342	0.32	30.95	32.33	M+	C26H24F3FeN4O6
603.1035	1	27734	-1.36	8.82	6.59	M+	C26H24F3FeN4O6
604.1011	1	1606	-9.15	0.51	1.00	M+	C26H24F3FeN4O6

Compound ID Table

Name	Formula	Species	RT	RT Diff	Mass	CAS	ID Source	Score	Score (Lib)	Score (Tgt)
	C26 H24 F3 Fe N4 O6	M+	0.223		599.1046		FBF	97.99		97.99

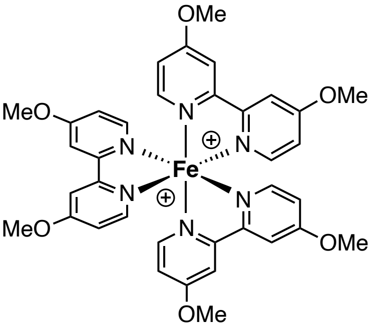

$C_{36}H_{36}FeN_6O_6^{2+}$

Compound Details

Cpd. 1: C36 H36 Fe N6 O6

Name	Formula	RT	RI	Mass	Diff (Tgt, ppm)	CAS	ID Source	Score	Algorithm
	C36 H36 Fe N6 O6	0.230		702.2098	0.73		FBF	98.91	FBF

Species	m/z	Score (Tgt)	Score (Lib)	Score (DB)	Score (MFG)	Score (RT)
M+2	351.1042	98.91				

Compound Spectra (overlaid)

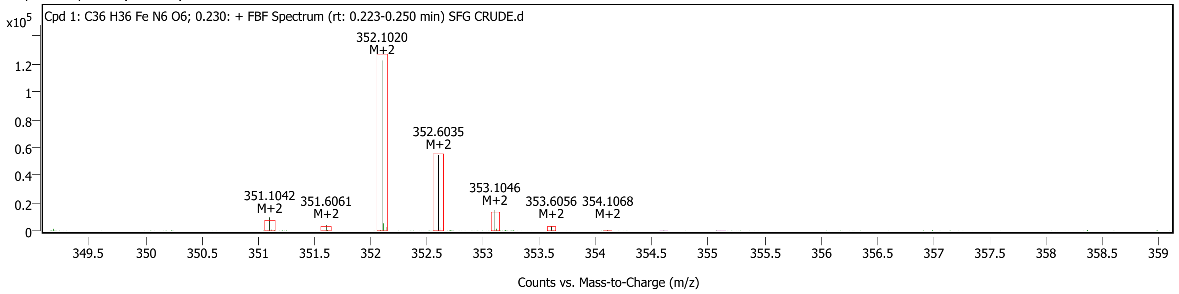

Spectrum Peaks

m/z	Z	Abund	Diff (ppm)	Height %	Height % (Calc)	Ion Species	Formula
351.1042	2	9625	0.35	7.85	6.33	M+2	C36H36FeN6O6
351.6061	2	4110	1.38	3.35	2.64	M+2	C36H36FeN6O6
352.1020	2	122605	0.76	100.00	100.00	M+2	C36H36FeN6O6
352.6035	2	54666	0.83	44.59	43.91	M+2	C36H36FeN6O6
353.1046	2	15067	0.38	12.29	10.96	M+2	C36H36FeN6O6
353.6056	2	3446	-0.27	2.81	1.98	M+2	C36H36FeN6O6
354.1068	2	545	0.01	0.44	0.29	M+2	C36H36FeN6O6

Compound ID Table

Name	Formula	Species	RT	RT Diff	Mass	CAS	ID Source	Score	Score (Lib)	Score (Tgt)
	C36 H36 Fe N6 O6	M+2	0.230		702.2098		FBF	98.91		98.91

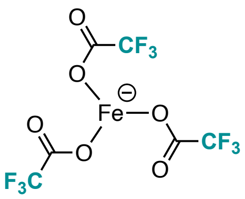

$C_6F_9FeO_6^-$

Compound Details

Cpd. 1: C6 F9 Fe O6

Name	Formula	RT	RI	Mass	Diff (Tgt, ppm)	CAS	ID Source	Score	Algorithm
	C6 F9 Fe O6	0.258		392.8947	0.04		FBF	96.98	FBF

Species	m/z	Score (Tgt)	Score (Lib)	Score (DB)	Score (MFG)	Score (RT)
M-	392.8951	96.98				

Compound Spectra (overlaid)

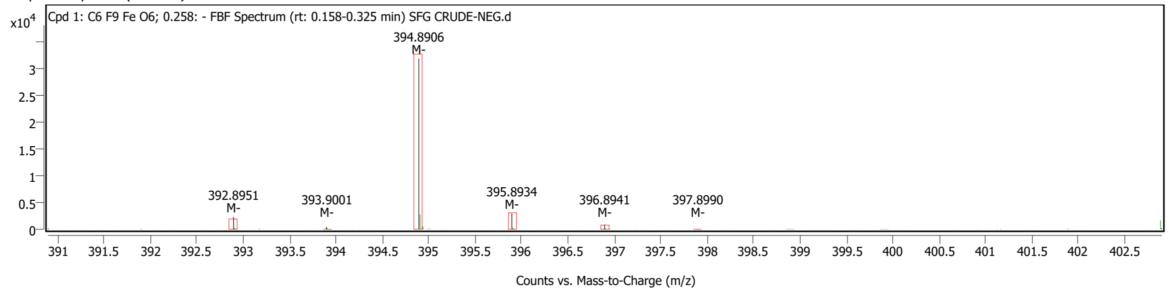

Spectrum Peaks

m/z	Z	Abund	Diff (ppm)	Height %	Height % (Calc)	Ion Species	Formula
392.8951	1	2294	-0.56	7.19	6.36	M-	
393.9001	1	286	3.58	0.89	0.43	M-	
394.8906	1	31932	0.02	100.00	100.00	M-	
395.8934	1	2968	0.28	9.30	9.02	M-	
396.8941	1	880	-0.06	2.76	1.88	M-	
397.8990	1	59	5.51	0.18	0.14	M-	

Compound ID Table

Name	Formula	Species	RT	RT Diff	Mass	CAS	ID Source	Score	Score (Lib)	Score (Tgt)
	C6 F9 Fe O6	M-	0.258		392.8947		FBF	96.98		96.98

ANALYSIS OF THE PHOTODECARBOXYLATION OF Fe(III) SPECIES

Analyses by UV-Visible absorption spectroscopy were performed to examine the photoinduced decarboxylation of Fe(III) species in the presence and absence of the ligand **L1**.

<u>Photodecarboxylation of non-ligated Fe(III) trifluoroacetate species (absence of **L1**)</u>

A mixture of $Fe(OTf)_3$ (10 mM), NaO_2CCF_3 (600 mM), and TFA (130 mM) in dry acetonitrile was irradiated at 405 nm, and the corresponding UV-visible spectra were recorded by taking an aliquot of 50 µL and diluting it in 2 mL with acetonitrile at different irradiation times.

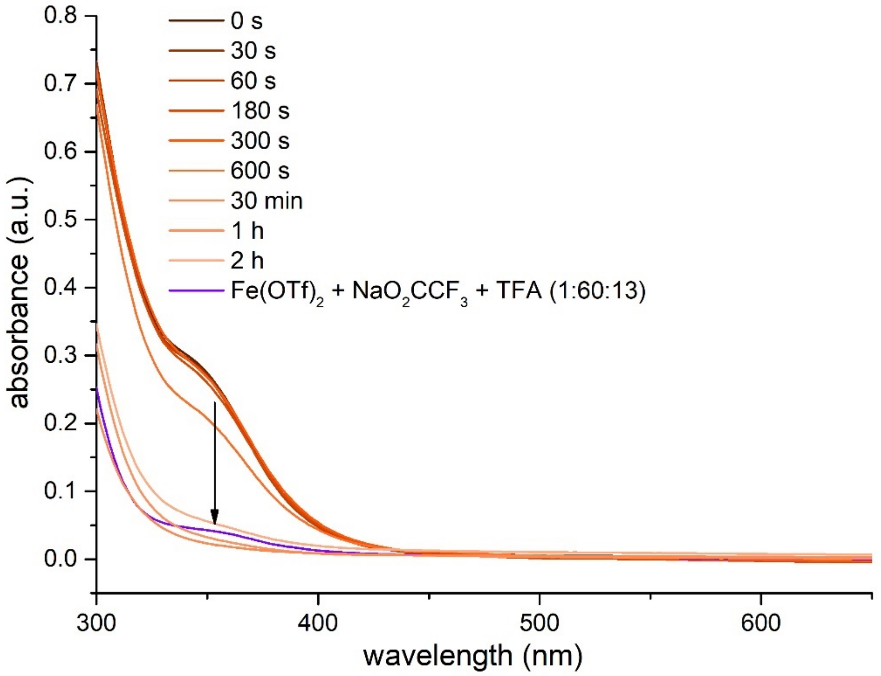

Figure S35: (a) UV-Vis spectra of a mixture of $Fe(OTf)_3$ (10 mM), NaO_2CCF_3 (600 mM) and TFA (130 mM) in dry acetonitrile, at different irradiation times (irradiation at 405 nm) [*orange gradient*]; (b) UV-Vis spectrum of a mixture of $Fe(OTf)_2$ (10 mM), NaO_2CCF_3 (600 mM) and TFA (130 mM) [*purple line*].

Comment: After 30 minutes of irradiation, the Fe(III) trifluoroacetate species associated with intermediate **IV** was completely consumed, as evidenced by the disappearance of the absorption band at 350 nm. Concomitantly, Fe(II) trifluoroacetate species were formed, as confirmed by comparison with the purple reference spectrum. The photoreduction of Fe(III) to Fe(II) arises from the decarboxylative LMCT process.

Photodecarboxylation of ligated Fe(III) trifluoroacetate species (with **L1**)

A mixture of $Fe(OTf)_3$ (10 mM), **L1** (10 mM), NaO_2CCF_3 (600 mM), and TFA (130 mM) in dry acetonitrile was irradiated at 405 nm, and the corresponding UV-visible spectra were recorded by taking an aliquot of 50 µL and diluting it in 2 mL with acetonitrile at different irradiation times.

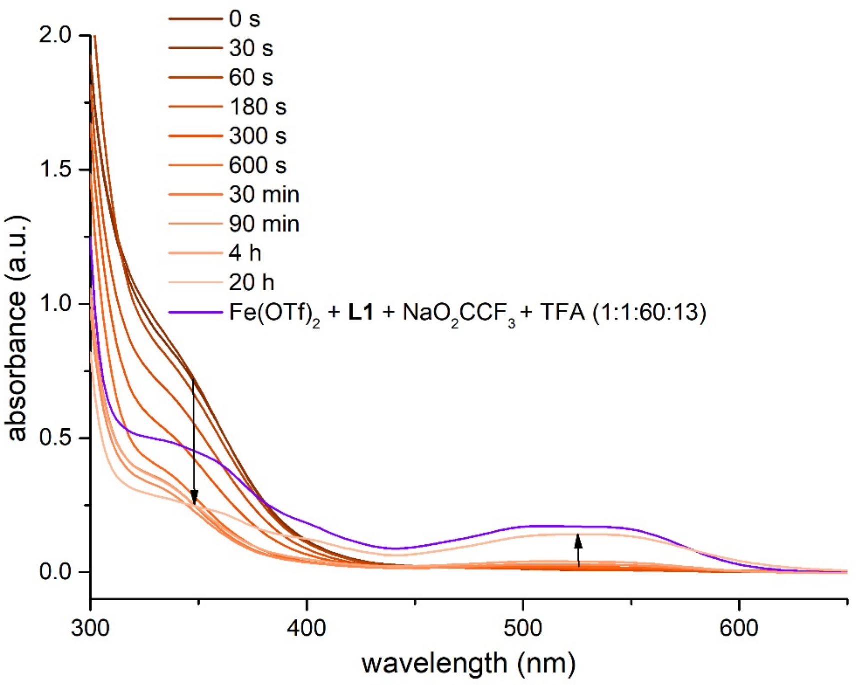

Figure S36: (a) UV-Vis spectra of a mixture of $Fe(OTf)_3$ (10 mM), **L1** (10 mM), NaO_2CCF_3 (600 mM) and TFA (130 mM) in dry acetonitrile, at different irradiation times (irradiation at 405 nm) [*orange gradient*]; (b) UV-Vis spectrum of a mixture of $Fe(OTf)_2$ (10 mM), **L1** (10 mM), NaO_2CCF_3 (600 mM) and TFA (130 mM) [*purple line*].

Comments: Photoreduction of the ligated Fe(III) trifluoroacetate species is evidenced by the emergence of an absorption band in the 500–600 nm region, which is attributed to Fe(II) intermediates based on comparison with the purple reference spectrum. Notably, in the presence of **L1** this LMCT decarboxylation proceeds much more slowly than for the corresponding non-ligated species, requiring approximately 20 h to reach completion.

a) 0 min b) 30 min

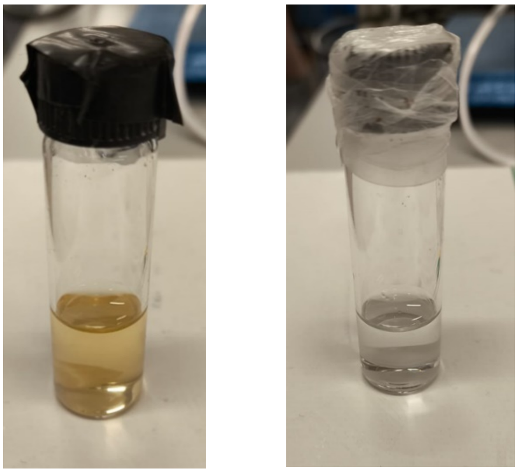

Figure S37: Reaction color at (a) t = 0 s (left); (b) 30 min of irradiation at 405 nm (right). Reaction conditions: $Fe(OTf)_3$ (10 mM), NaO_2CCF_3 (600 mM) and TFA (130 mM), in dry acetonitrile.

a) 0 min b) 30 min c) 20 h

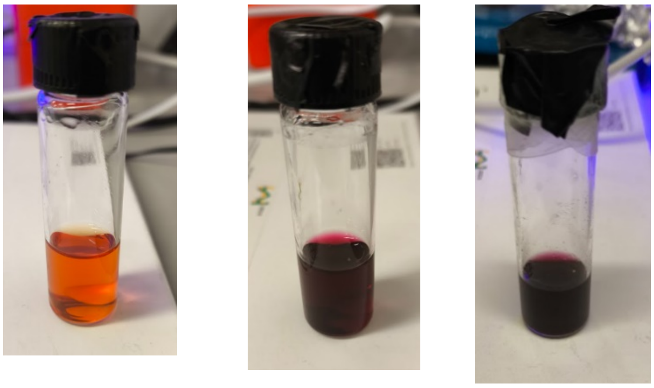

Figure S38: Reaction color at (a) t = 0 s (left); (b) 30 min (middle); (c) 20 h (right). Reaction conditions: $Fe(OTf)_3$ (10 mM), **L1** (10 mM), NaO_2CCF_3 (600 mM) and TFA (130 mM), in dry acetonitrile. Irradiation at 405 nm.

To estimate the photoinduced LMCT event from non-ligated Fe(III) trifluoroacetate species (**IV**) (without **L1**) to the corresponding Fe(II) intermediates (**I**), the absorbance at 350 nm was selected in Figure S35 (vide supra). The % photoreduction was calculated as follows:

$$\%\, photoreduction\ \boldsymbol{IV}\ to\ \boldsymbol{I} = \frac{A_{max} - A_t}{A_{max} - A_{min}}$$

where A_t is the absorbance at a certain time, A_{max} is the absorbance of the Fe(III) mixture before irradiation, and A_{min} is the absorbance of the Fe(II) mixture. All of them at 350 nm.

On the other hand, to estimate the photoinduced LMCT event from ligated Fe(III) trifluoroacetate species in the presence of ligand **L1** (**V**) to the corresponding Fe(II) intermediates (**II**), the absorbance at 542 nm was selected in Figure S36 (vide supra). The % photoreduction was calculated as follows:

$$\%\, photoreduction\ \boldsymbol{V}\ to\ \boldsymbol{II} = \frac{A_t - A_{min}}{A_{max} - A_{min}}$$

where A_t is the absorbance at a certain time, A_{min} is the absorbance of the Fe(III) mixture before irradiation, and A_{max} is the absorbance of the Fe(II) mixture. All of them at 542 nm.

With this data in hand, the photoreduction of Fe(III) species **V** and **IV** were plotted vs time in the following figure. As observed after only 30 min, Fe(III) species **IV** are completely transformed into the corresponding Fe(II) species **I**, whereas only an estimated 16 % of Fe(III) species **V** are photoreduced to Fe(II) intermediates **II**.

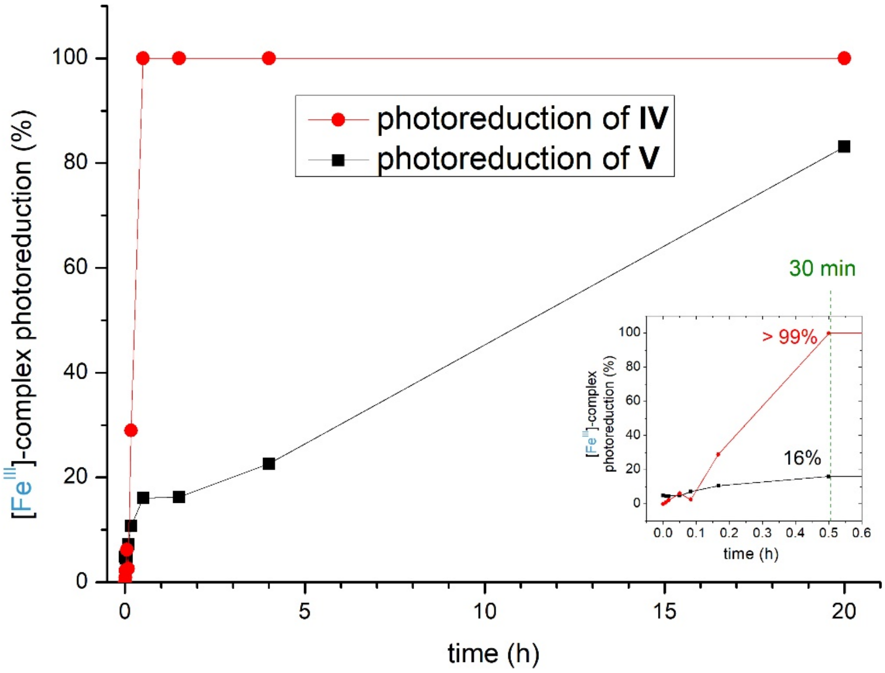

Figure S39: Photoreduction of Fe(III) species in the presence and in the absence of ligand **L1**. The inset shows zoomed data up to 0.5 h, indicating the photoreduction of both Fe(III) species after 30 min.

NMR Spectral Data

^{1}H-NMR (400 MHz, $CDCl_3$) of 2

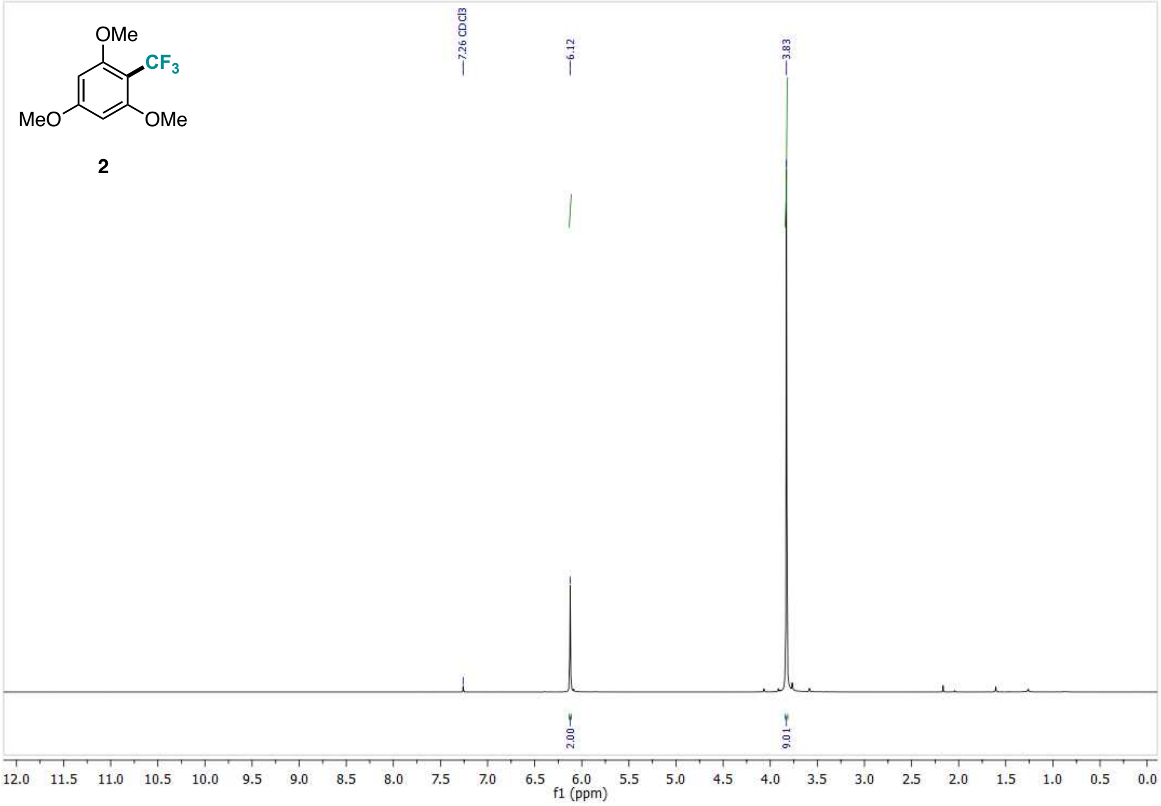

^{13}C-NMR (101 MHz, $CDCl_3$) of 2

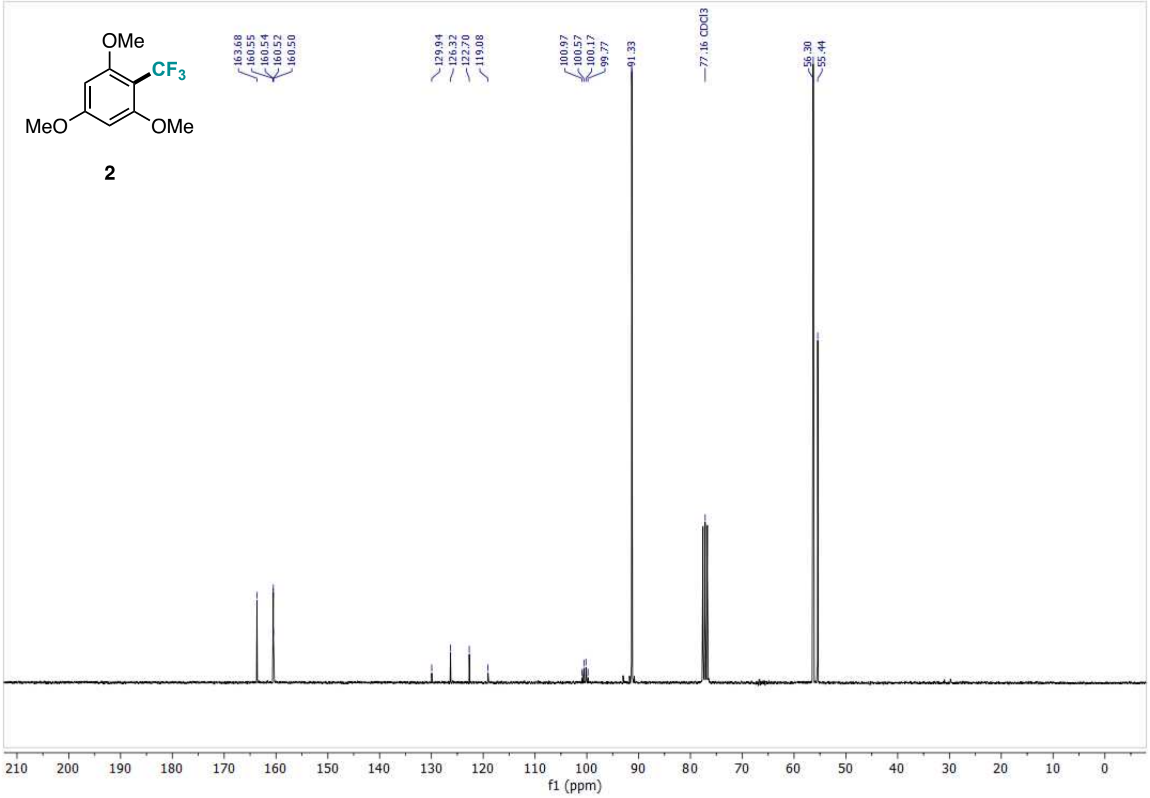

^{19}F-NMR (376 MHz, $CDCl_3$) of 2

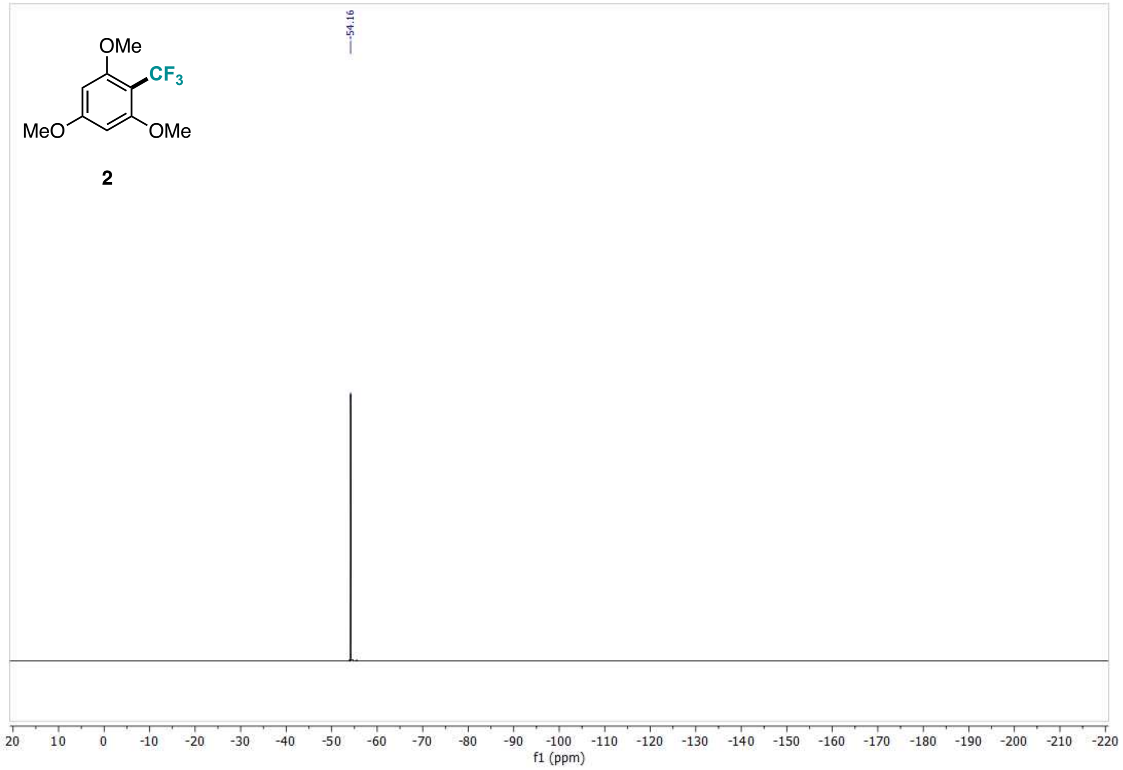

^{1}H-NMR (400 MHz, $CDCl_3$) of 3

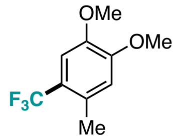

3

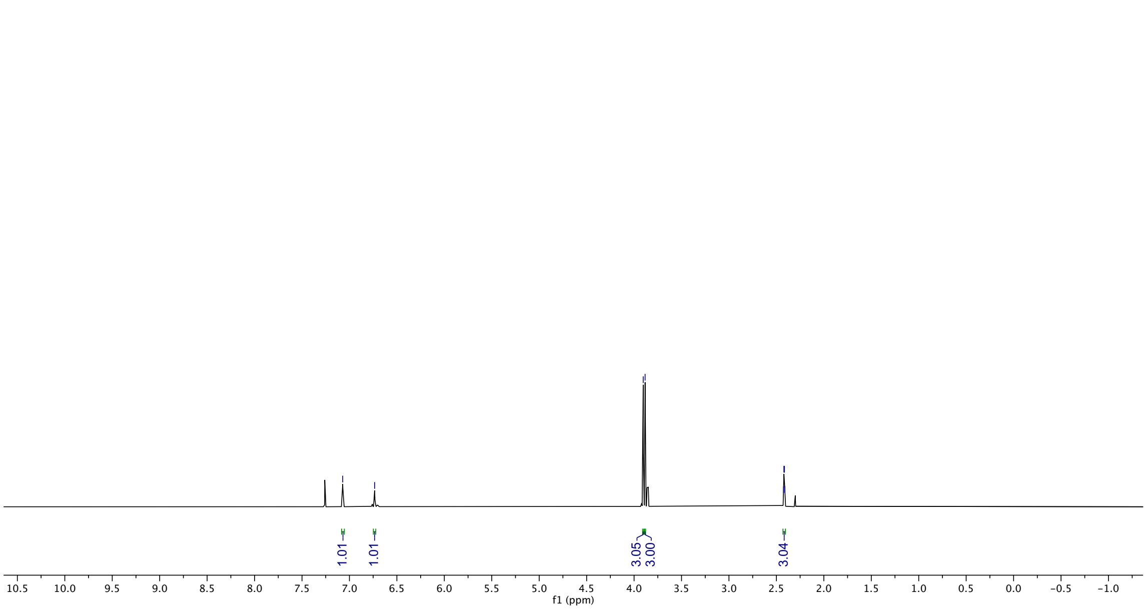

^{13}C-NMR (101 MHz, $CDCl_3$) of 3

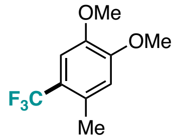

3

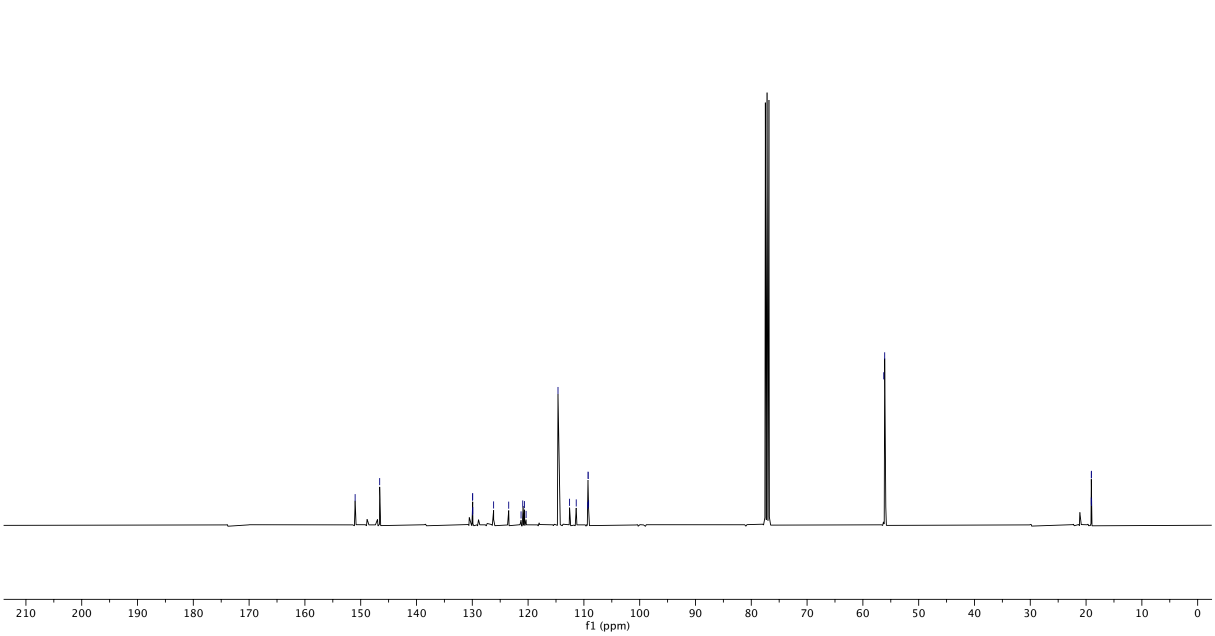

^{19}F-NMR (376 MHz, $CDCl_3$) of 3

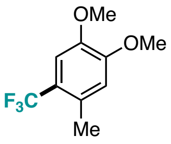

3

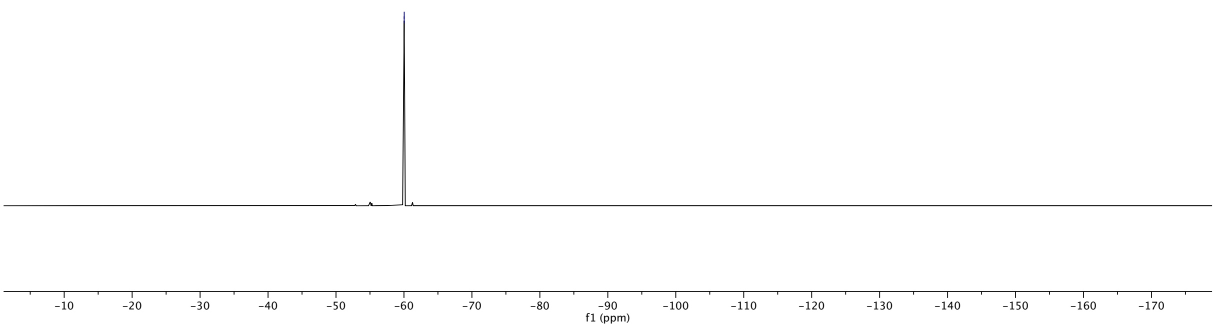

^{1}H-NMR (400 MHz, CDCl$_3$) of 4

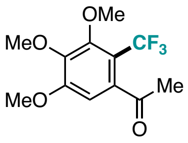

4

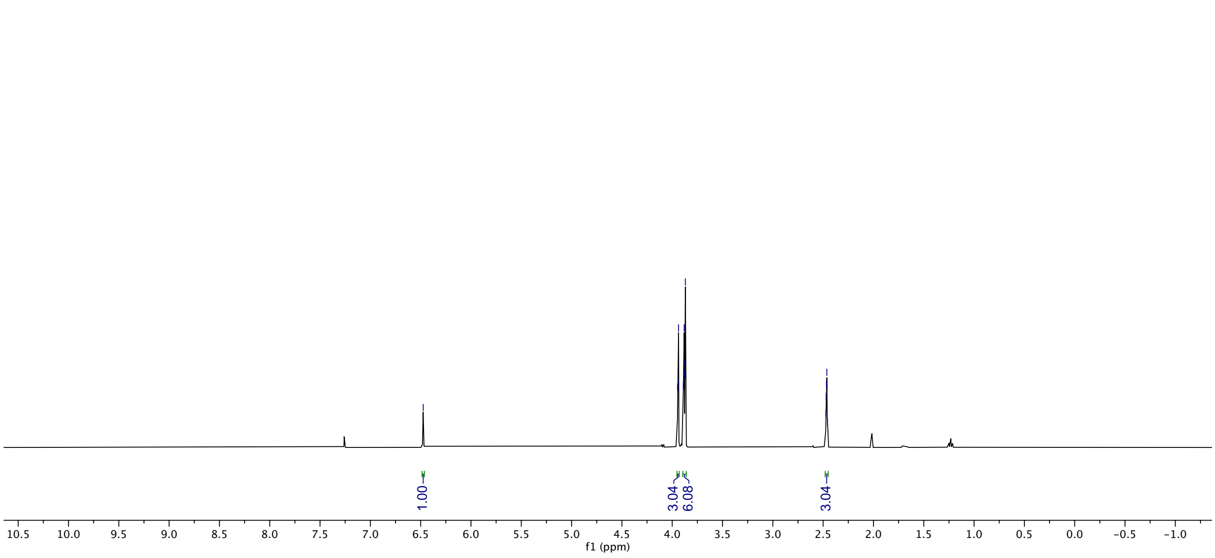

^{13}C-NMR (101 MHz, CDCl$_3$) of 4

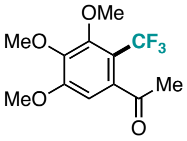

4

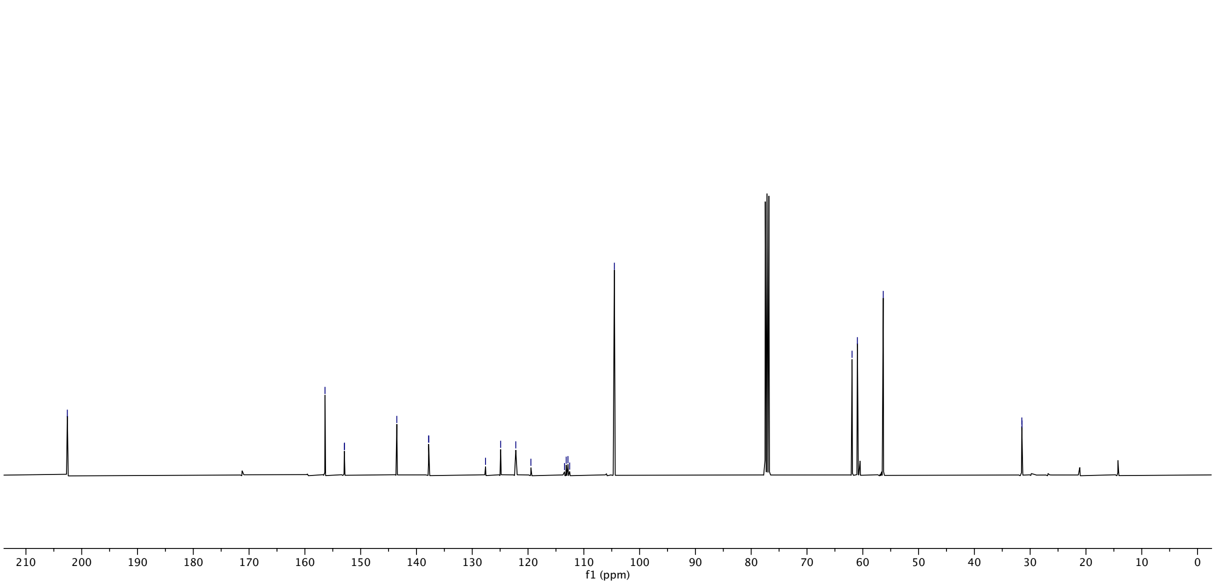

^{19}F-NMR (376 MHz, $CDCl_3$) of 4

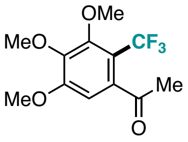

4

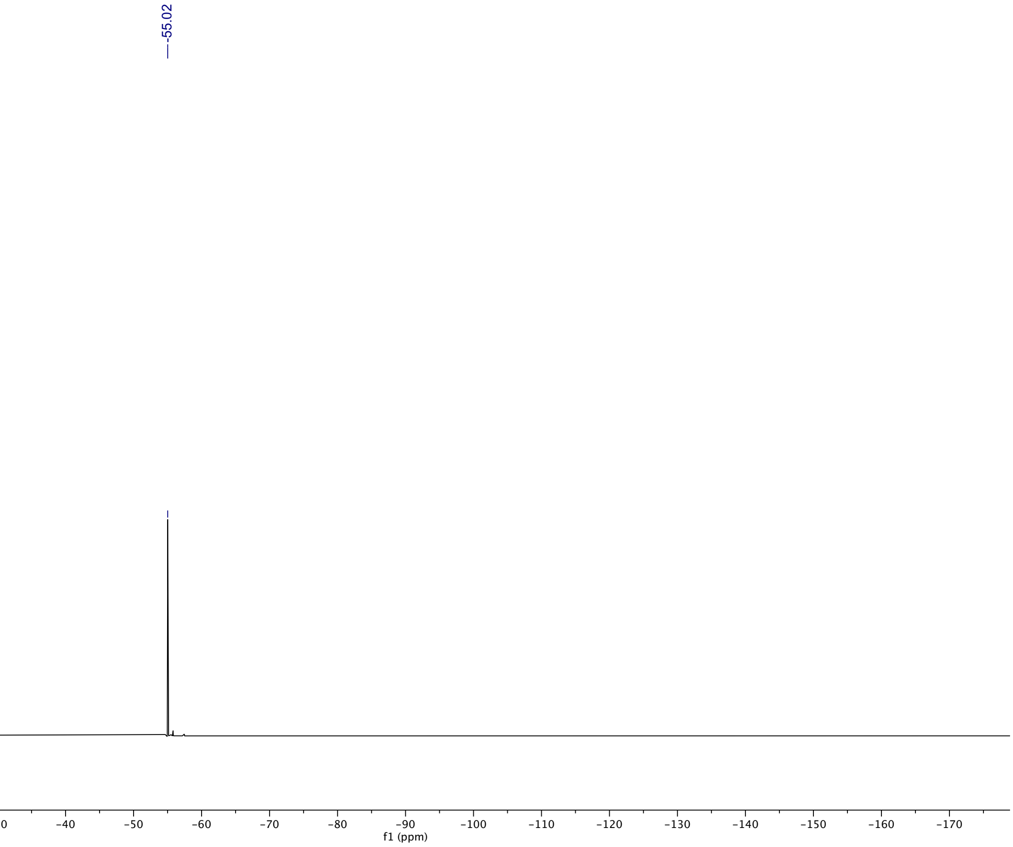

^{1}H-NMR (400 MHz, $CDCl_3$) of 6

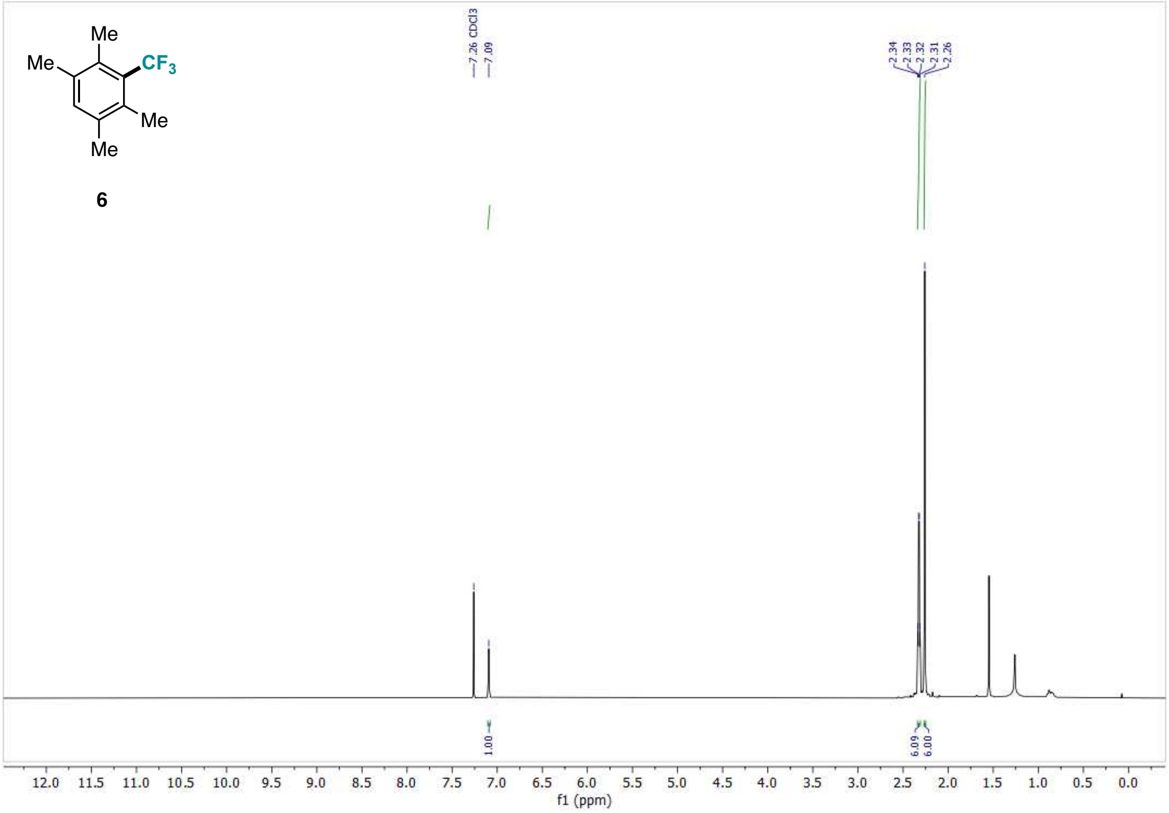

^{13}C-NMR (101 MHz, $CDCl_3$) of 6

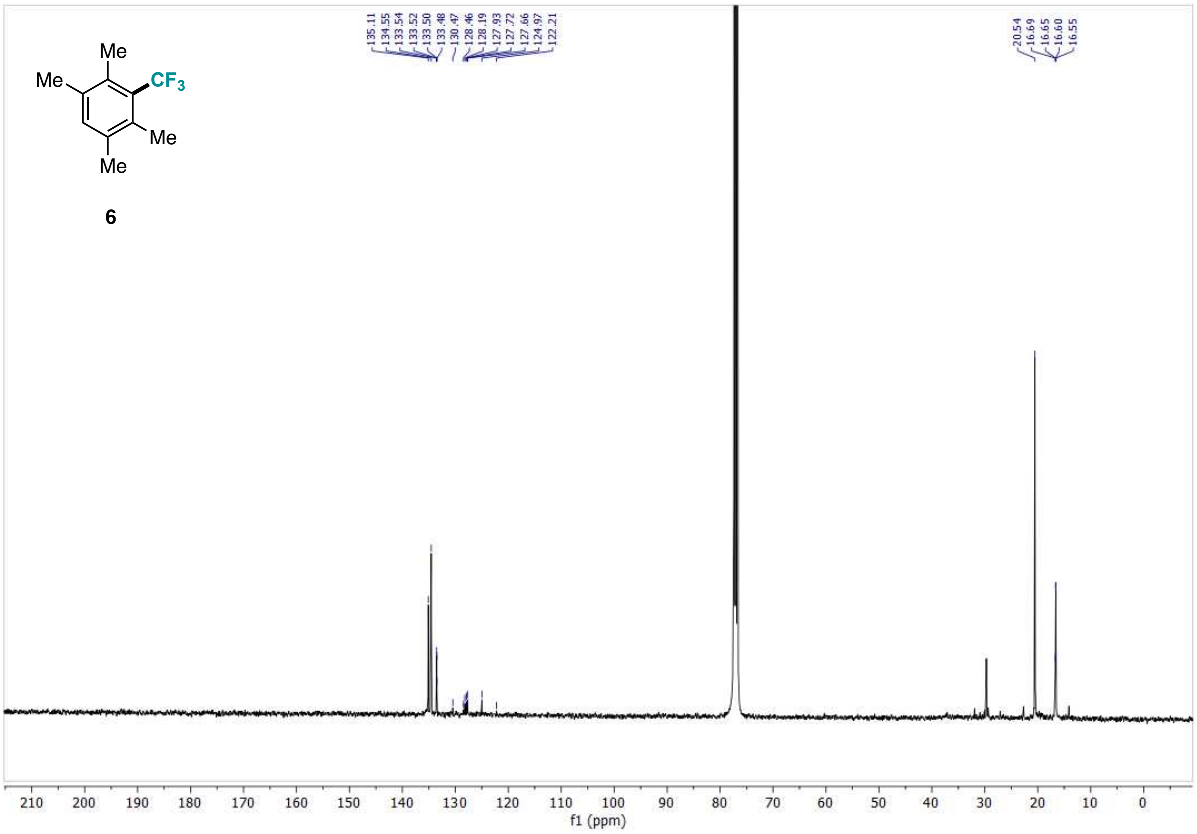

^{19}F-NMR (376 MHz, $CDCl_3$) of 6

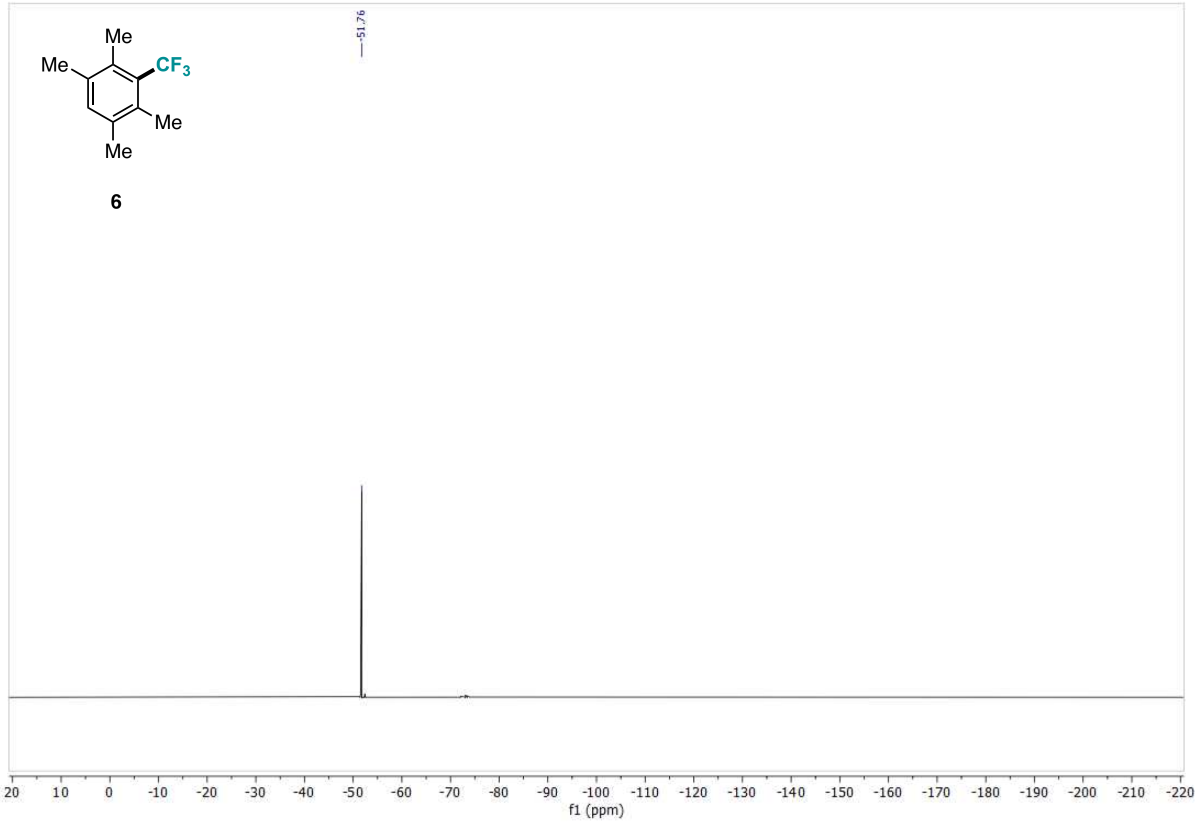

^{1}H-NMR (400 MHz, $CDCl_3$) of 7

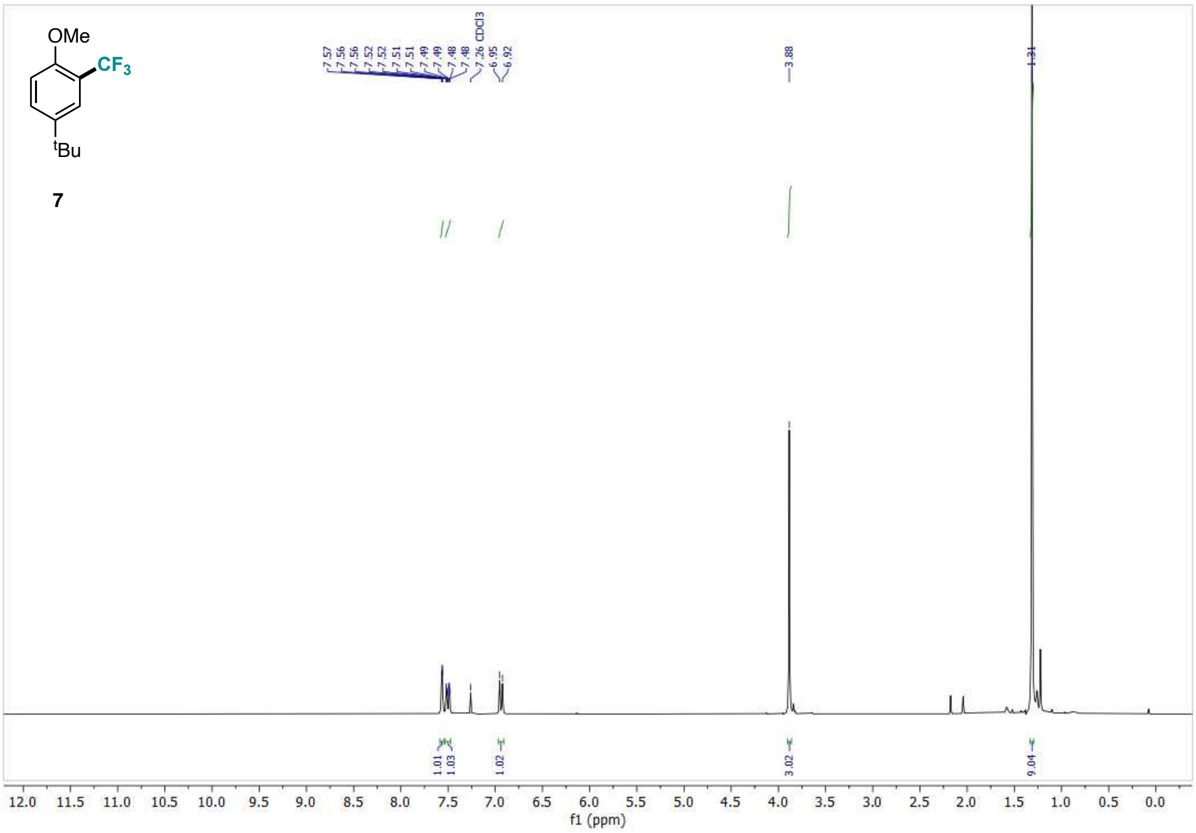

^{13}C-NMR (101 MHz, $CDCl_3$) of 7

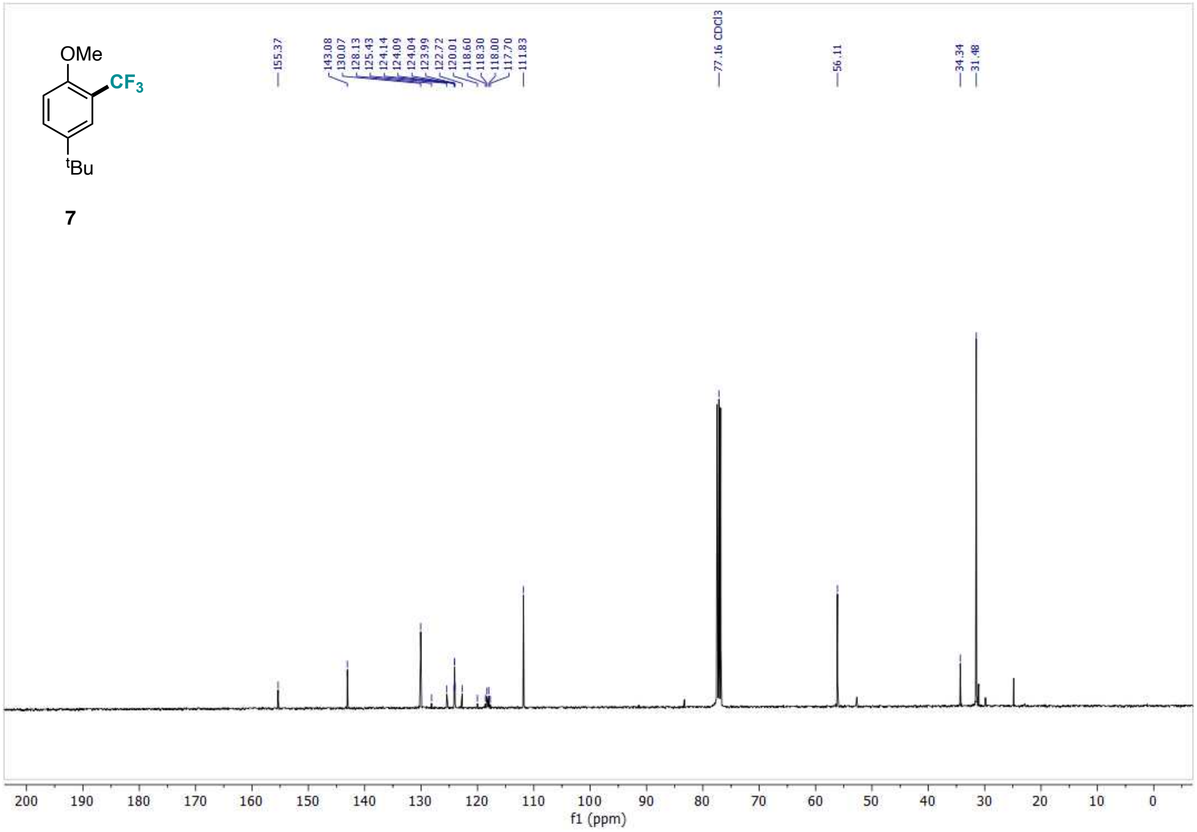

^{19}F-NMR (376 MHz, $CDCl_3$) of 7

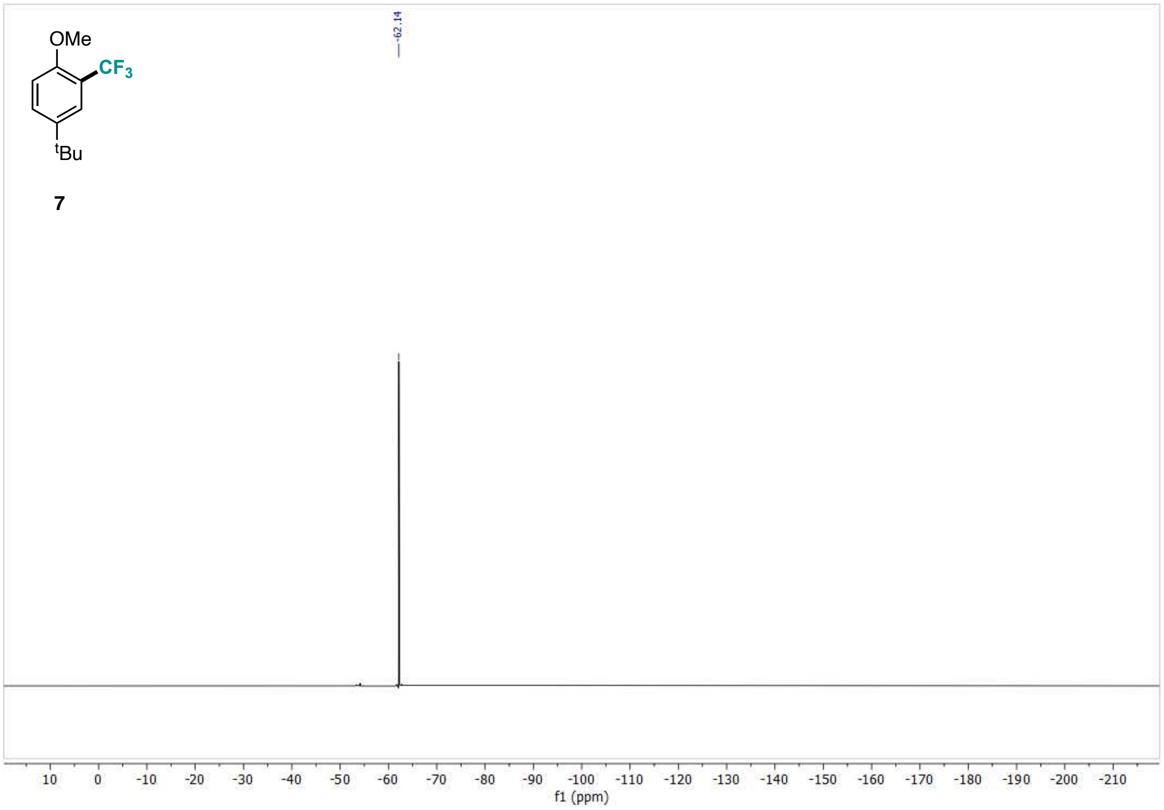

^{1}H-NMR (400 MHz, CDCl$_3$) of 8

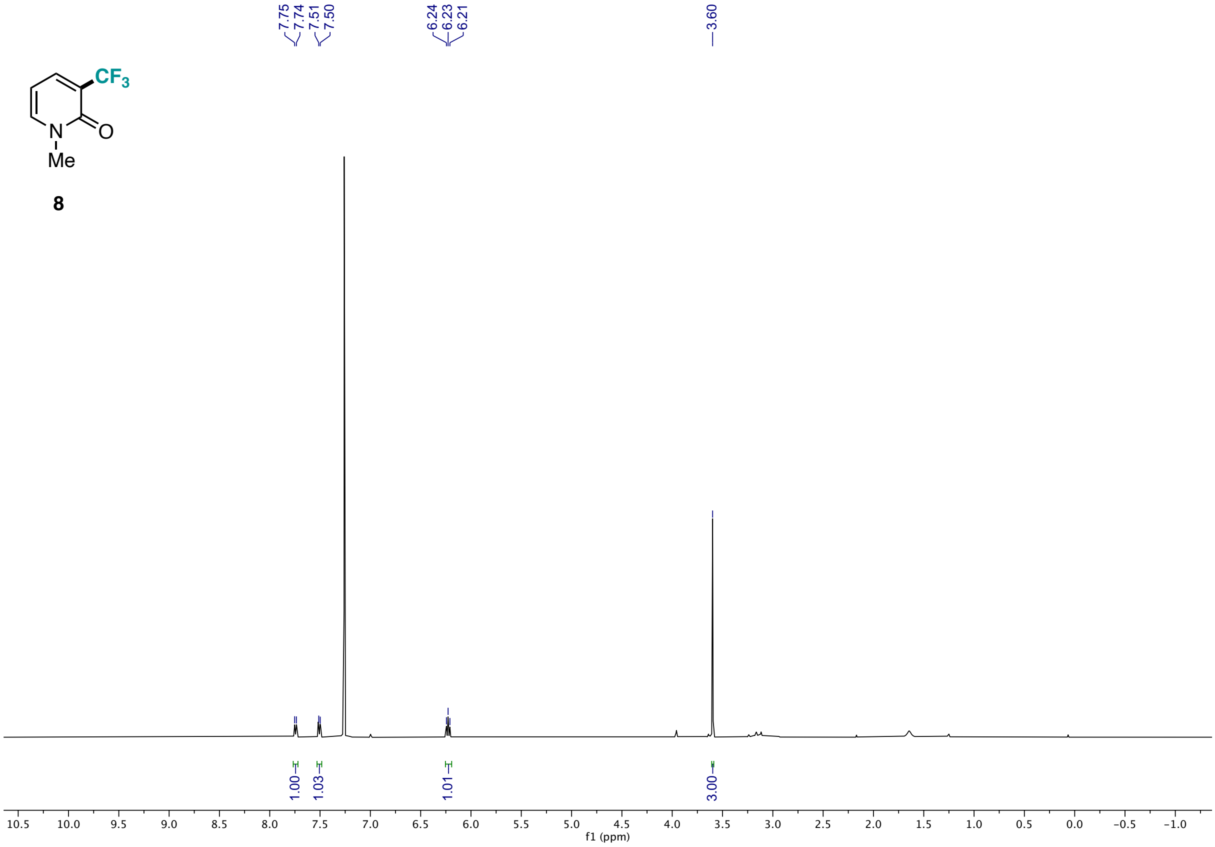

^{13}C-NMR (101 MHz, CDCl$_3$) of 8

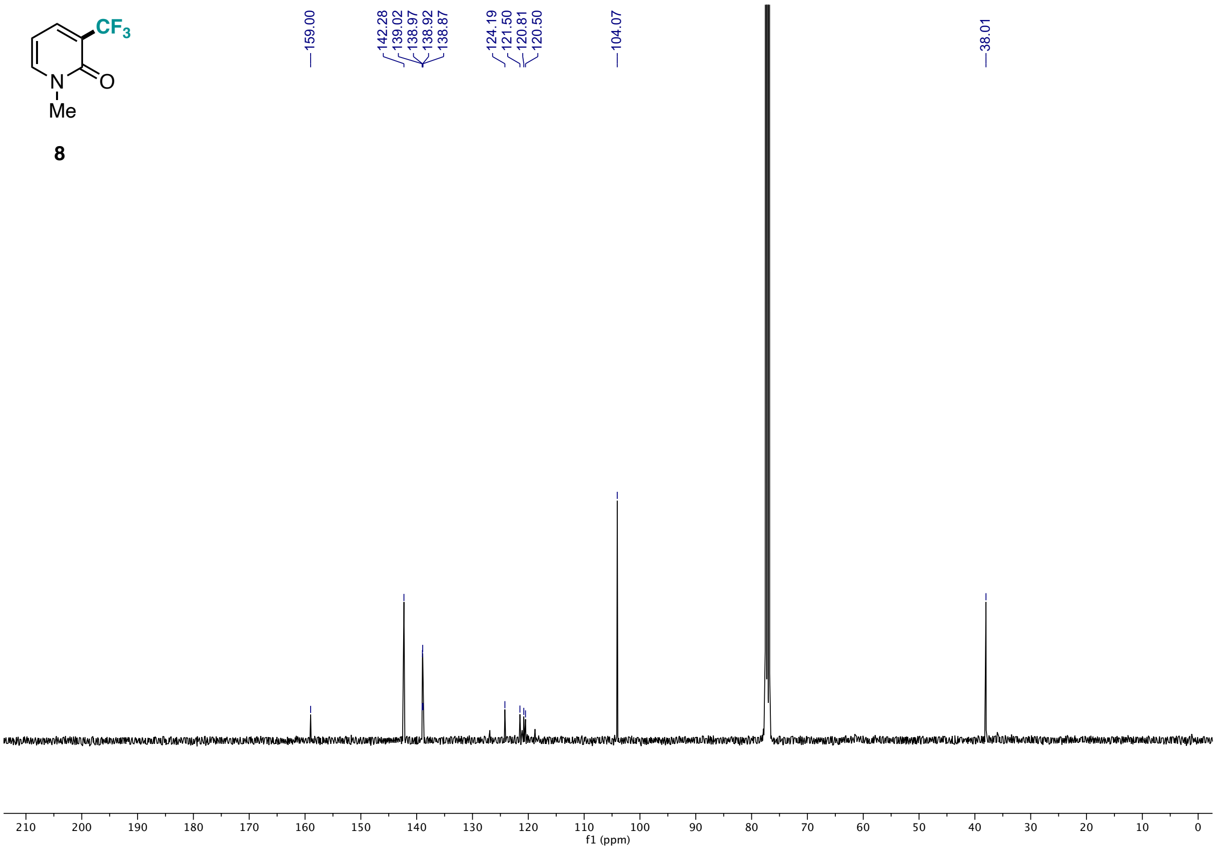

^{19}F-NMR (376 MHz, $CDCl_3$) of 8

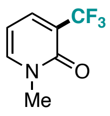

8

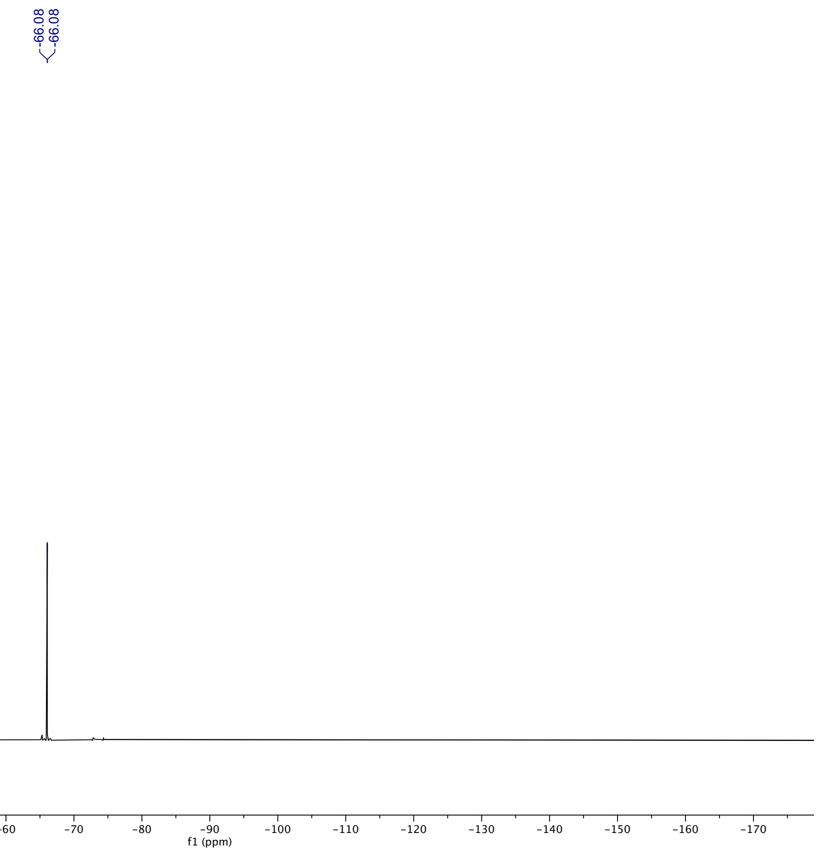

1H-NMR (400 MHz, $CDCl_3$) of 9

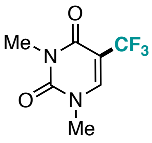

9

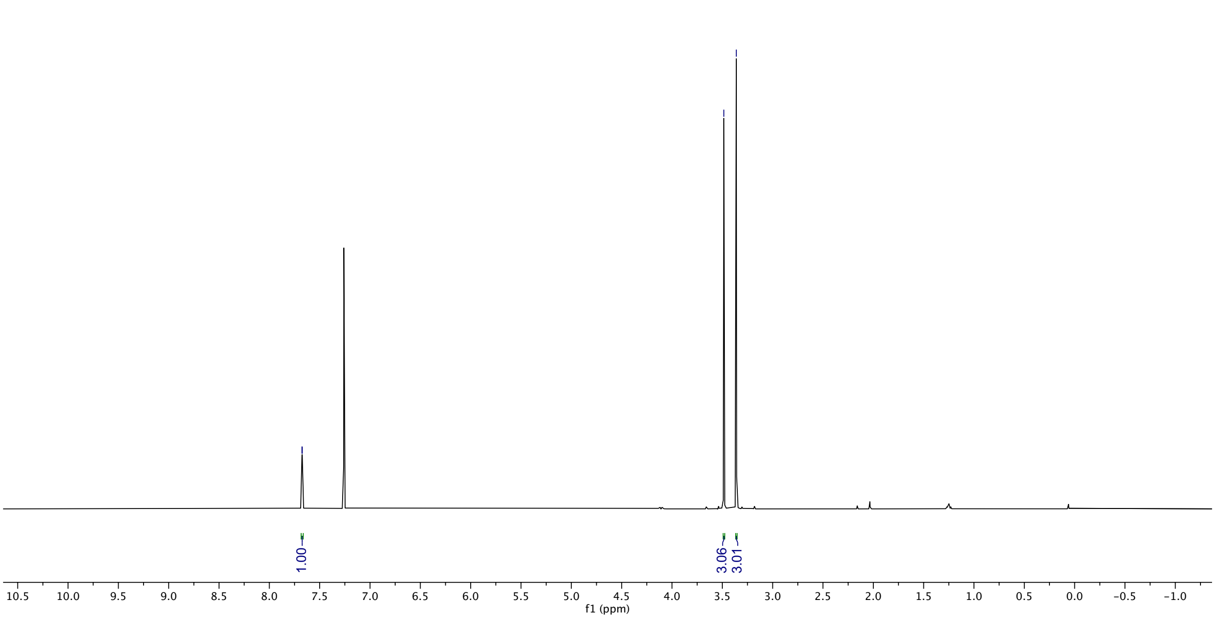

13C-NMR (101 MHz, $CDCl_3$) of 9

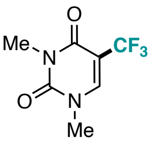

9

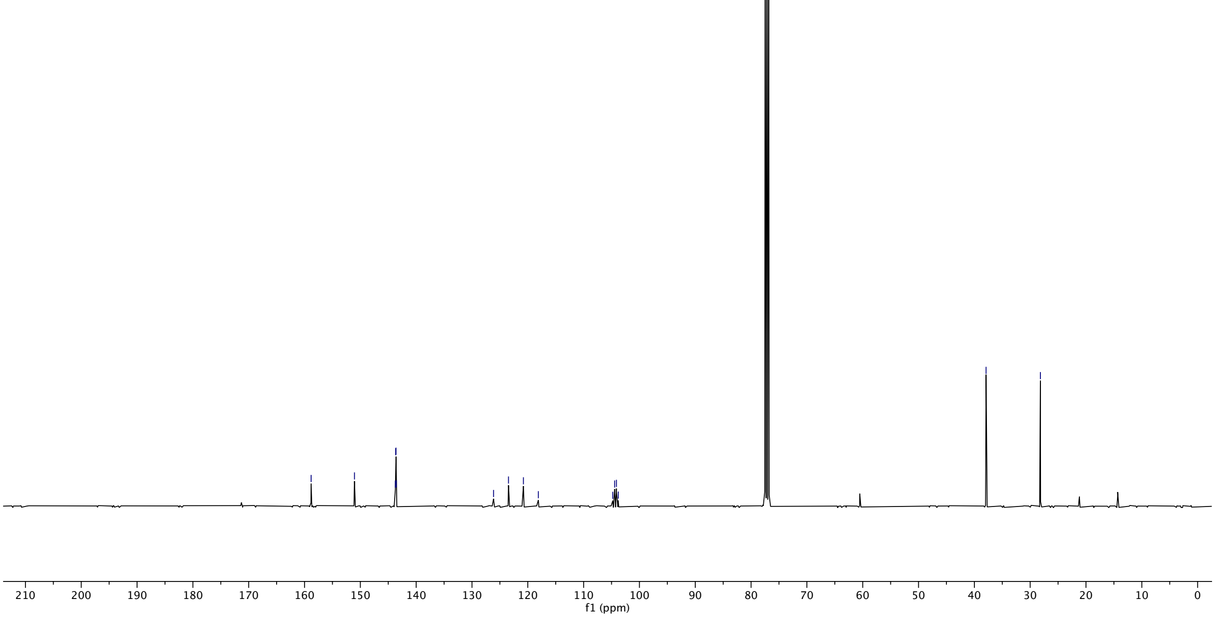

^{19}F-NMR (376 MHz, $CDCl_3$) of 9

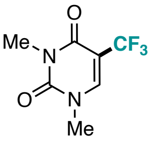

9

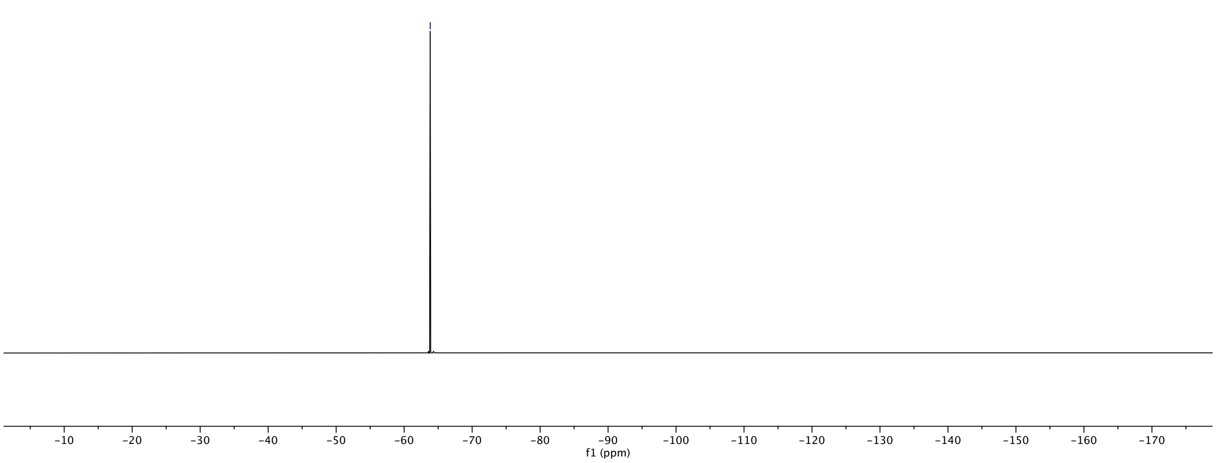

^{1}H-NMR (400 MHz, $CDCl_3$) of 10

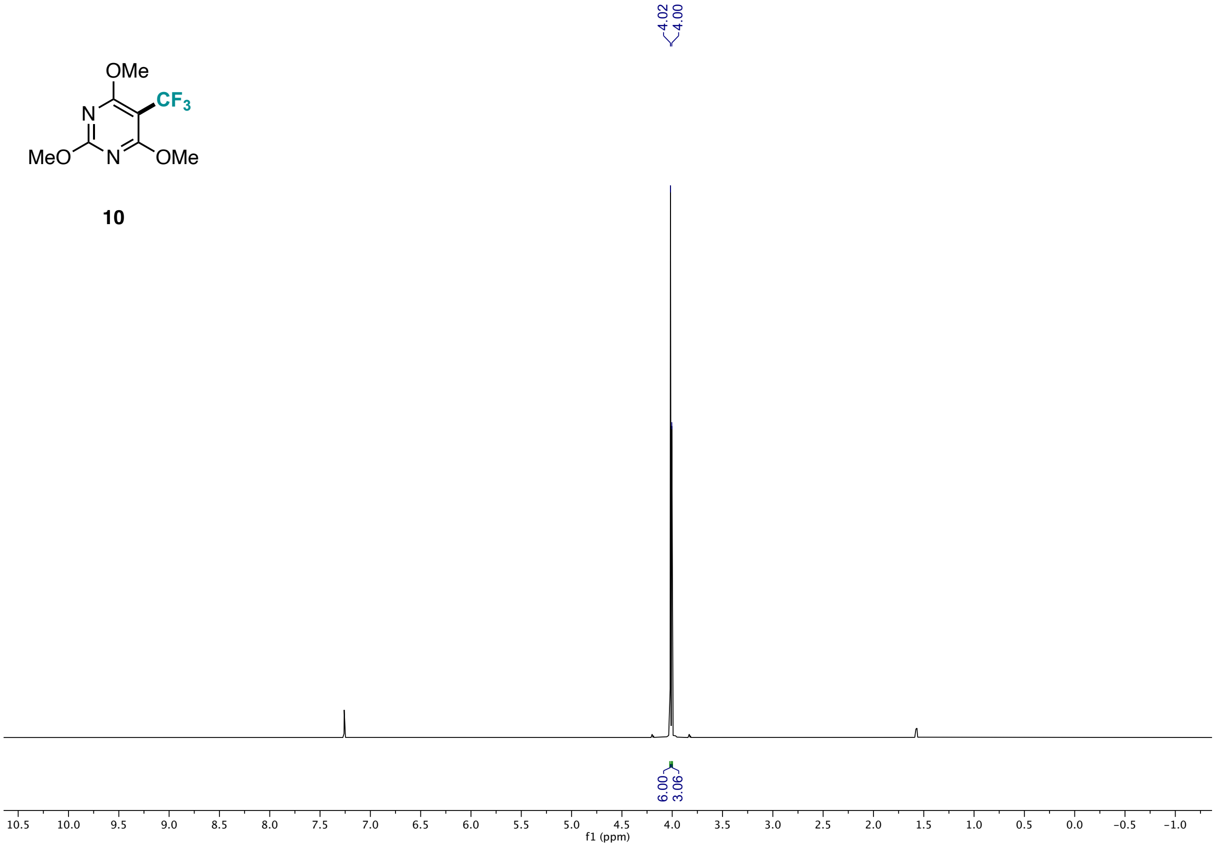

^{13}C-NMR (101 MHz, $CDCl_3$) of 10

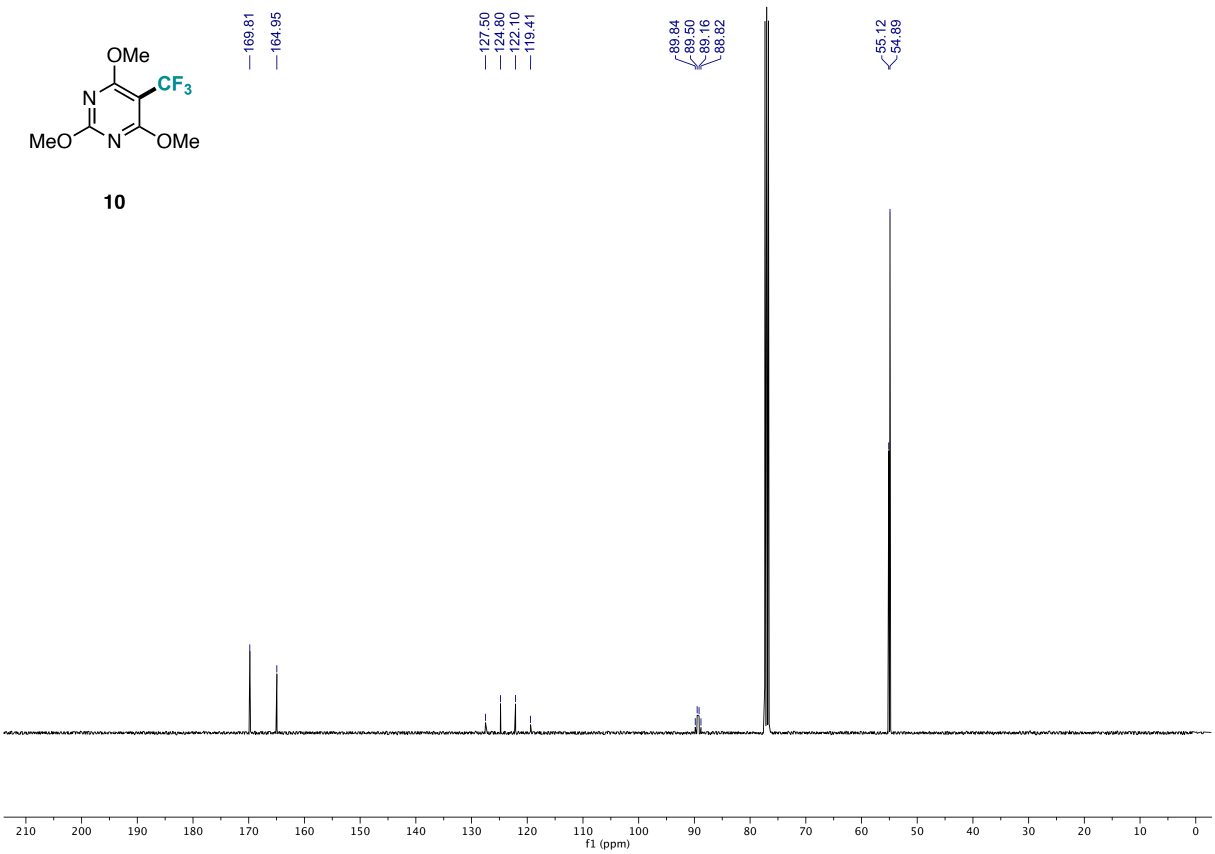

^{19}F-NMR (376 MHz, $CDCl_3$) of 10

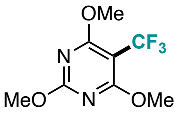

10

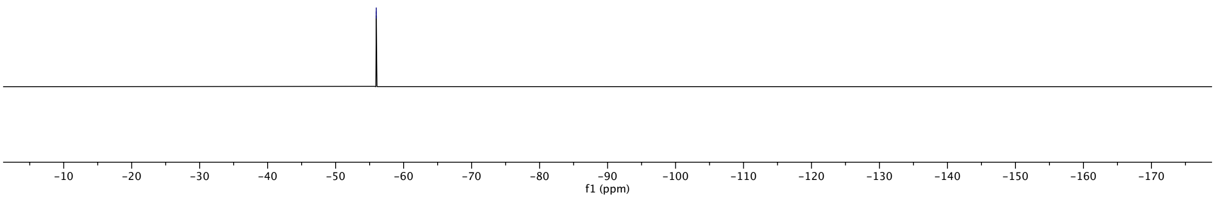

^{1}H-NMR (400 MHz, $CDCl_3$) of 11a + 11b

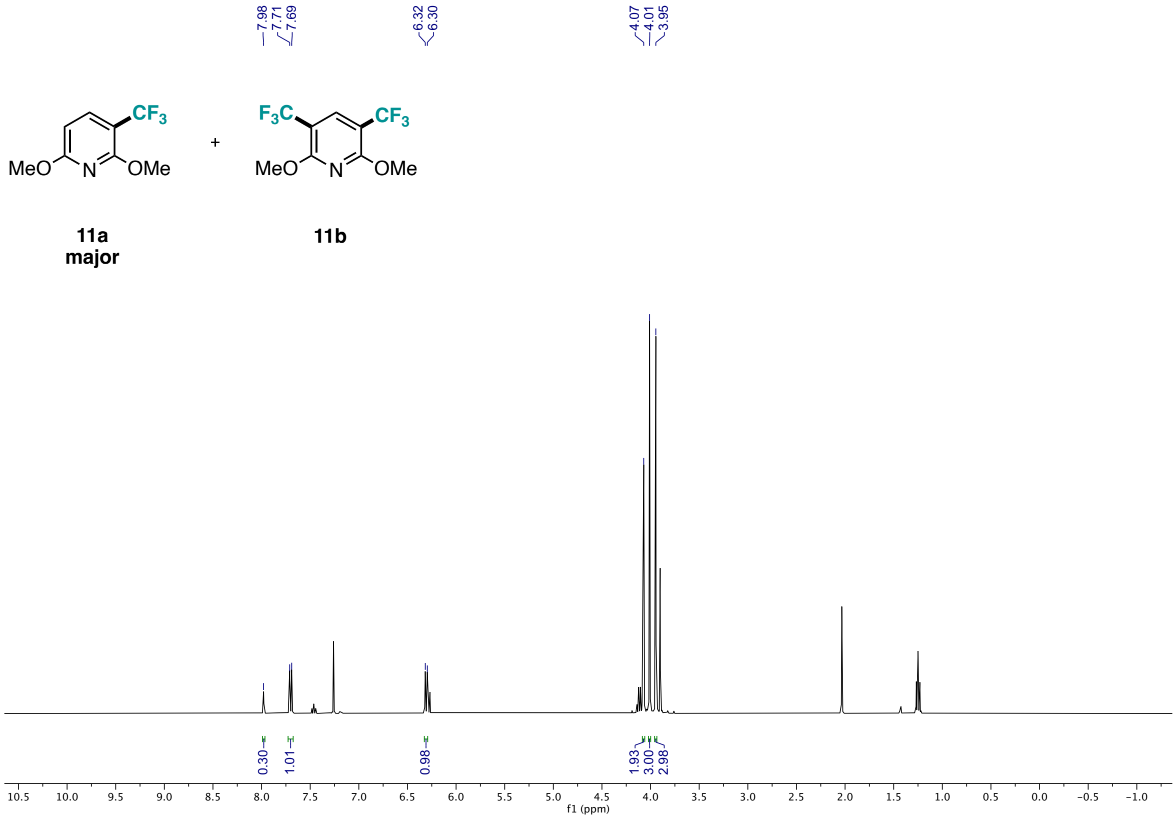

^{13}C-NMR (101 MHz, $CDCl_3$) of 11a + 11b

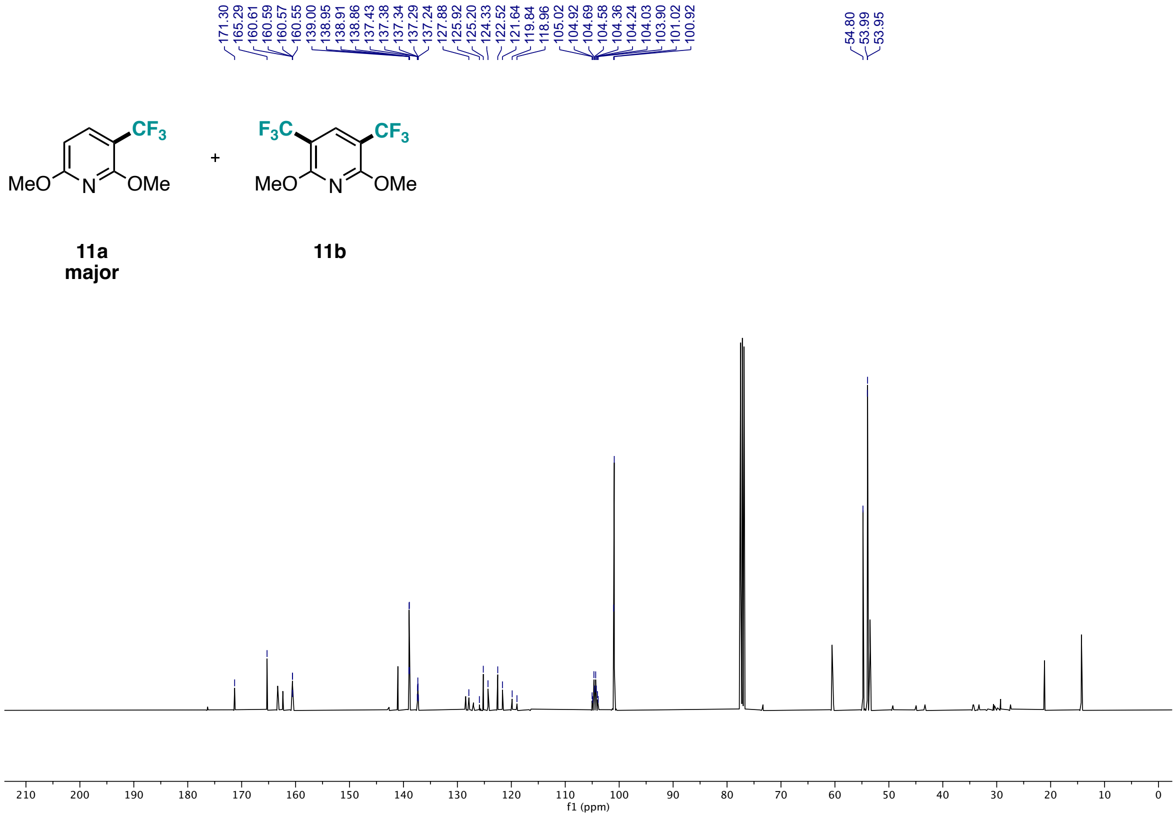

^{19}F-NMR (376 MHz, CDCl$_3$) of 11a + 11b

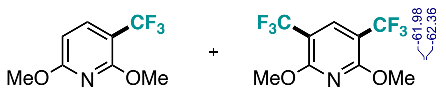

11a
major

11b

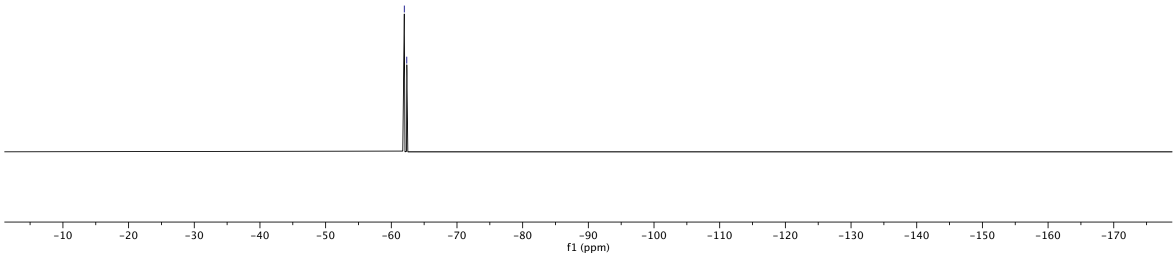

^{1}H-NMR (600 MHz, $CDCl_3$) of 12

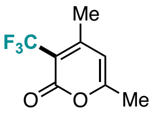

12

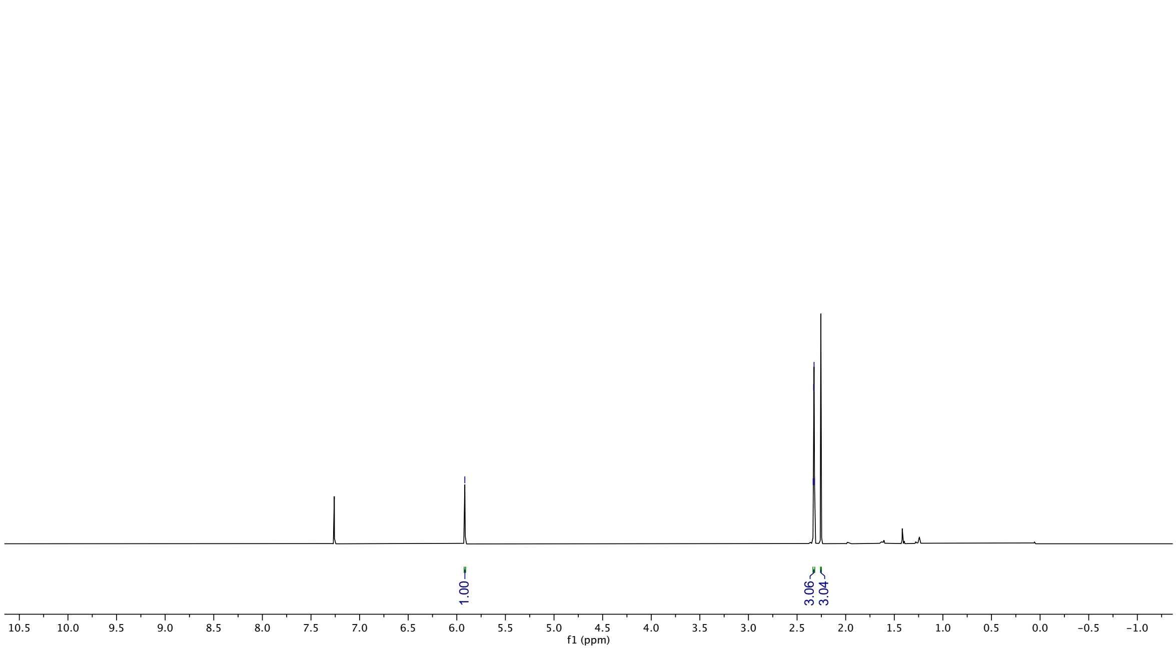

^{13}C-NMR (151 MHz, $CDCl_3$) of 12

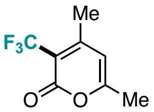

12

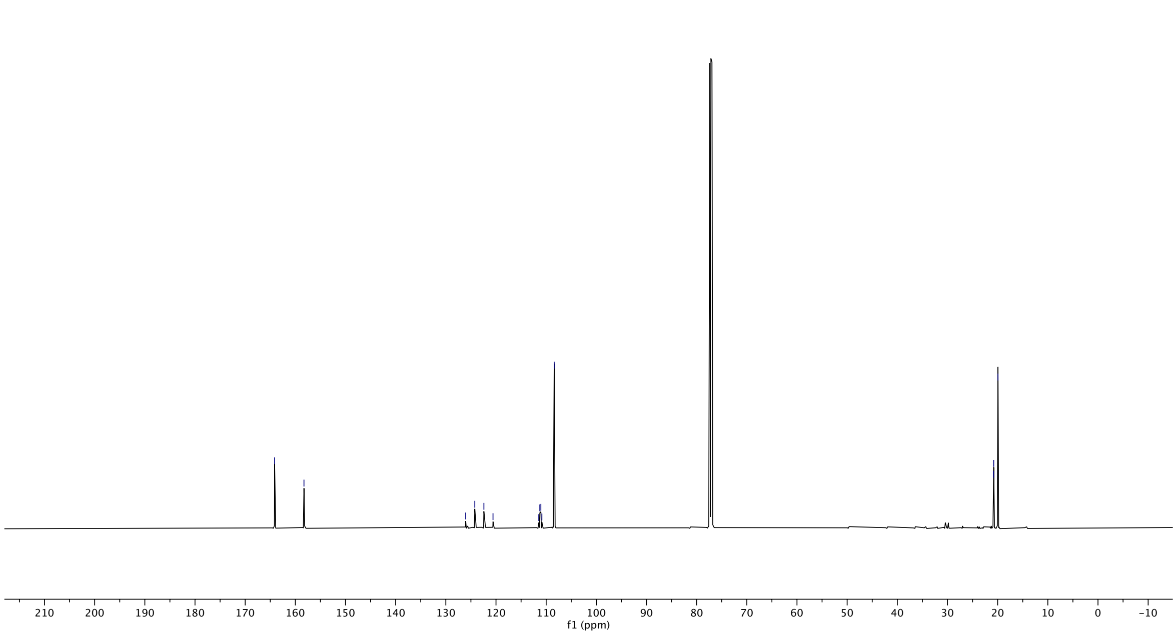

^{19}F-NMR (376 MHz, $CDCl_3$) of 12

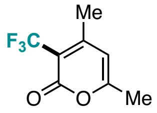

12

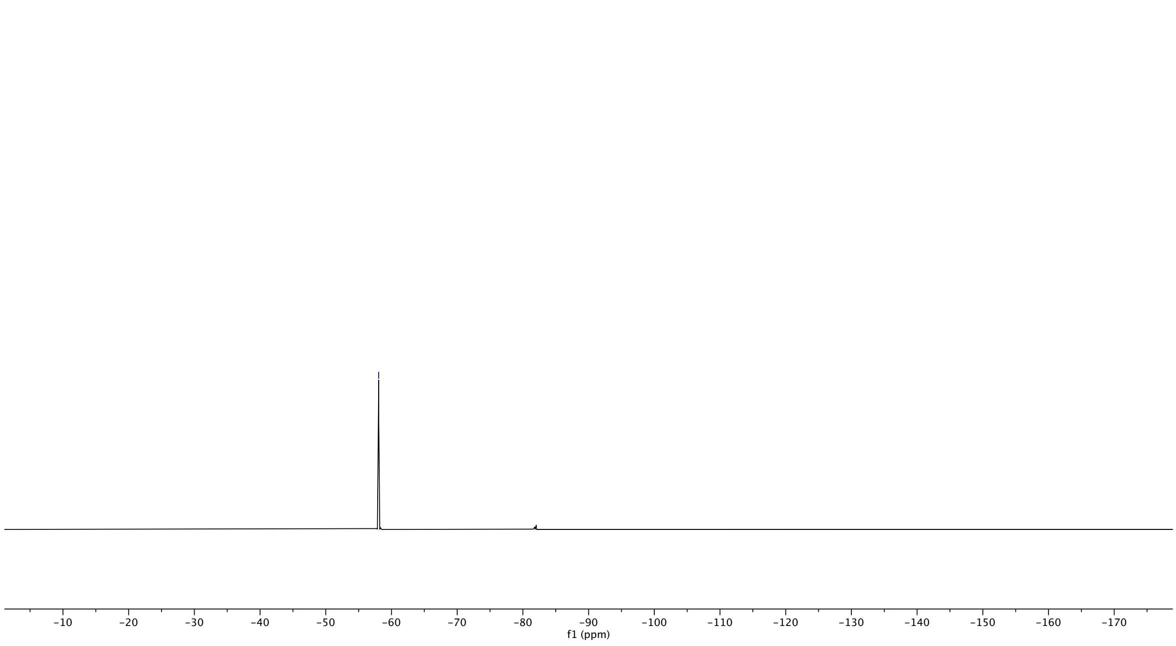

^{1}H-NMR (400 MHz, $CDCl_3$) of 13

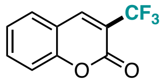

13

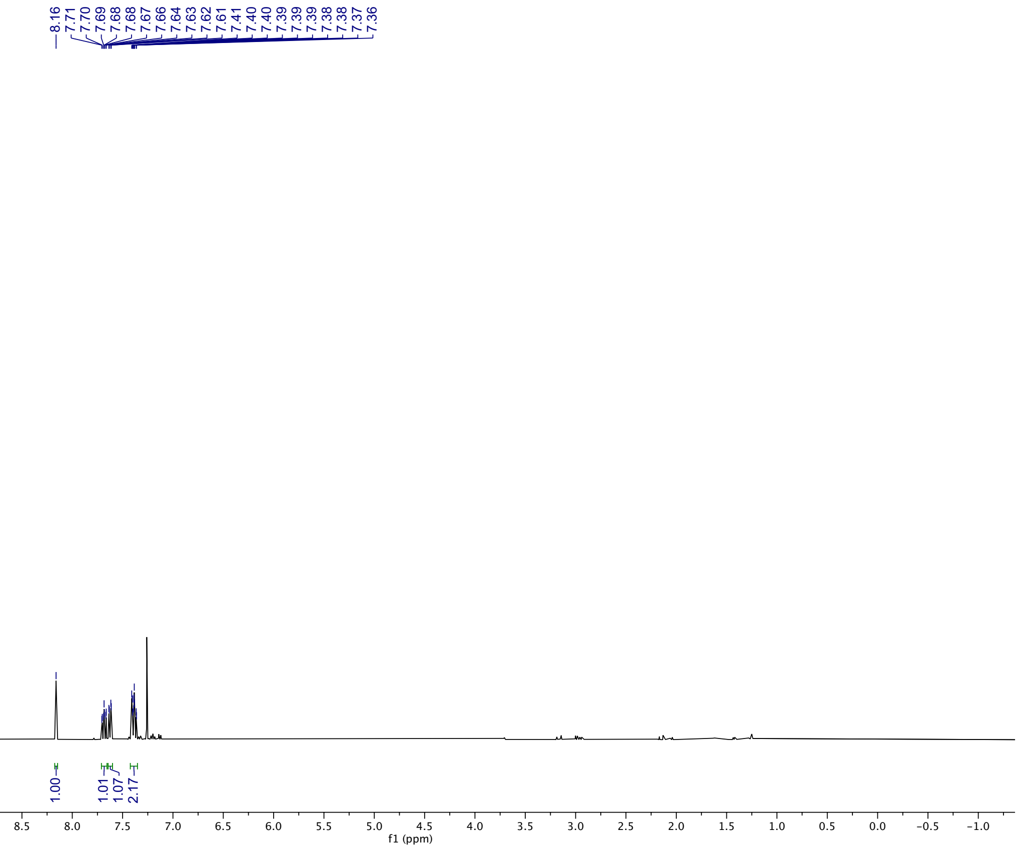

^{13}C-NMR (101 MHz, $CDCl_3$) of 13

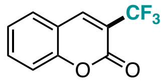

13

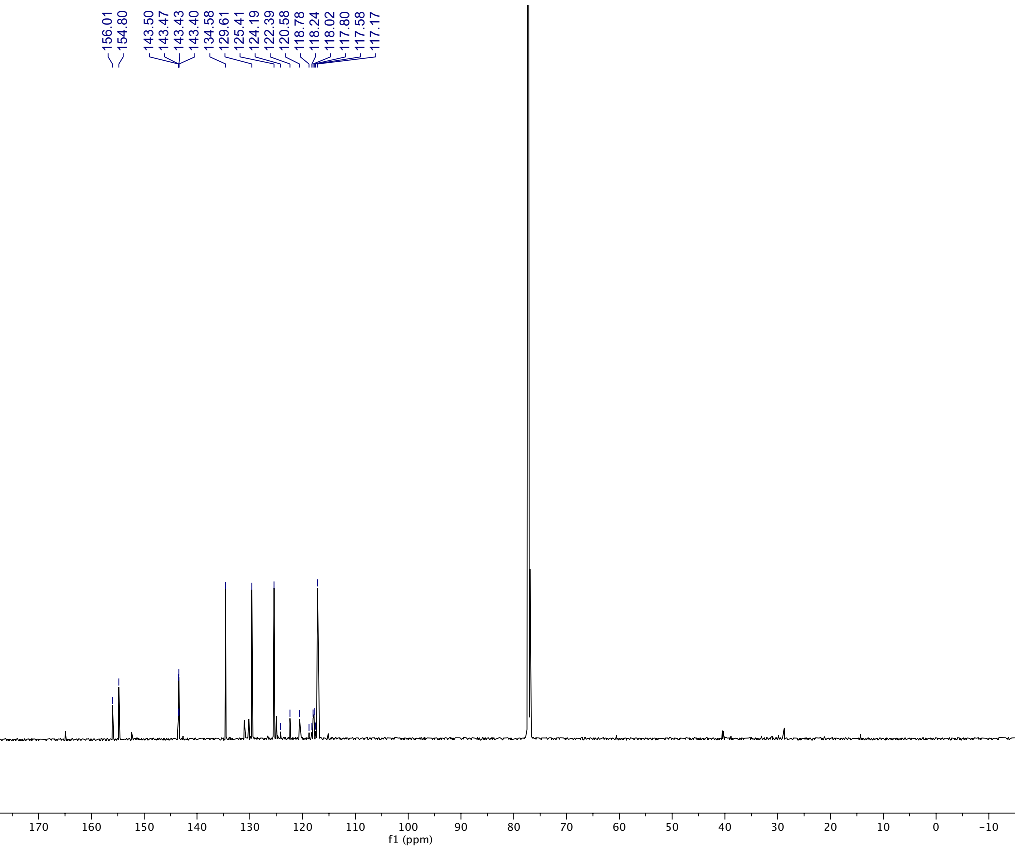

^{19}F-NMR (376 MHz, $CDCl_3$) of 13

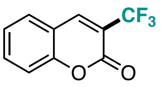

13

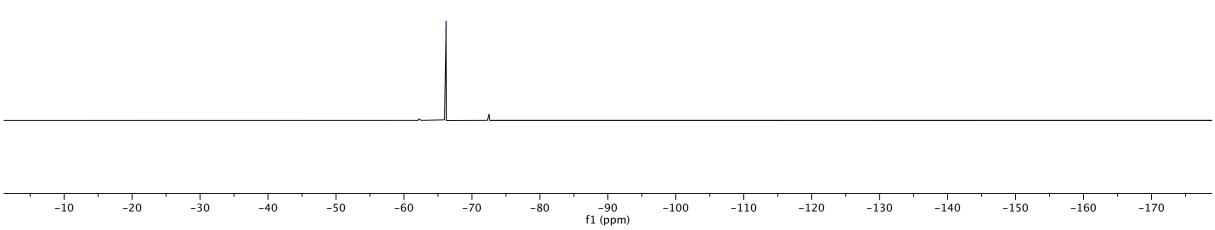

^{1}H-NMR (400 MHz, $CDCl_3$) of 14

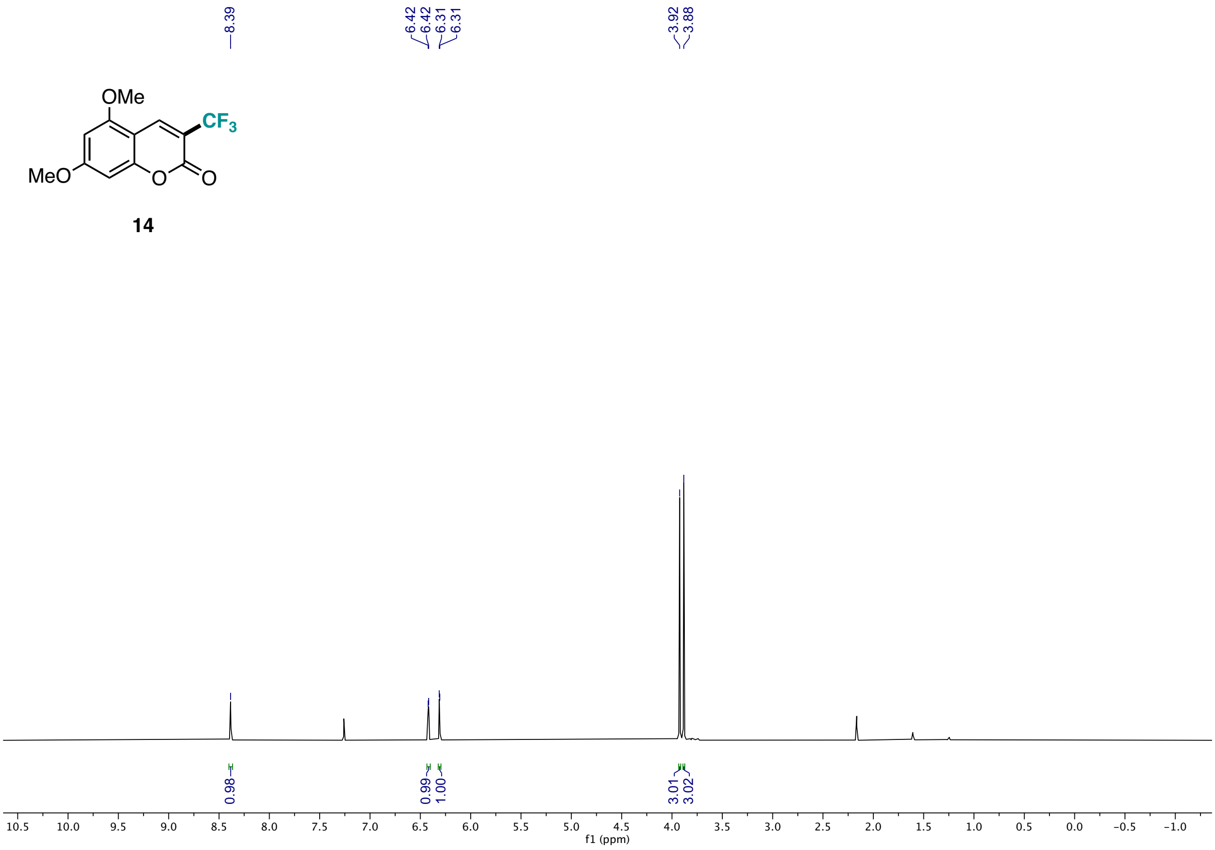

^{13}C-NMR (101 MHz, $CDCl_3$) of 14

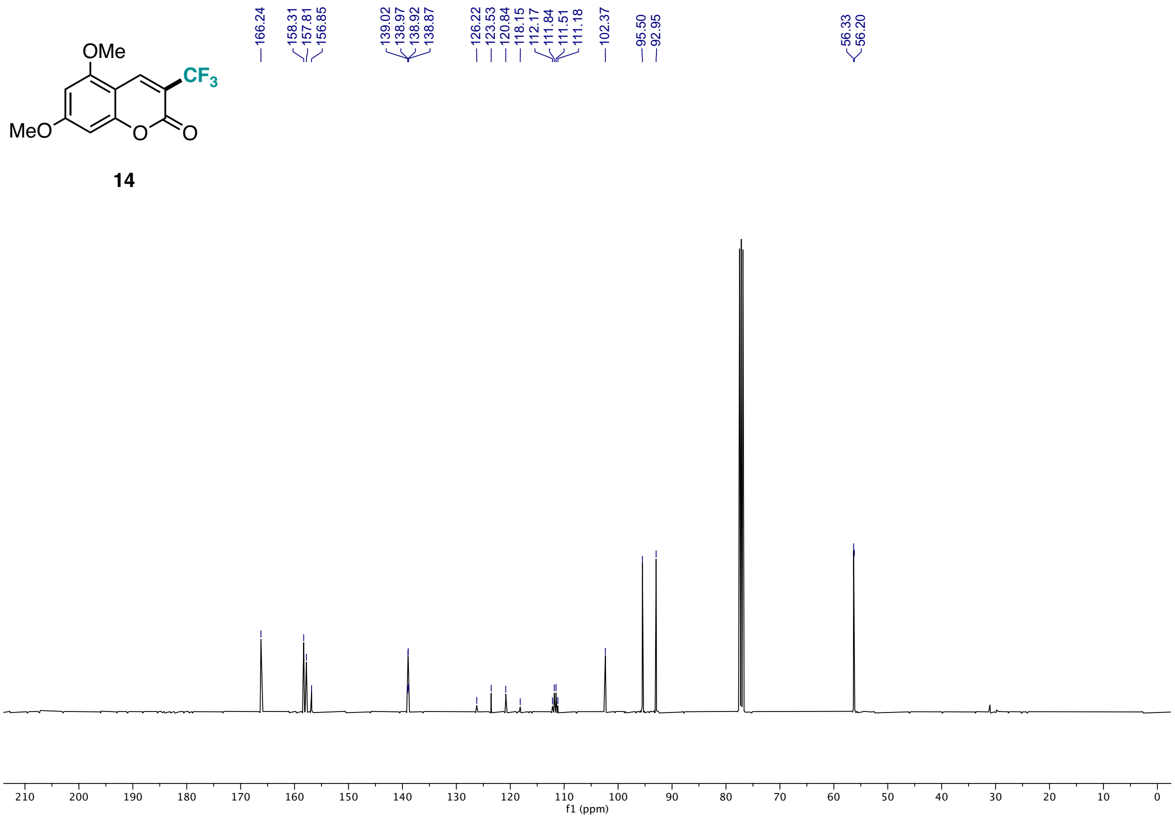

^{19}F-NMR (376 MHz, $CDCl_3$) of 14

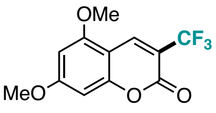

14

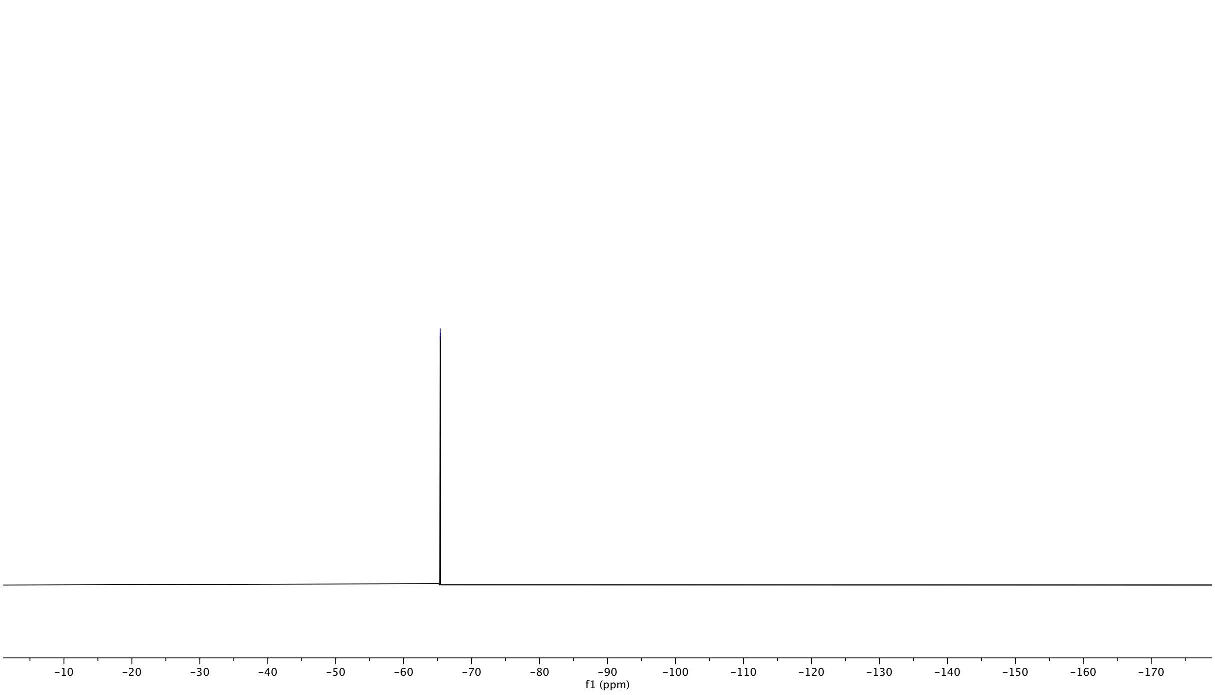

^{1}H-NMR (400 MHz, $CDCl_3$) of 15a + 15b

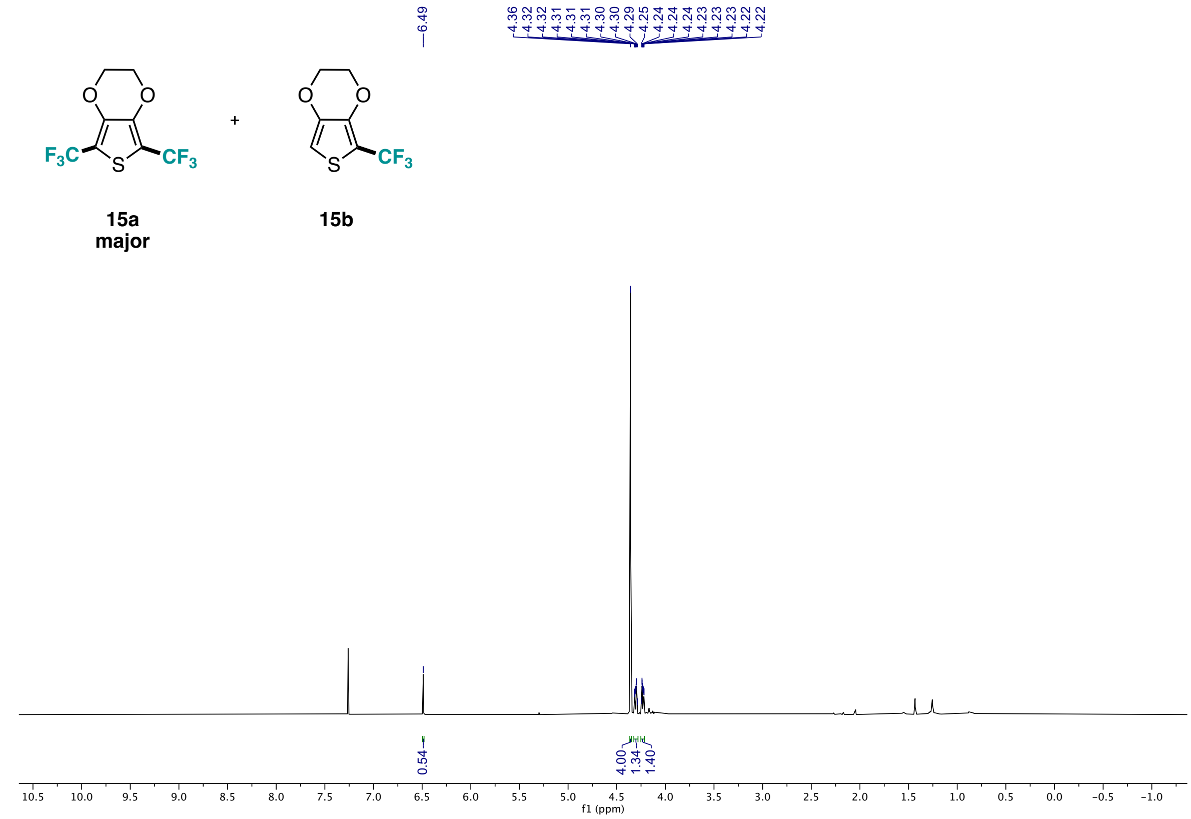

^{13}C-NMR (101 MHz, $CDCl_3$) of 15a + 15b

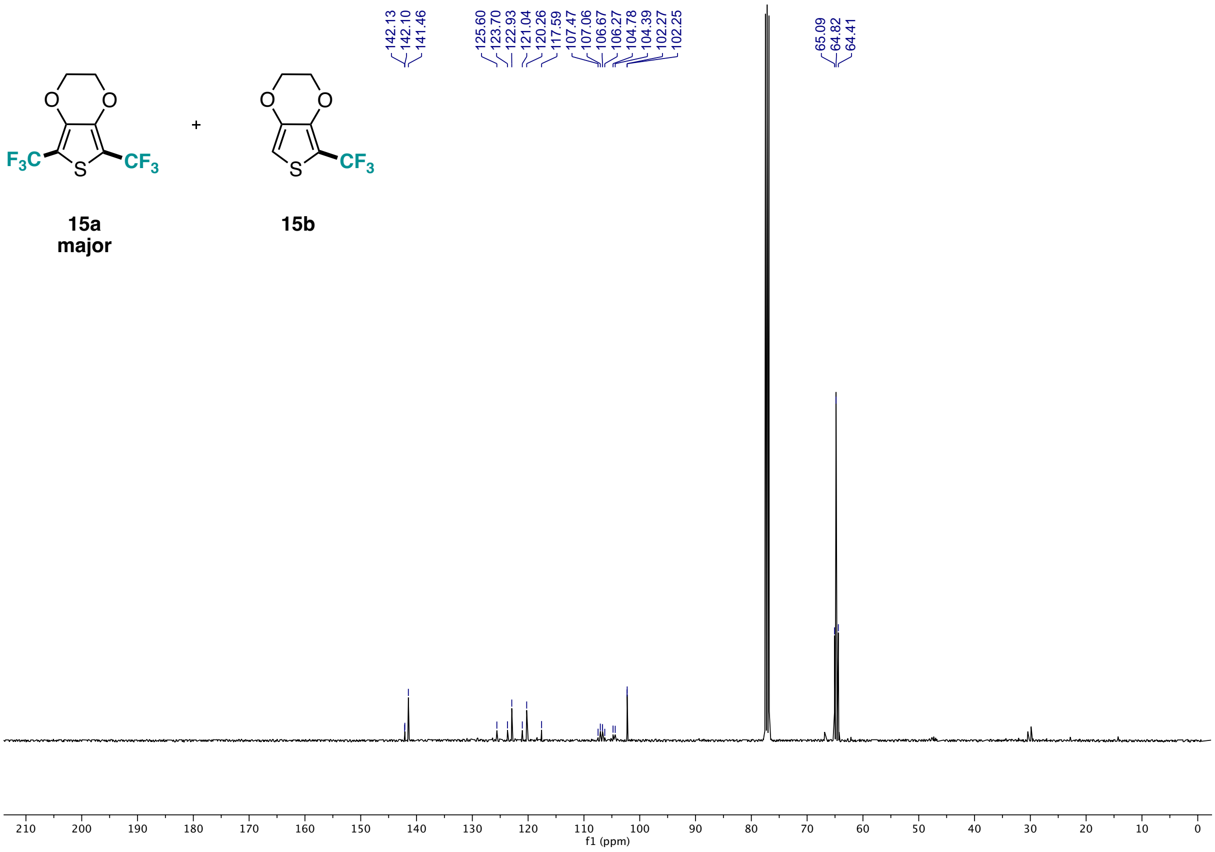

^{19}F-NMR (376 MHz, $CDCl_3$) of 15a + 15b

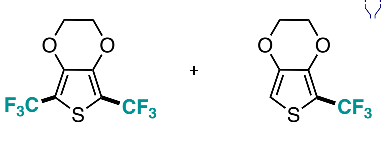

15a
major

15b

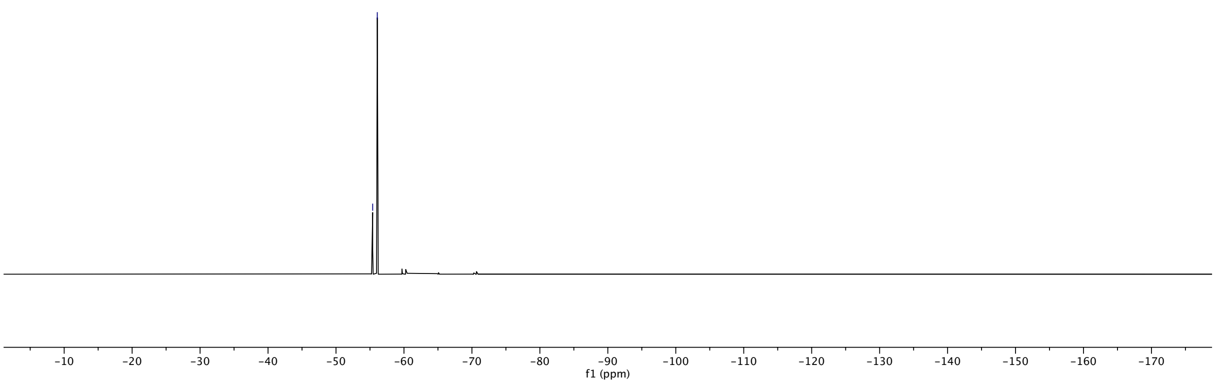

^{1}H-NMR (400 MHz, $CDCl_3$) of 16

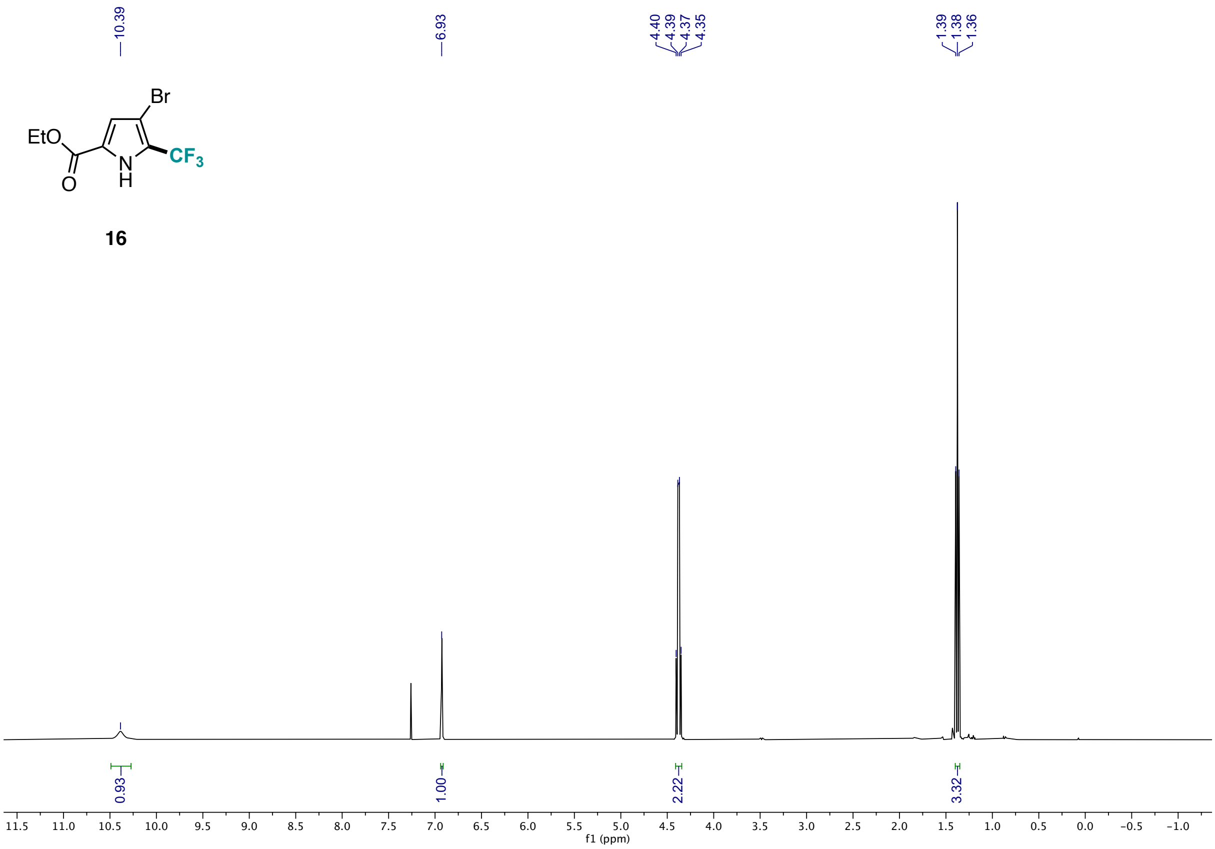

^{13}C-NMR (101 MHz, $CDCl_3$) of 16

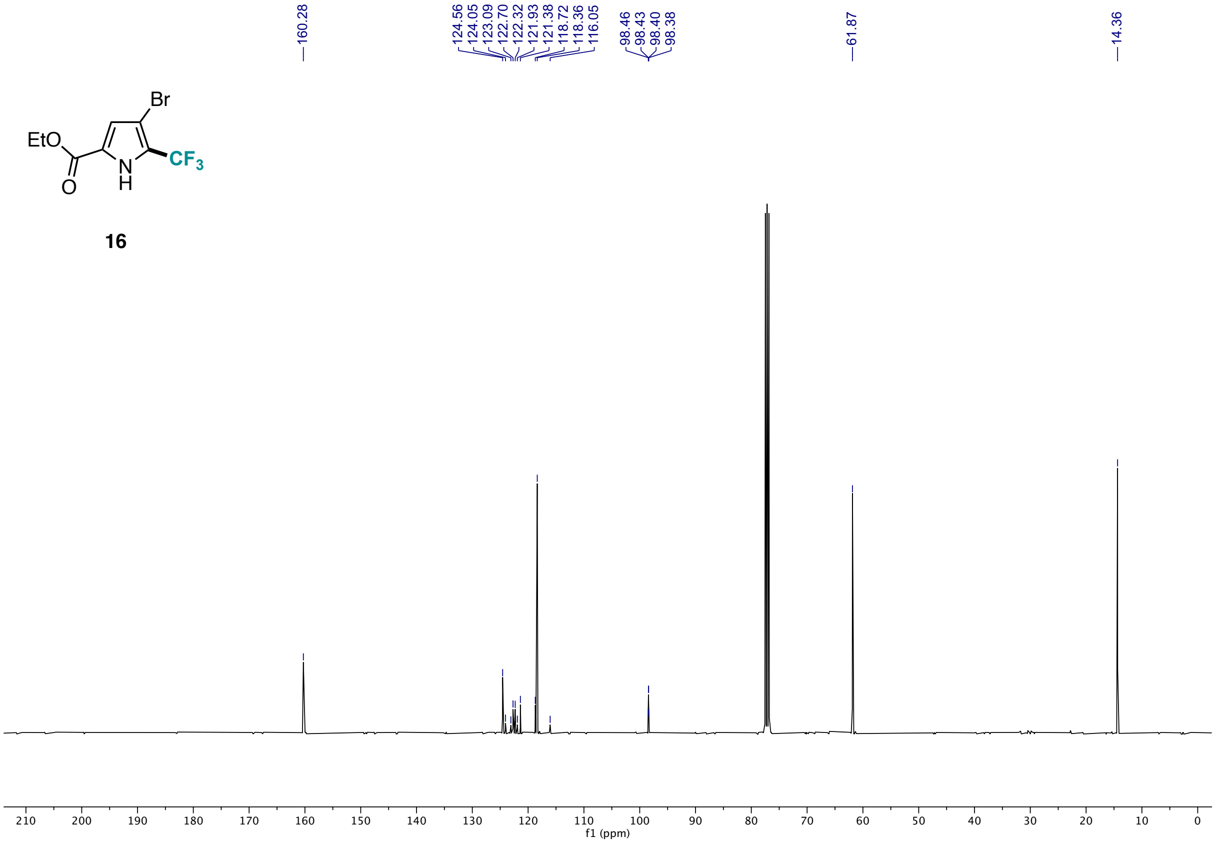

^{19}F-NMR (376 MHz, $CDCl_3$) of 16

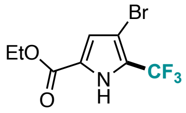

16

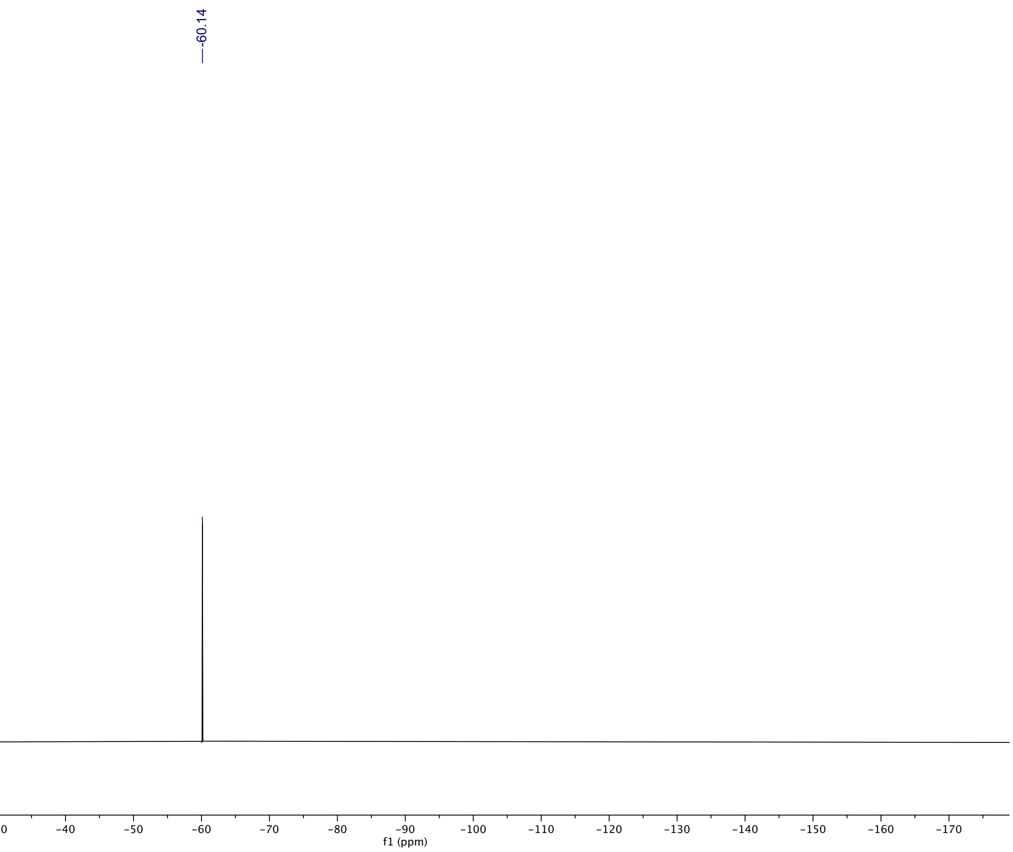

^{1}H-NMR (400 MHz, $CDCl_3$) of 17

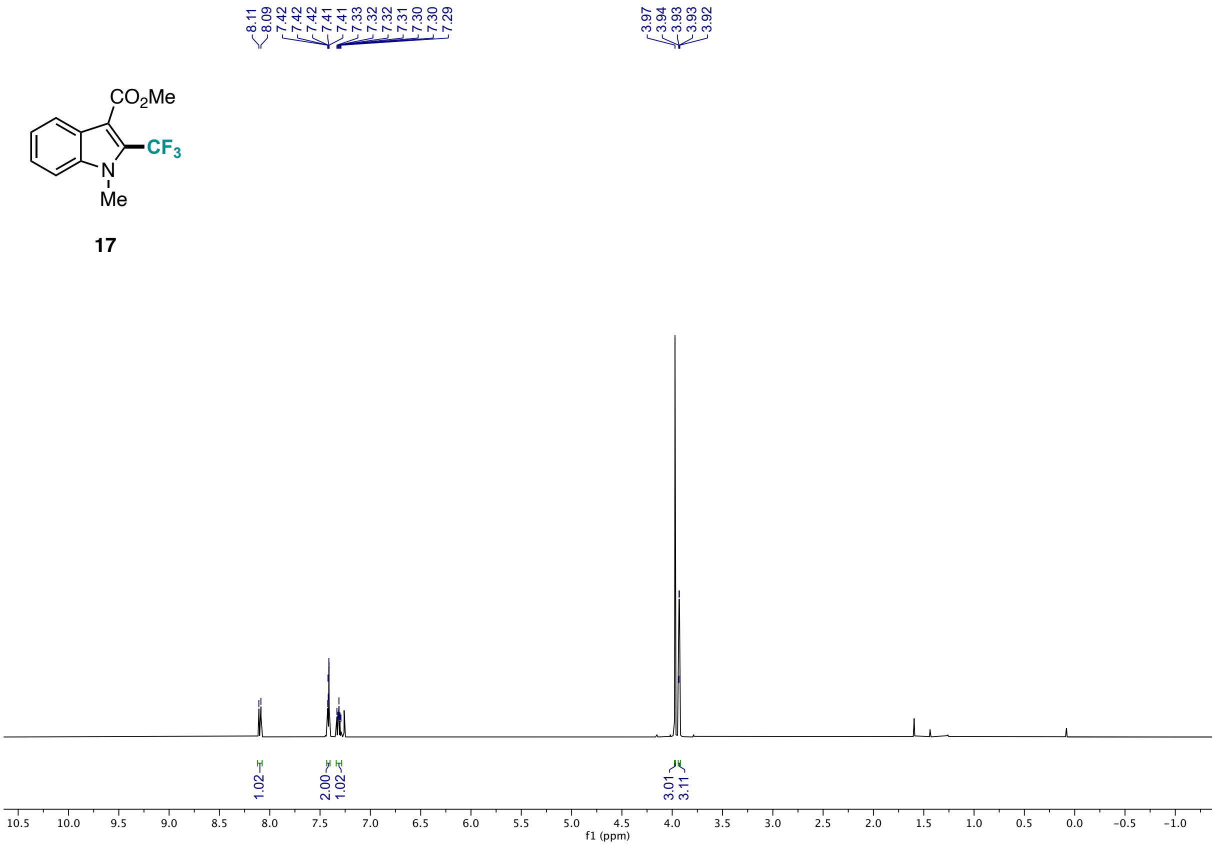

17

^{13}C-NMR (101 MHz, $CDCl_3$) of 17

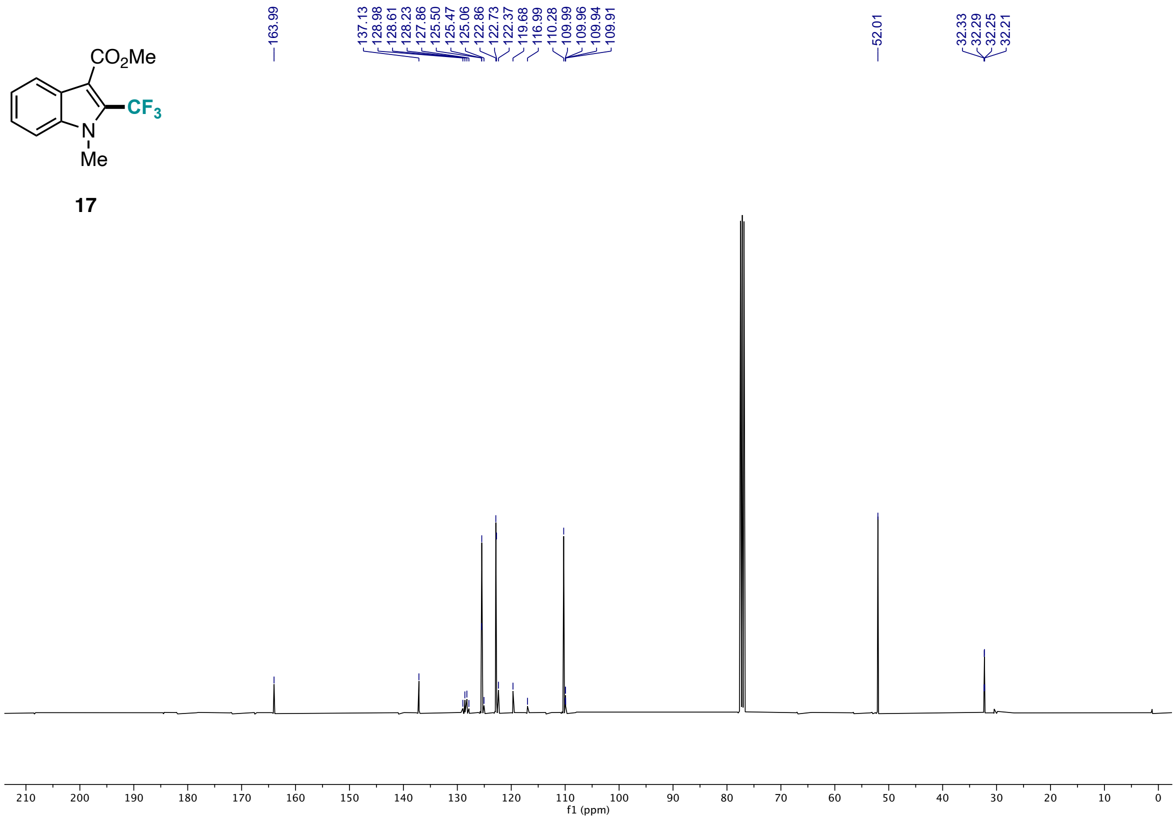

17

^{19}F-NMR (376 MHz, $CDCl_3$) of 17

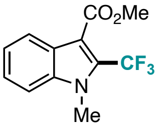

17

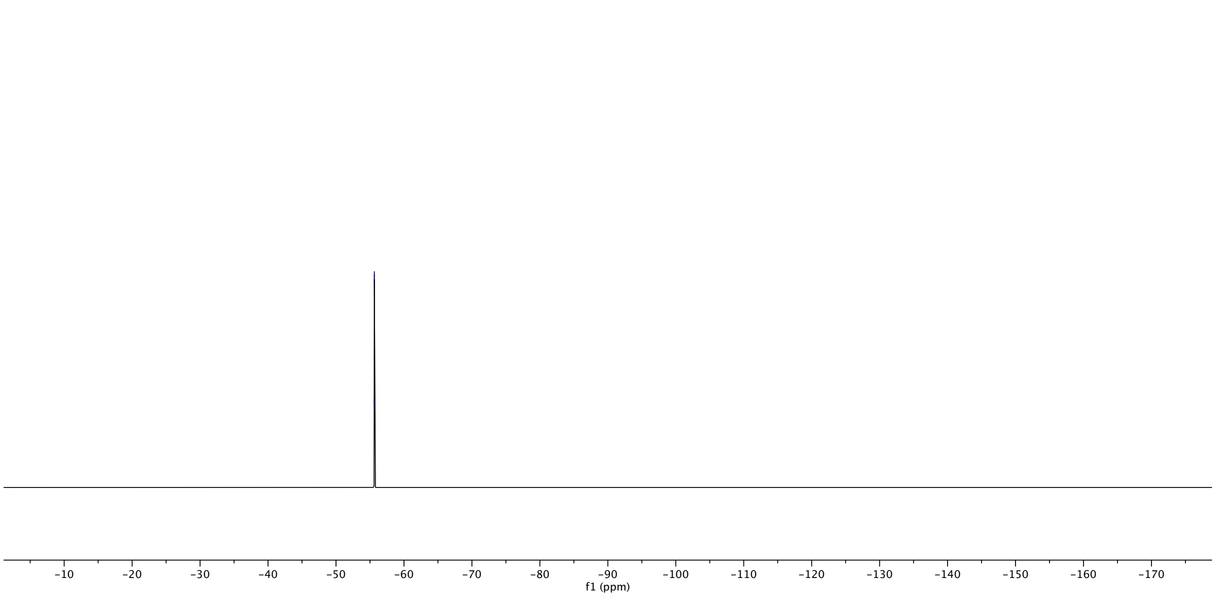

^{1}H-NMR (300 MHz, $CDCl_3$) of 18

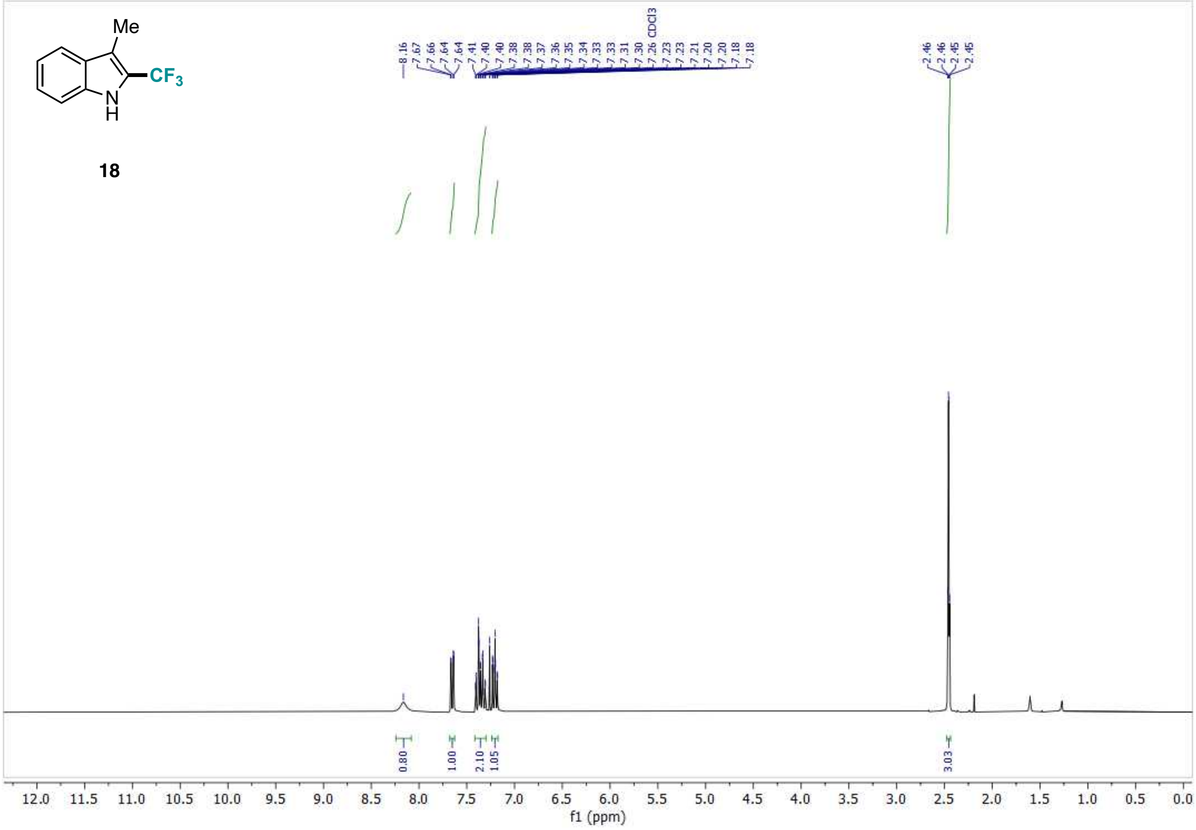

^{13}C-NMR (101 MHz, $CDCl_3$) of 18

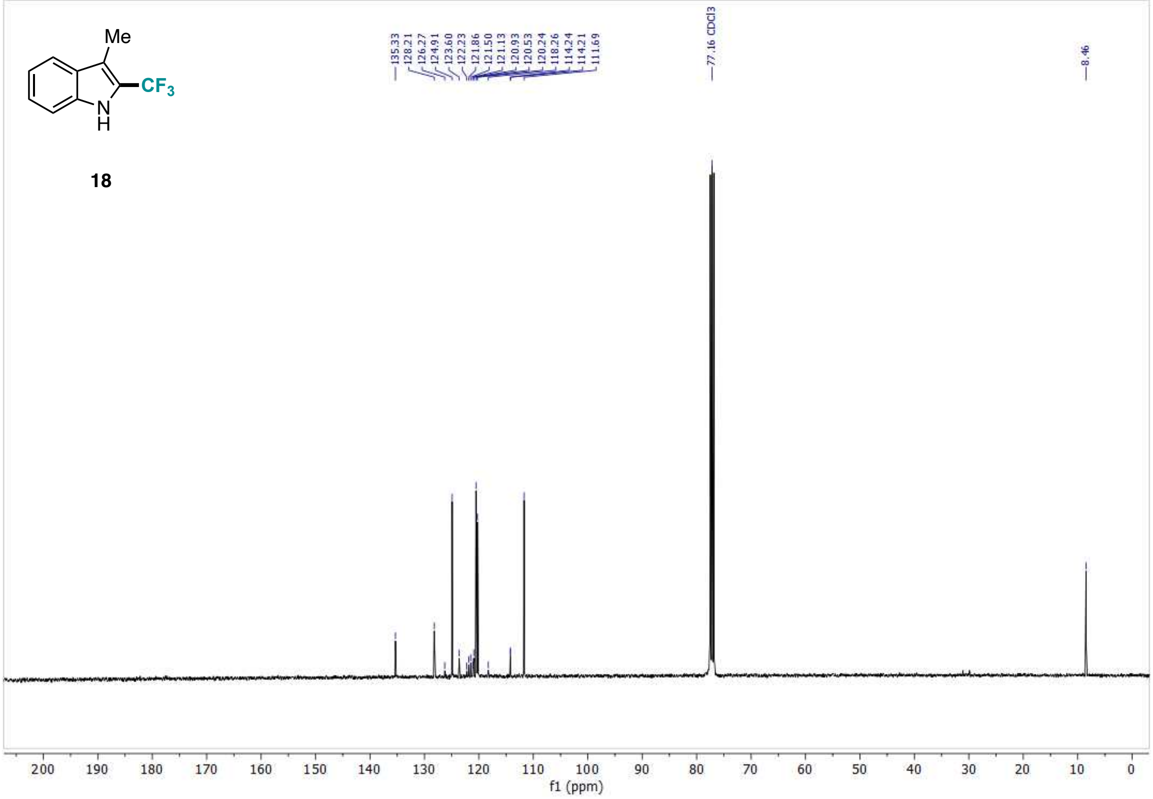

^{19}F-NMR (376 MHz, $CDCl_3$) of 18

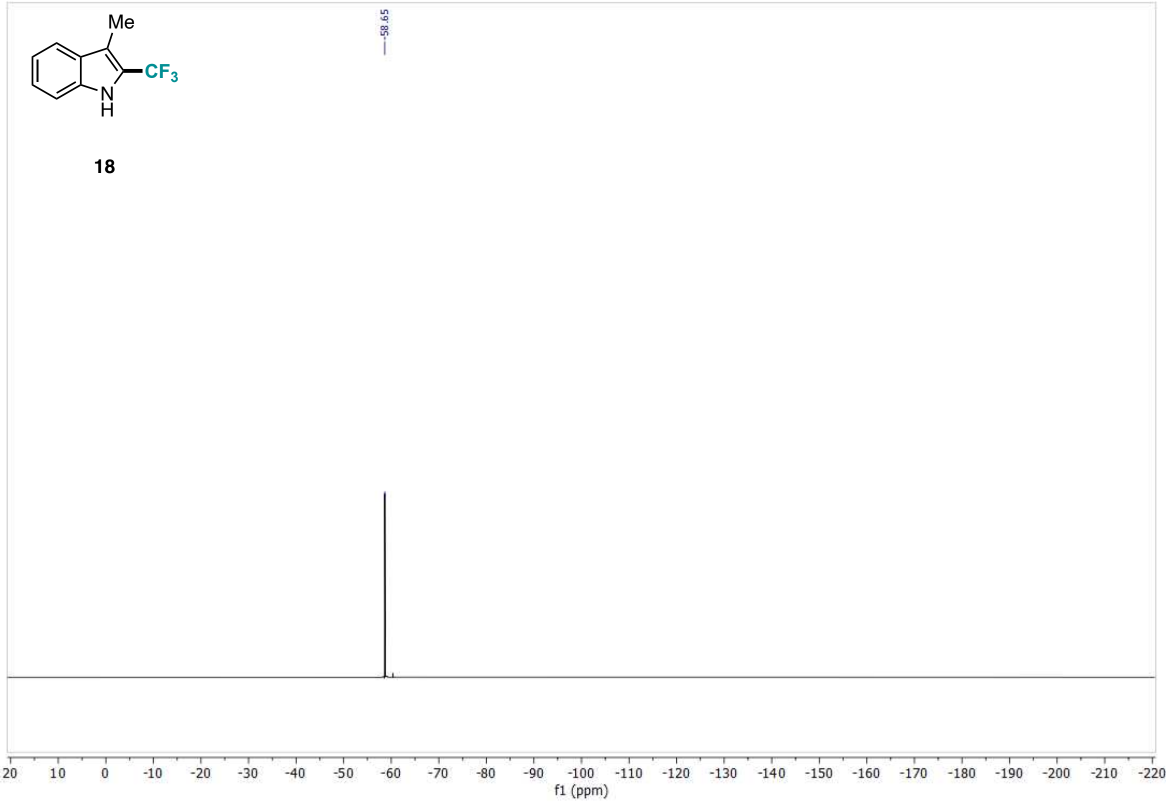

^{1}H-NMR (400 MHz, $CDCl_3$) of 19

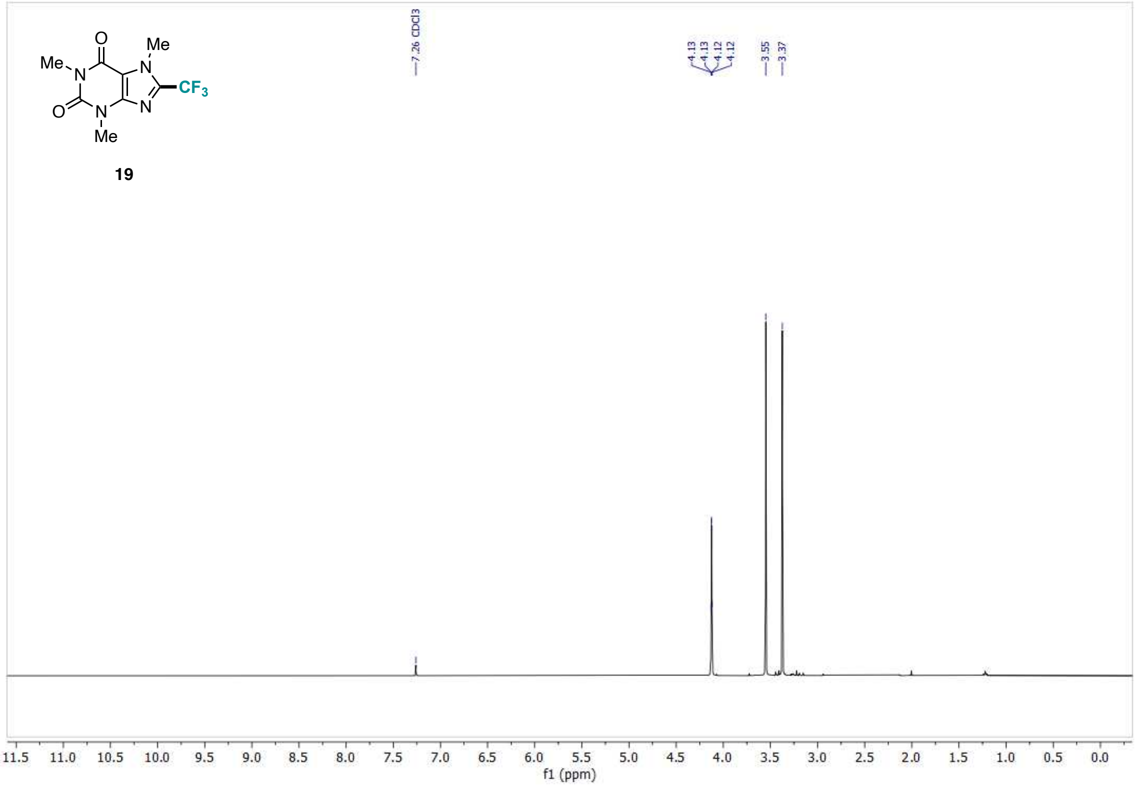

^{13}C-NMR (101 MHz, $CDCl_3$) of 19

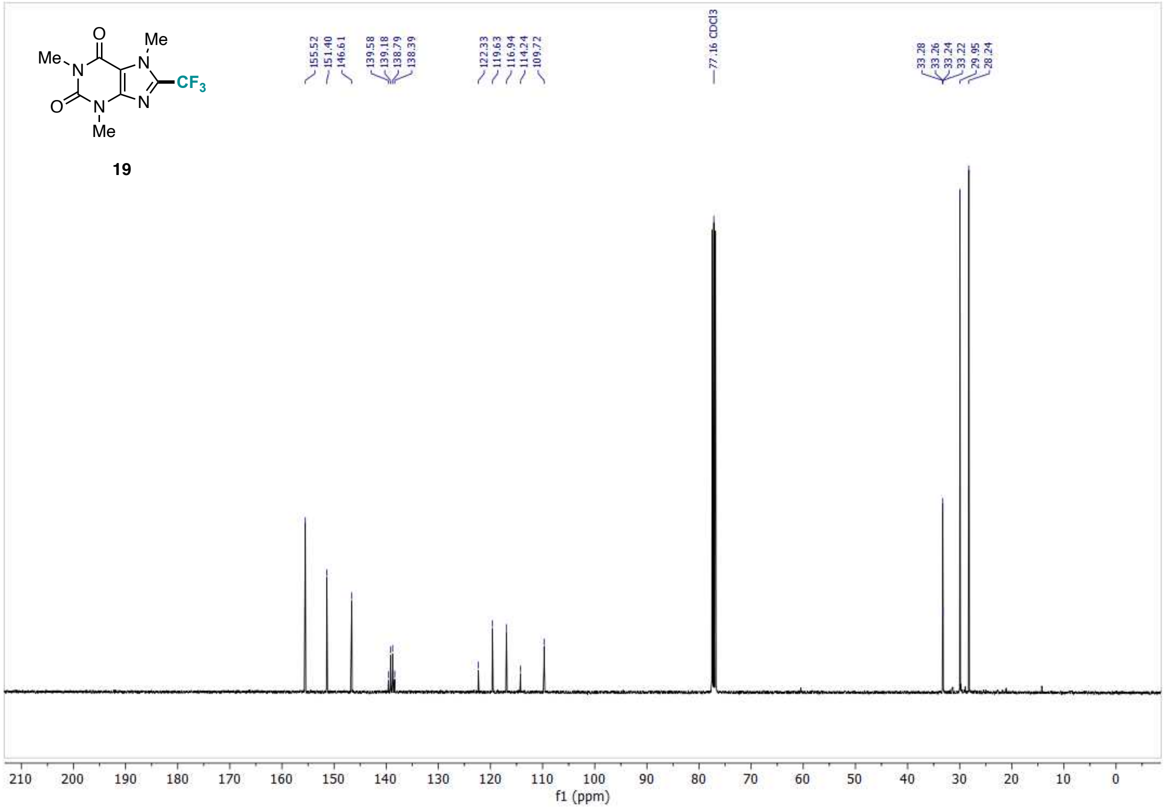

^{19}F-NMR (376 MHz, $CDCl_3$) of 19

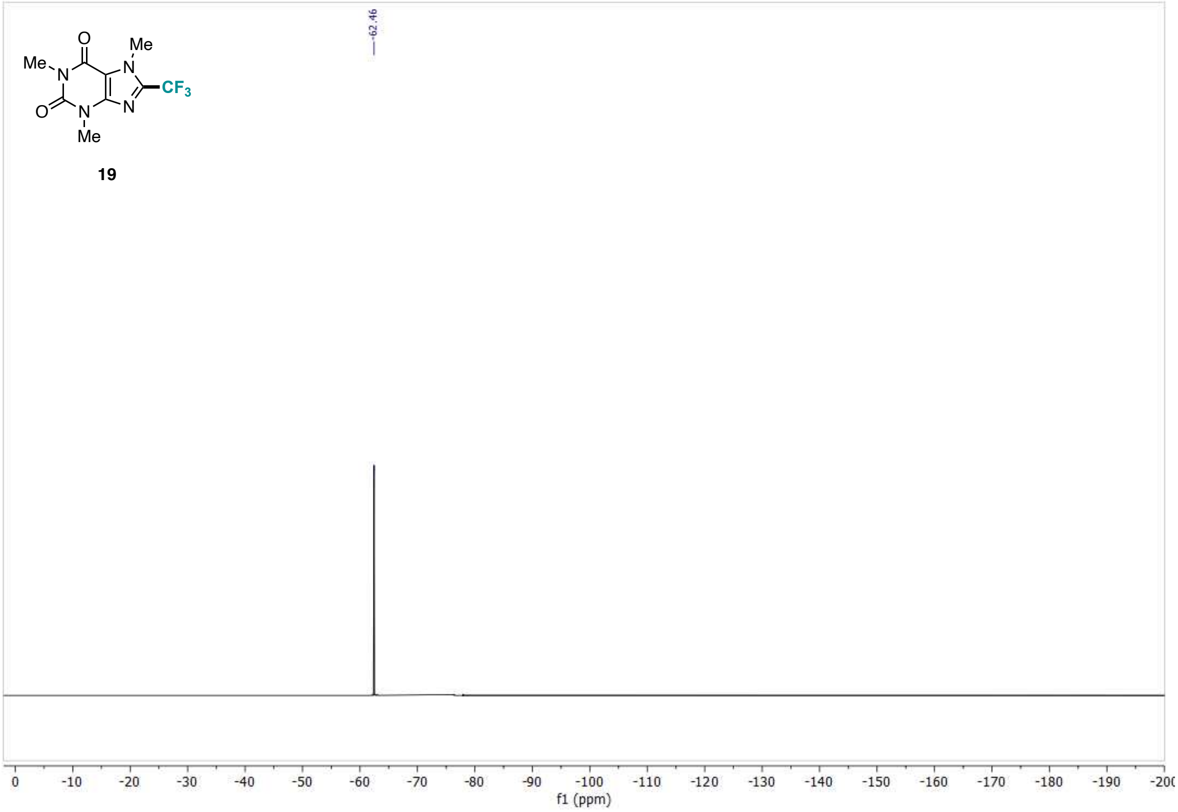

^{1}H-NMR (400 MHz, $CDCl_3$) of 20

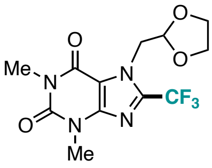

20

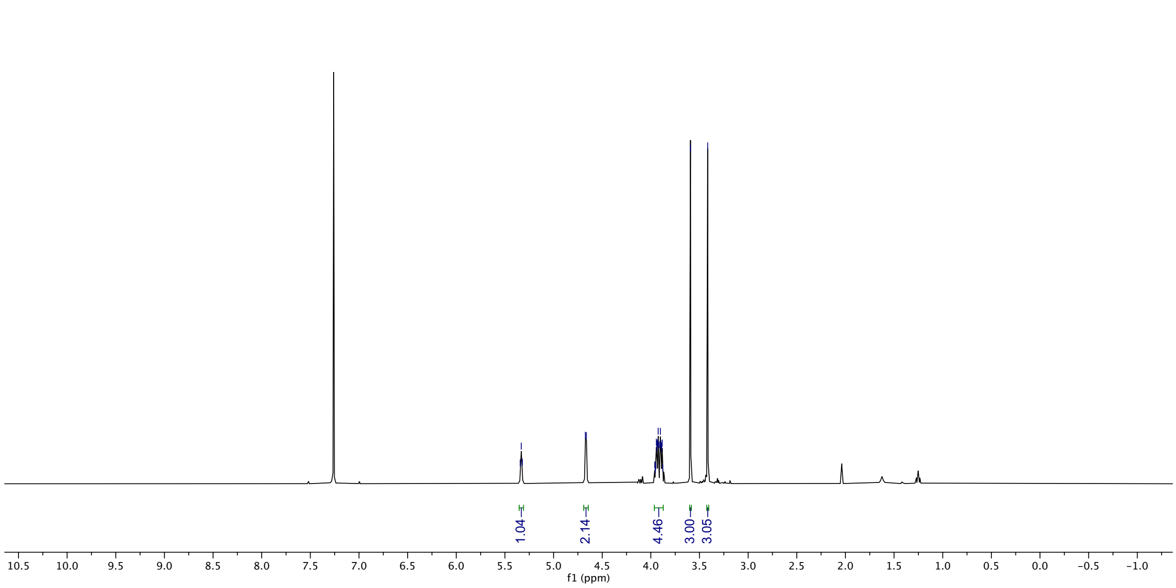

^{13}C-NMR (101 MHz, $CDCl_3$) of 20

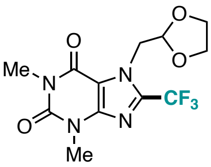

20

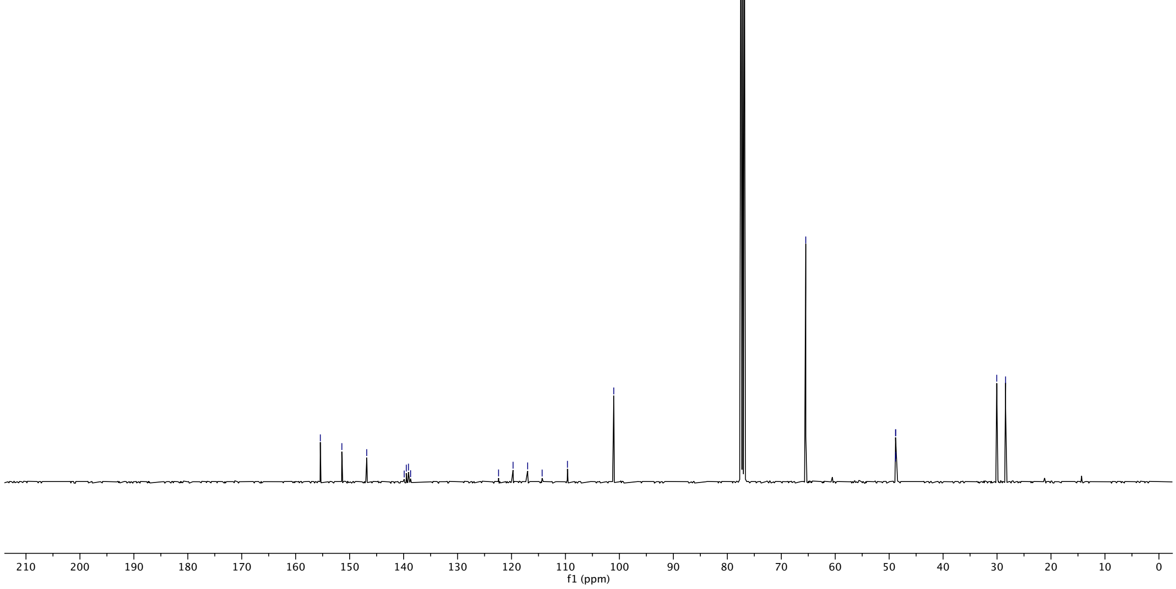

^{19}F-NMR (376 MHz, $CDCl_3$) of 20

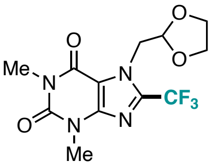

20

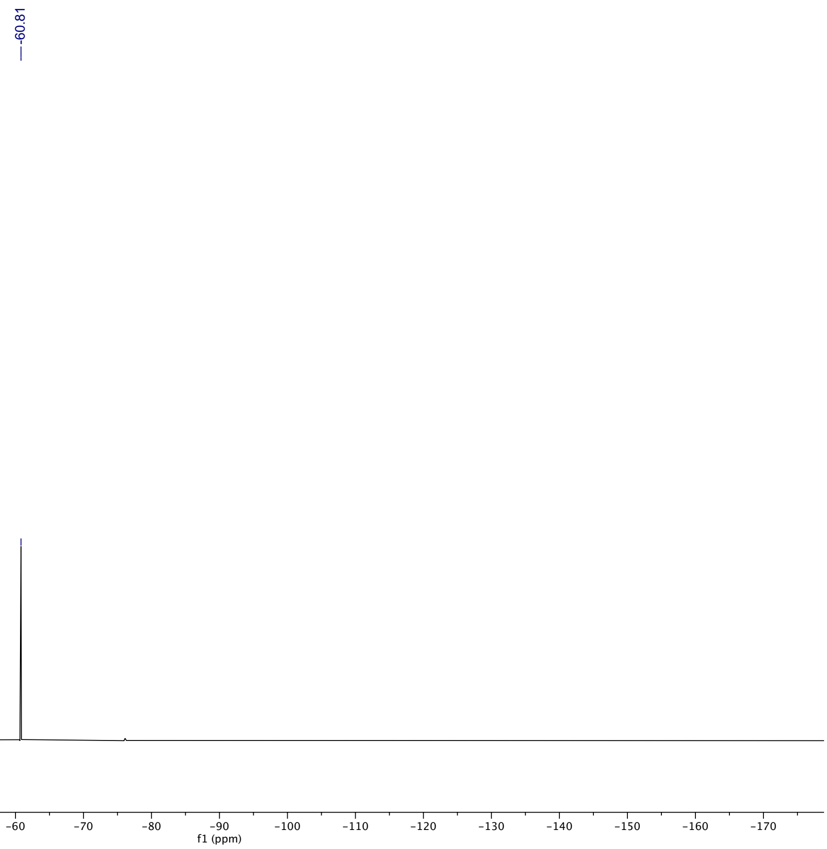

^{1}H-NMR (600 MHz, DMSO-d_6) of 21

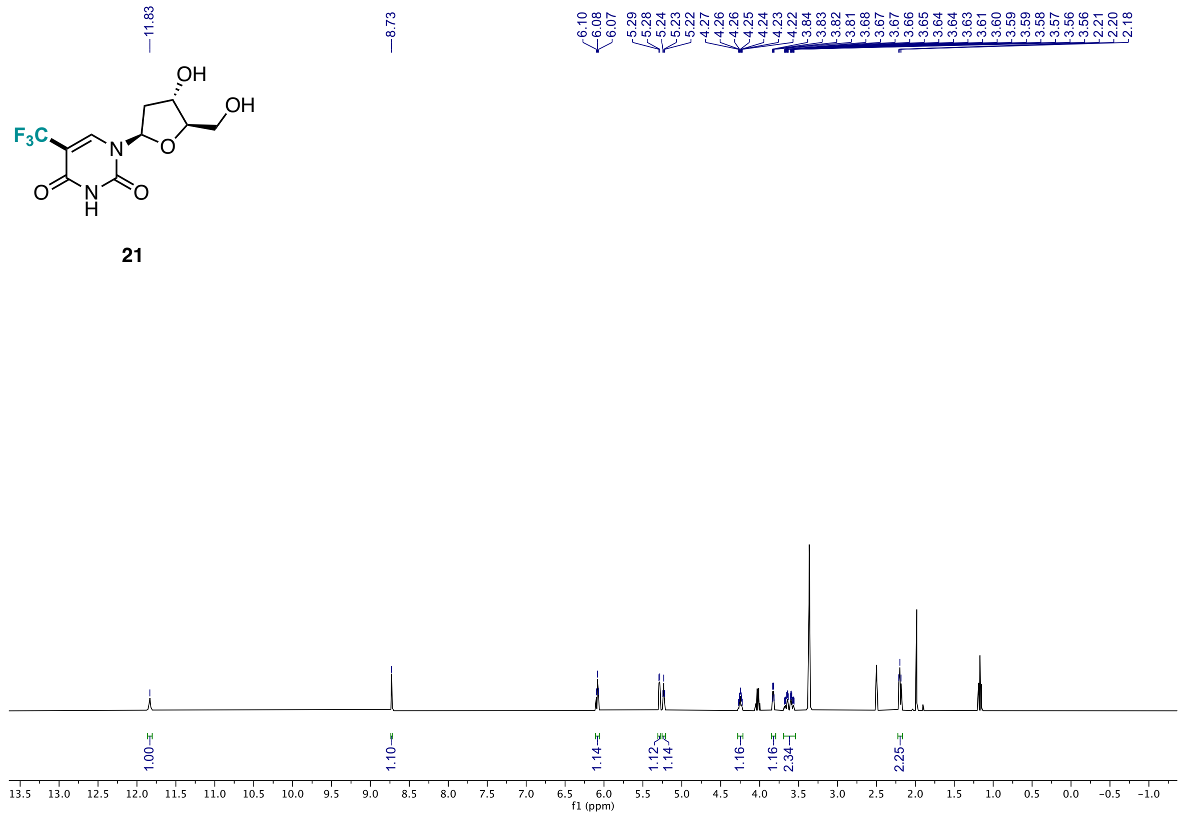

^{13}C-NMR (151 MHz, DMSO-d_6) of 21

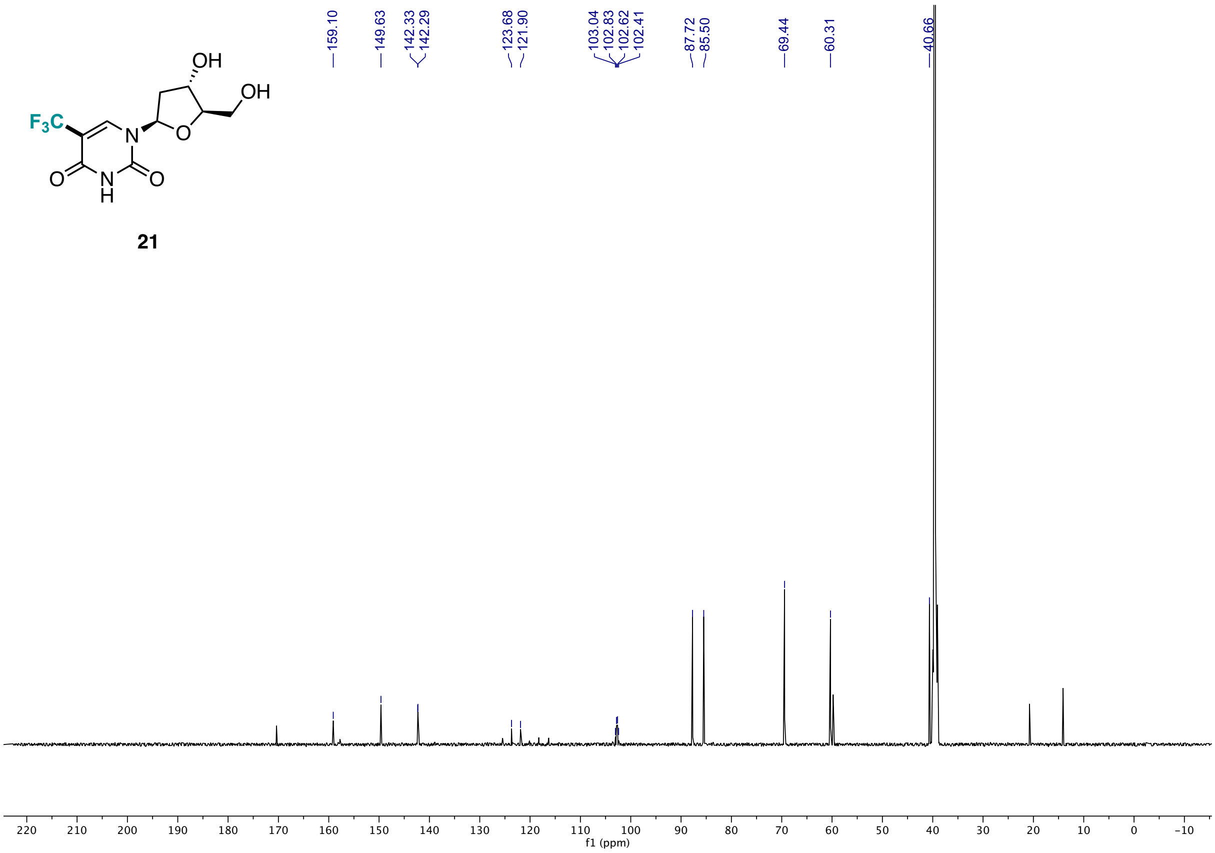

^{19}F-NMR (376 MHz, DMSO-d_6) of 21

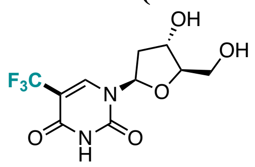

21

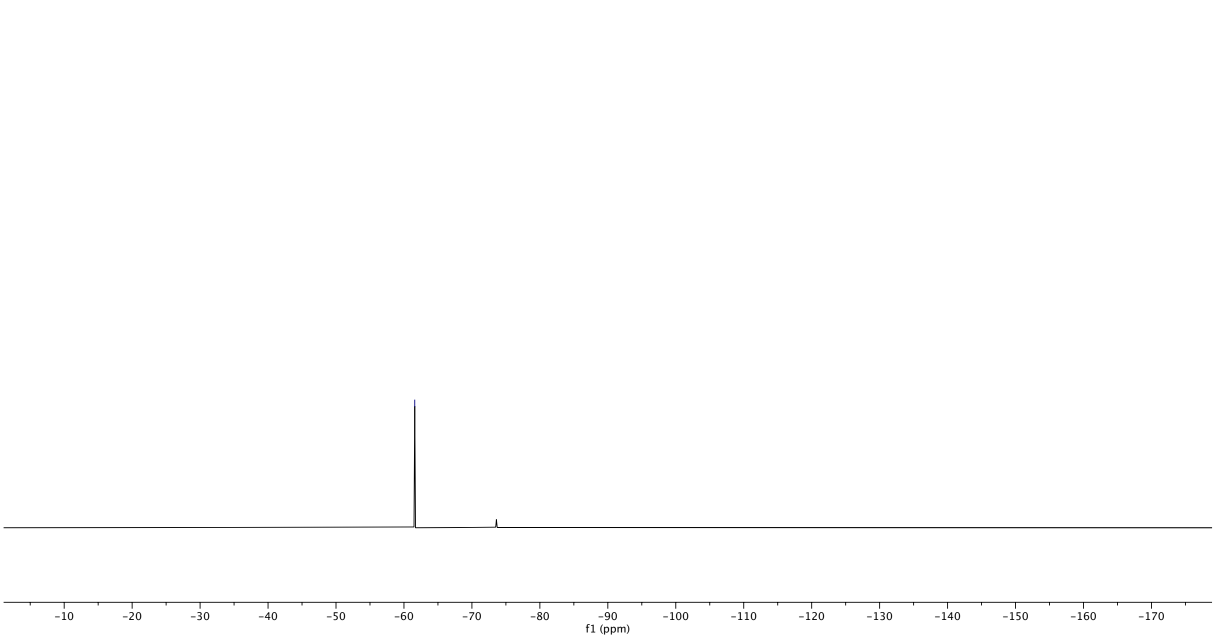

^{1}H-NMR (400 MHz, $CDCl_3$) of 22a + 22b

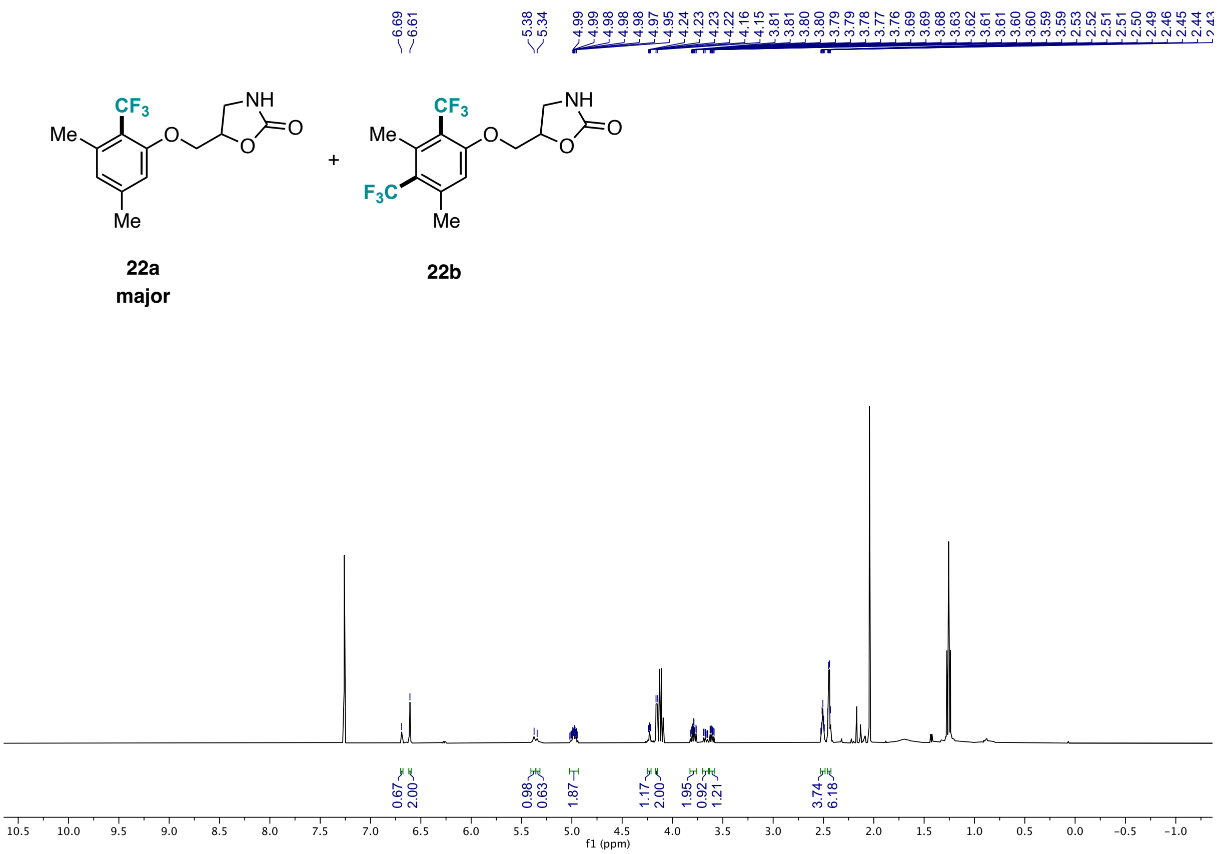

^{13}C-NMR (75 MHz, $CDCl_3$) of 22a + 22b

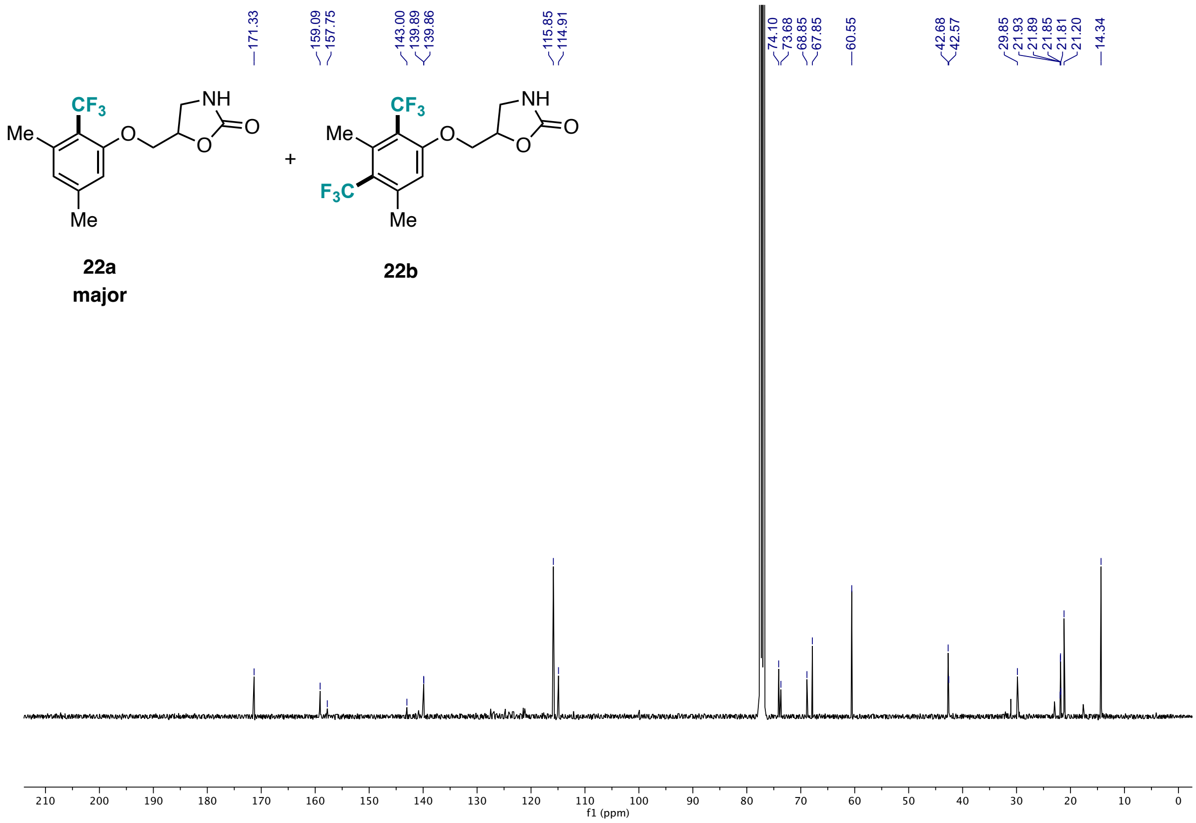

^{19}F-NMR (376 MHz, $CDCl_3$) of 22a + 22b

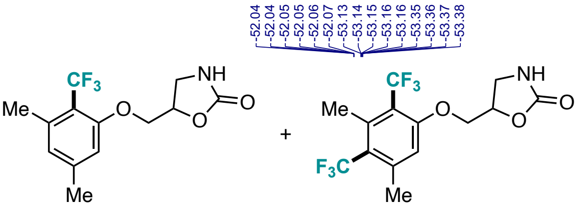

22a
major

22b

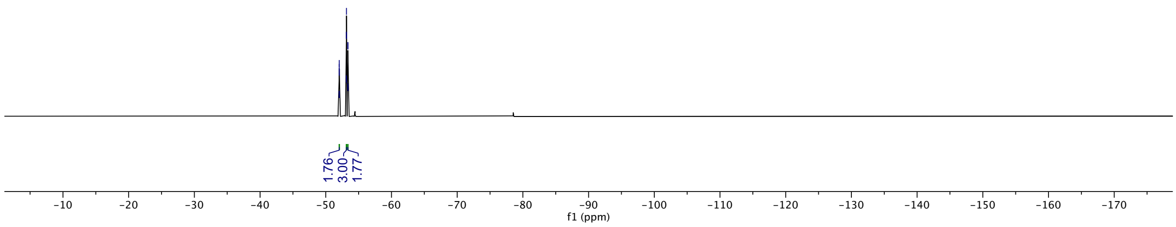

^{1}H-NMR (400 MHz, $CDCl_3$) of 23a

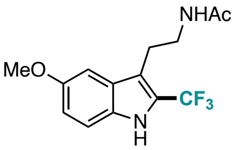

23a

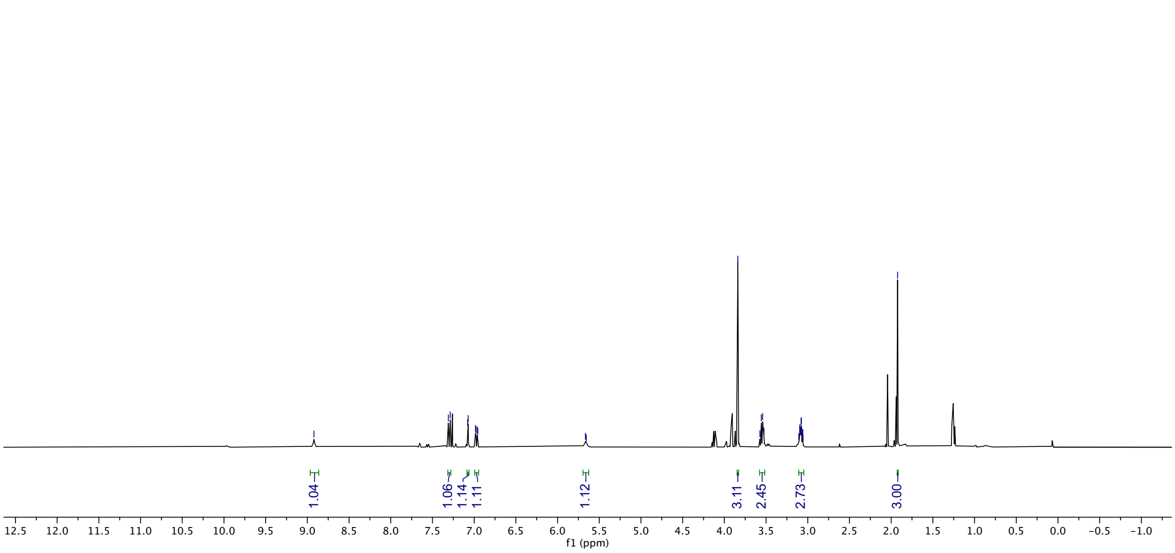

^{13}C-NMR (101 MHz, $CDCl_3$) of 23a

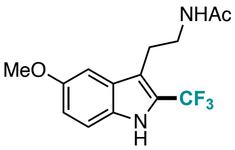

23a

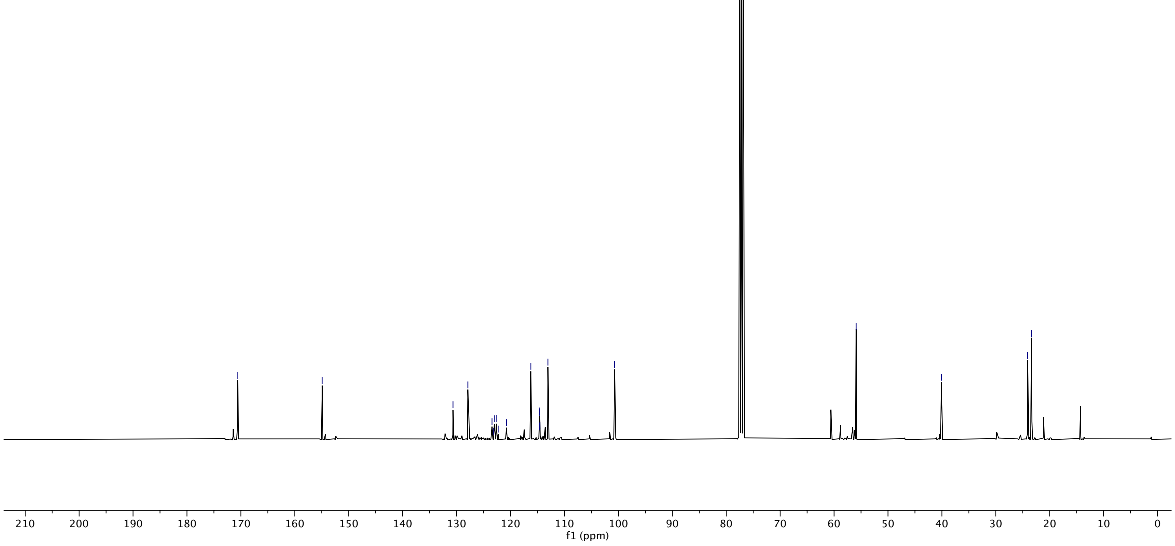

^{19}F-NMR (376 MHz, $CDCl_3$) of 23a

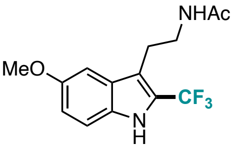

23a

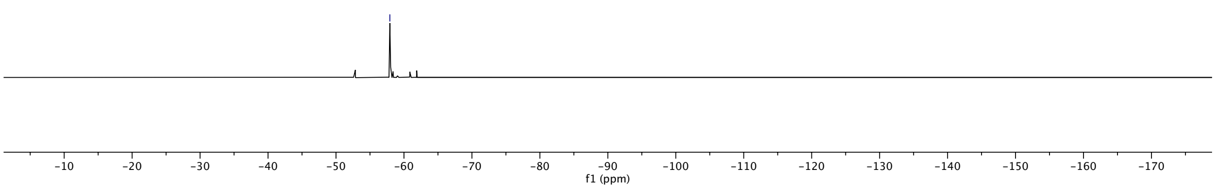

^{1}H-NMR (400 MHz, $CDCl_3$) of 23b + 23c

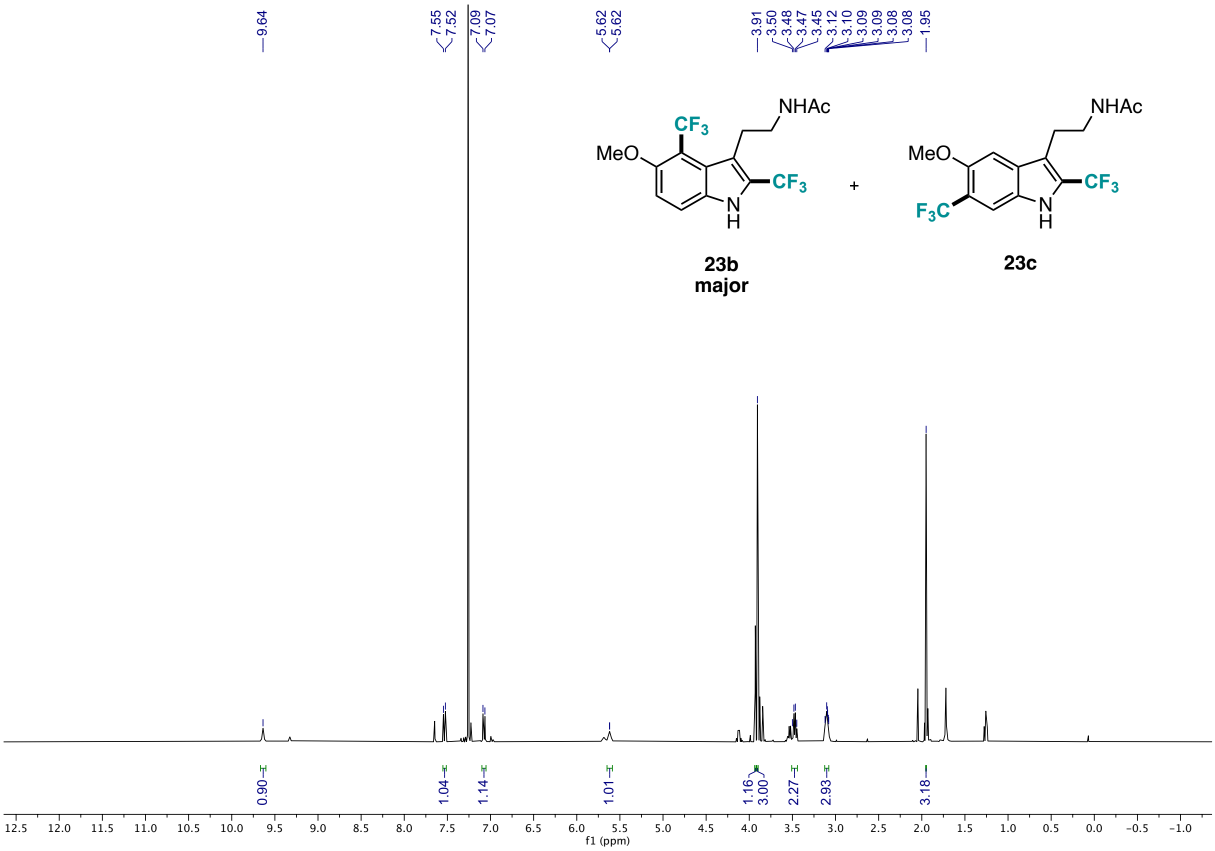

^{13}C-NMR (101 MHz, $CDCl_3$) of 23b + 23c

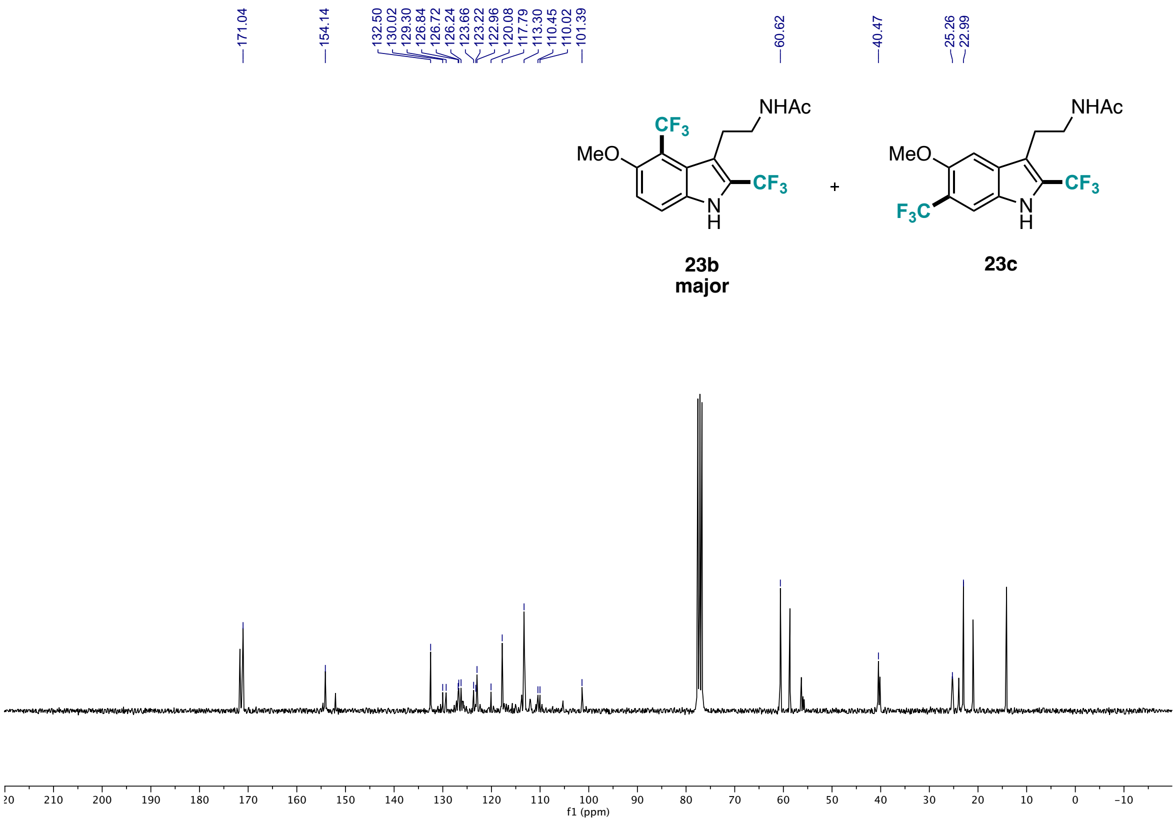

^{19}F-NMR (376 MHz, CDCl$_3$) of 23b + 23c

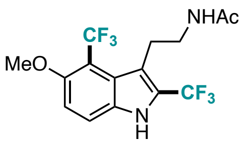

23b
major

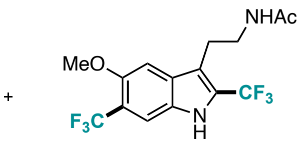

23c

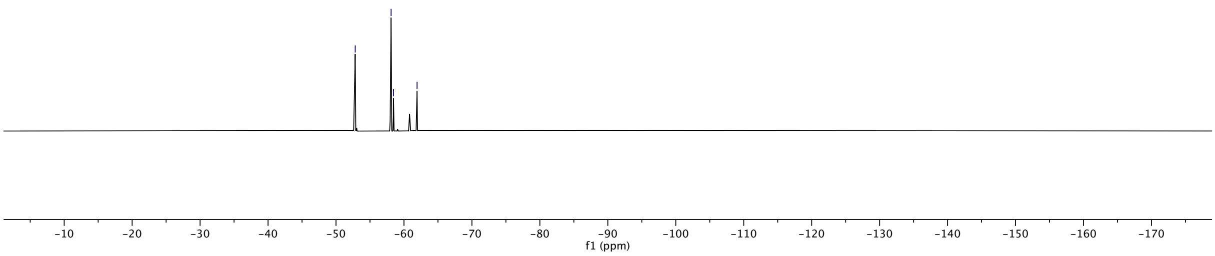

REFERENCES

(1) Yin, D.; Su, D.; Jin, J. Photoredox Catalytic Trifluoromethylation and Perfluoroalkylation of Arenes Using Trifluoroacetic and Related Carboxylic Acids. *Cell Rep. Phys. Sci.* **2020**, *1* (8), 100141.

(2) Kirij, N. V.; Filatov, A. A.; Khrapach, G. Yu.; Yagupolskii, Y. L. The First Nucleophilic C–H Perfluoroalkylation of Aromatic Compounds via (Arene)Tricarbonylchromium Complexes. *Chem. Commun.* **2017**, *53* (13), 2146–2149.

(3) Fernández-García, S.; Chantzakou, V. O.; Juliá-Hernández, F. Direct Decarboxylation of Trifluoroacetates Enabled by Iron Photocatalysis. *Angew. Chem. Int. Ed.* **2024**, *63,* e202311984.

(4) Nagib, D. A.; MacMillan, D. W. C. Trifluoromethylation of Arenes and Heteroarenes by Means of Photoredox Catalysis. *Nature* **2011**, *480* (7376), 224–228.

(5) Tsuruta, T.; Spinnato, D.; Moon, H. W.; Leutzsch, M.; Cornella, J. Bi-Catalyzed Trifluoromethylation of C(sp^2)–H Bonds under Light. *J. Am. Chem. Soc.* **2023**, *145* (47), 25538–25544.

(6) Torti, E.; Protti, S.; Fagnoni, M. *N*-Aryltrifluoromethanesulfonimides as New Trifluoromethylating Agents for the (Photo)Catalyst-Free Functionalization of (Hetero)Aromatics. *Chem. Commun.* **2018**, *54* (33), 4144–4147.

(7) Ackermann, L.; Motornov, V.; Trienes, S.; Resta, S.; Oliveira, J. C. A.; Lin, Z.; Liu, Z.; Von Münchow, T. Photoelectrochemical Iron(III)-Catalysis for Late-Stage C–H Fluoroalkylations. *Angew. Chem. Int. Ed.* **2025**, *64*, e202504143.

(8) Ji, Y.; Brueckl, T.; Baxter, R. D.; Fujiwara, Y.; Seiple, I. B.; Su, S.; Blackmond, D. G.; Baran, P. S. Innate C-H Trifluoromethylation of Heterocycles. *Proc. Natl. Acad. Sci. U.S.A.* **2011**, *108* (35), 14411–14415.

(9) Pavlishchuk, V. V.; Addison, A. W. Conversion Constants for Redox Potentials Measured versus Different Reference Electrodes in Acetonitrile Solutions at 25°C. *Inorganica Chimica Acta* **2000**, *298* (1), 97–102.

(10) Bard, A. J.; Faulkner, L. R. *Electrochemical Methods: Fundamentals and Applications*, 2nd ed.; Wiley: New York, 2001.

(11) Little, R. D. A Perspective on Organic Electrochemistry. *J. Org. Chem.* **2020**, *85* (21), 13375–13390.

(12) Francke, R.; Little, R. D. Redox Catalysis in Organic Electrosynthesis: Basic Principles and Recent Developments. *Chem. Soc. Rev.* **2014**, *43* (8), 2492.
